# Measuring the diagnostic management and follow‐up imaging for glioma patients across Belgian hospitals between 2016 and 2019

**DOI:** 10.1002/cam4.70045

**Published:** 2024-10-30

**Authors:** Dimitri Vanhauwaert, Katrijn Vanschoenbeek, Frank Weyns, Ludo Vanopdenbosch, Ann Tieleman, Alex Michotte, Karolien Goffin, Cindy De Gendt, Steven De Vleeschouwer, Tom Boterberg

**Affiliations:** ^1^ Department of Neurosurgery AZ Delta Roeselare Belgium; ^2^ Belgian Cancer Registry Brussels Belgium; ^3^ Department of Neurosurgery Ziekenhuis Oost‐Limburg Genk Belgium; ^4^ Neurosciences, Faculty of Medicine and Life Sciences Hasselt University Hasselt Belgium; ^5^ Department of Neurology AZ Sint‐Jan Brugge Bruges Belgium; ^6^ Department of Radiology AZ Delta Roeselare Belgium; ^7^ Department of Pathology (neuropathology) and Neurology UZ Brussel Brussels Belgium; ^8^ Department of Nuclear Medicine UZ Leuven Leuven Belgium; ^9^ Department of Neurosurgery UZ Leuven Leuven Belgium; ^10^ Department Neurosciences and Leuven Brain Institute (LBI) KU Leuven Leuven Belgium; ^11^ Department of Radiation Oncology Ghent University Hospital Ghent Belgium

**Keywords:** brain tumors, diagnostic management, follow‐up imaging, glioma, quality of care

## Abstract

**Objectives:**

This study aimed to assess the diagnostic management and follow‐up imaging for glioma patients across Belgian hospitals by calculating process indicators.

**Methods:**

Patients with newly diagnosed glioma in Belgium (2016–2019) were selected from the Belgian Cancer Registry. The National Social Security Number served as unique patient identifier, linking the Registry to vital status and reimbursement data. Nine measurable process related to diagnosis and follow‐up imaging were identified, with reformulations for 7 due to data limitations. For each indicator, technical documentation sheets, containing all required details (rationale, numerator and denominator, target, limitations, benchmarking, subgroup analyses) were developed, reviewed by a multidisciplinary expert panel, and validated in six pilot hospitals. Per indicator, patients were assigned to the most relevant hospital per indicator using allocation algorithms.

**Results:**

Results for process indicators assessing MRI use in glioma diagnosis and follow‐up aligned with predefined targets (90%), except for early postoperative MRI (48.5% vs. target 90%). Mandatory reporting of the WHO performance status (89.3% vs. target 100%) and performance of full‐spine (43.6% vs. target 90%) and follow‐up MRI (73.5% vs. target 90%) in ependymoma were suboptimal. The largest variability across centers was noted for the indicator on early postoperative MRI.

**Conclusion:**

This calculation of process indicators identified opportunities for improvement in diagnosis and follow‐up imaging for glioma patients in Belgium. Monitoring indicator results and providing individual feedback reports to the Belgian hospitals invites neuro‐oncology care teams and hospital managements to reflect on their results and to take measures to continuously improve care for glioma.

## Graphical abstract

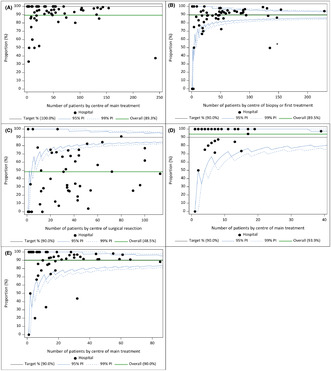

## 1 INTRODUCTION

Assessing quality of care through calculation of process and outcome indicators is widely performed in different oncological fields, but experience in neuro‐oncology is scarce.^1^, ^2^, ^3^, ^4^ Though Denmark and Scotland have used process and outcome indicators to improve care for neuro‐oncology patients.^5^, ^6^, ^7^ Recently, in Belgium, a set of process and outcome indicators to illuminate care paths was developed for neuro‐oncology. By calculating these indicators, the quality of care for glioma patients can be mapped and monitored over time.^8^

The objective of this study was to calculate process indicators assessing the quality of *diagnostic management* and *follow‐up imaging* in patients diagnosed with glioma across Belgian hospitals from 2016 to 2019. Although presenting hospital‐level results in funnel plots allows for benchmarking, this first assessment should be considered exploratory. It can affirm both physicians and institutions to continue their good clinical practice or, on the contrary, identify opportunities for improvement in certain domains of the care path. Where feasible, comparisons with earlier time frames (2009–2011 and 2012–2015) were conducted to discern evolving trends. Subsequent publications will elaborate on the patterns and quality of therapeutic care (including surgery, chemo‐ and/or radiotherapy), quality of pathology reporting, and mortality and survival.

## 2 MATERIALS AND METHODS

### 2.1 Data sources and study cohort

In Belgium, cancer registration is compulsory, and all new diagnoses ought to be reported to the Belgian Cancer Registry (BCR) by the so‐called “oncological care programs” of the Belgian hospitals and independently also by the laboratories of pathological anatomy. Required data include incidence date, basis of diagnosis (e.g., autopsy, histology of primary tumor, technical exam, etc.), ICD‐O‐3 (International Classification of Diseases for Oncology, 3rd edition) topography and morphology code, and WHO performance status at time of diagnosis.^10^ The BCR is legally charged with the collection of data on all new oncological diseases, and the completeness of the database is estimated at least 98% as from 2004 onwards.^11^, ^12^ This study focused on all newly diagnosed glioma (ICD‐O‐3: 938‐945/C71) between 2016 and 2019. This cohort included patients of 18 years or older with official residence in Belgium at time of diagnosis and with a known National Social Security Number (NSSN). Exclusion criteria were subsequently applied to exclude patients lacking reimbursement data for diagnostic or therapeutic procedures within the time frame 1 month before until 3 months after incidence date, cases where the incidence date coincided the date of death and patients lost to follow‐up from the day of incidence. The NSSN was used as a unique identifier to link the BCR database with other data sources. First, the database of the Intermutualistic Agency (IMA) provided details on all diagnostic and therapeutic procedures reimbursed by the compulsory health insurance. Linkage was performed with cancer‐related diagnostic and therapeutic procedures occurring as from January 1st of the year preceding the incidence year up to December 31st of the fifth year after the incidence year. However, a delay up to 2 years should be considered. Since an administrative database lacks the specific diagnosis related to or the indication for procedures, time frames were established around the incidence date for all extracted procedure codes. This approach aims to increase the likelihood that these procedures were related to the glioma. Second, the vital status of patients was retrieved from the Crossroad Bank of Social Security. An approval for linkage of the databases is provided by the Data Protection Authority (DPA), previously known as the Belgian Privacy Commission.^13^

### 2.2 Process indicators

From the previously published list of indicators assessing care for patients diagnosed with glioma, 11 process indicators pertaining to the diagnostic management and follow‐up imaging were identified (see Table 1).^8^ Measurability was judged based on the ability to define every single element of the indicator based on the available data. To address shortcomings in the available data, some indicators required reformulation to be measurable. In case insufficient data were available, recommendations for additional or more specific future data collection were suggested. In line with the methodology used by the KCE (Belgian Knowledge Center for Healthcare) in indicator projects, for each indicator a Technical Documentation Sheet (TDS) was developed, detailing the rationale for the indicator, eventual reformulations, the numerator and denominator of the calculation, the target value, the data source(s) and technical definitions used to define every single element of the indicator, risk adjustment if indicated, limitations, subgroup analyses, sensitivity analyses, benchmarking, and comparable international results if available.^14^, ^15^, ^16^, ^17^, ^18^ The TDSs can be found in Appendix S1. Prior to definite calculation of the indicators, these TDSs were reviewed by a multi‐disciplinary expert board. In order to assess concordance between diagnostic and therapeutic procedures identified in the project‐specific database (cancer registration linked to administrative data) and the information directly available in the hospitals (i.e., medical files and financial data, which are considered as “gold standard”), a validation study and data checks were performed in 6 pilot hospitals.

**TABLE 1:** Initial process indicators related to diagnostic management and follow‐up imaging, reformulation based on availability of data and limitations.

QI	Initial Quality Indicator	Reformulation	Benchmarking	Target	Calculation	Limitations (availability of data in administrative databases)
1	Proportion of newly diagnosed glioma patients who have a documented WHO performance status or KPS at time of multidisciplinary team (MDT) discussion	**Proportion of glioma patients who have a WHO performance status reported to the BCR**	Center of main treatment	100%	N: glioma patients who have a WHO performance status reported to the BCR	Only WHO performance score is available, KPS not
					D: all glioma patients	Only WHO score at cancer registration is available, not necessarily the same at MDT
2	Proportion of patients with newly diagnosed glioma who are discussed at multidisciplinary team after obtaining pathological diagnosis prior to definitive management (chemotherapy, radiotherapy, second look surgery or resection after initial biopsy).	**Proportion of glioma patients who are discussed at a multidisciplinary team (MDT) meeting between 1 month before incidence date and 9 months after incidence date**	n.a. (descriptive indicator)	n.a.	N: glioma patients with WHO performance status reported to the BCR	Timing of MDT (in relation to definitive treatment) is uncertain due to reimbursement rules (1 MDT/year)
					D: all glioma patients	
3	Proportion of patients with suspected brain tumor who underwent MRI with T2, T1 and T1 contrast enhanced weighted images.	**Proportion of glioma patients who underwent MRI (MRI brain or fMRI) before a diagnostic biopsy or before start of treatment in the absence of a diagnostic biopsy**	Center of biopsy or first treatment (in absence of biopsy)	90%	N: glioma patients who underwent MRI (MRI brain or fMRI) from 6 weeks before until 1 day before diagnostic biopsy or before start of oncological treatment in the absence of a diagnostic biopsy	Only confirmed tumors are registered, suspected tumors cannot be identified
					D: glioma patients who received a diagnostic biopsy and/or oncological treatment (surgical resection, chemotherapy and/or (chemo)radiotherapy)	Different MRI sequences cannnot be identified
4	Proportion of patients with suspected low‐grade glioma in whom nuclear imaging with amino acid PET was incorporated in defining target for biopsy.	**Proportion of patients with low‐grade (grade 2) glioma in whom nuclear imaging with PET was performed before a diagnostic biopsy**	n.a. (descriptive indicator)	n.a.	D: low‐grade glioma patients undergoing PET from 12 weeks before until the day before a diagnostic biopsy	Only confirmed tumors are registered, suspected tumors cannot be identified
						Incorporation in biopsy planning and targeting cannot be identified
					N: low‐grade (grade 2) glioma patients who underwent a diagnostic biopsy	Identification of used radiopharmaceuticals is not possible for all cases
						Absence of clear guideline makes target setting arbitrary
5	**Proportion of patients with diagnosis of intracranial ependymoma who had full spine MRI**	n.a.	Calculated only at population level	n.a.	N: intracranial ependymoma patients who received (full) spine MRI from 1 month before until 6 weeks after incidence date	Differentiation between full spine MRI vs. MRI for cervical, thoracic or lumbosacral spine only possible from december 2018
					D: intracranial ependymoma patients	
6	Proportion of patients with low‐grade glioma undergoing follow‐up with MRI 2 to 4 times yearly for the first 5 years after diagnosis. MRI should include T2/flair and T1 with and without contrast and volumetric assessment of residual tumor	**Proportion of patients with** low‐grade **(grade 2) glioma undergoing at least two MRI's (MRI brain or fMRI) in the first year of follow‐up**	Center of main treatment	90%	N: low‐grade (grade 2) glioma patients with at least 1 year of follow‐up in IMA‐data undergoing at least two MRI's (MRI brain or fMRI) in the first follow‐up year	Different MRI sequences cannnot be identified
					D: low‐grade (grade 2) glioma patients with at least 1 year of follow‐up in IMA‐data (follow‐up starting from start date of oncological treatment or from day of diagnostic biopsy in the absence of oncological treatment)	Volumetric assessment of residual tumor cannot be identified
7	(*Proportion of patients with* low‐grade *glioma undergoing followup with MRI 1 to 2 times yearly after the first 5 years after diagnosis. MRI should include T2/flair and T1 with and without contrast and volumetric assessment of residual tumor*)	n.a.	Not measurable	n.a.		Administrative data only available for BCR until 5th year after incidencec year
8	Proportion of patients with high‐grade glioma undergoing follow‐up with MRI with volumetric assessment of residual tumor every 2 to 4 months	**Proportion of patients with** high‐grade **(grade 3/4) glioma undergoing at least three MRI's (MRI brain or fMRI) in the first year of follow‐up**	Center of main treatment	90%	N: high‐grade (grade 3/4) glioma patients with at least 1 year of follow‐up in IMA‐data undergoing at least three MRI's (MRI brain or fMRI) in the first follow‐up year	Different MRI sequences cannot be identified
					D: high‐grade (grade 3/4) glioma patients with at least 1 year of follow‐up in IMA‐data (follow‐up starting from start date of oncological treatment or from day of diagnostic biopsy in the absence of oncological treatment)	Volumetric assessment of residual tumor cannot be identified
						Administrative data only available for BCR until 5th year after incidence year
9	**Proportion of patients with ependymoma undergoing follow‐up with MRI with volumetric assessment of residual tumor every 3 months for the first 2 years after diagnosis**	n.a.	Calculated only at population level	n.a.	N: ependymoma patients with at least 1 year of follow‐up in IMA‐data undergoing at least three MRI's (MRI brain or fMRI) in the first follow‐up year	Volumetric assessment of residual tumor cannot be identified
					D: ependymoma patients with at least 1 year of follow‐up in IMA‐data (follow‐up starting from start date of oncological treatment or from day of diagnostic biopsy in the absence of oncological treatment)	
10	(*Proportion of patients with ependymoma undergoing follow‐up with MRI with volumetric assessment of residual tumor every 6 months after the first 5 years after diagnosisi*	n.a.	Not measurable	n.a.		Volumetric assessment of residual tumor cannot be identified
						Administrative data only available for BCR until 5th year after incidence year
11	Proportion of patients with HGG who had postoperative MRI in which volumetric assessment of residual tumor is performed within 72 h of surgical resection	**Proportion of patients with** high‐grade **(grade 3/4) glioma who had postoperative MRI (MRI brain or fMRI) within 72 h after surgical resection**	Center of surgical resection	90%	N: high‐grade (grade 3/4) glioma patients who underwent a surgical resection undergoing a postoperative MRI (MRI brain or fMRI) performed within the first 3 days after surgical resection	Volumetric assessment of residual tumor cannot be identified
					D: low‐grade glioma patients undergoing PET from 12 weeks before until the day before a diagnostic biopsy	For MRI on day of surgery, differentiation between preoperative diagnostic or postoperative MRI is impossible

*Note*: Process indicators in bold were calculated, *Process indicators in italics* remained unmeasurable.

Abbreviations: D, denominator; N, numerator.

### 2.3 Assignment of each patient to one center

To benchmark indicators between hospitals, it was crucial to identify in which hospital(s) patients received their diagnostic and therapeutic care. Per indicator, each patient had to be assigned to one center.^9^, ^14^, ^15^, ^16^, ^17^, ^18^ As patients often received care in more than one hospital, allocation algorithms were designed in order to identify the hospital with the most important impact on a certain indicator. For the indicators under investigation in this study, benchmarking between hospitals was based on either ‘center of main treatment’, ‘center of biopsy’, ‘center of surgical resection’ or ‘center of biopsy or first treatment’. The main treatment center is the facility that, in descending order of priority, provides surgical resection, radiotherapy, chemotherapy, or, if none of the aforementioned treatments are available, a biopsy. The detailed allocation algorithms including applied priority rules can be found in Appendix S2. For hospitals mergers, the fusion status was considered as of December 31st, 2009. This means that if two hospitals merged before this date, the results of both hospitals were combined. When two hospitals merged after this date, the results of both hospitals were calculated and presented separately.

### 2.4 Statistical analysis

For all process indicators, population‐level results for the main cohort (incidence years 2016–2019) are calculated and compared with earlier cohorts (incidence years 2012–2015 and 2008–2011). Descriptive presentations without statistical tests were employed. For the indicators with a sufficient number of patients in the denominator, the observed indicator results per hospital are visualized in funnel plots. The estimate of an indicator is plotted on the vertical axis versus the number of observations (patients in the denominator) per hospital on the x‐axis. The agreed target is used as reference value and added to the plot, as well as assumed prediction limits (95 and 99%). Also, the observed overall (national) indicator result is presented.^14^ The 95% prediction limits are the 2.5% and 97.5% quantiles of the binomial distribution around the central value (target) for a given center size. These were calculated in SAS using the quantile function.

## 3 RESULTS

### 3.1 Cohort description and patient to center assignment

A total of 3137 gliomas, newly diagnosed between 2016 and 2019, were selected from the BCR database, corresponding to 3136 patients. In total 70 gliomas (2.2%) were excluded: for 67 (2.1%) there were no links with the health insurance database (IMA) and for 3 (0.1%) the incidence date corresponded with the day of death. This resulted in 3067 remaining tumors in this study set.

Almost 60% (*n* = 1838) of the included patients were men. Twenty‐six point six percent (*n* = 816) of patients were diagnosed in the 7th decade of life, while 33.2% (*n* = 1017) of patients were 70 years or older. The vast majority (*n* = 1906; 62.2%) presented with a WHO performance status of 1, that is, ‘Symptomatic but completely ambulatory’. Only 176 patients (5.7%) were asymptomatic at diagnosis (WHO score of 0), while the WHO performance status was missing in 329 patients (10.7%). For 2924 gliomas (95.3%), the basis for diagnosis was histology of the primary tumor, while for 143 (4.7%) diagnosis was made based on technical investigations (imaging) only. The majority of patients (*n* = 2270; 74.0%) were diagnosed with a glioblastoma, while 218 (7.1%) had a grade 2 astrocytoma and 159 (5.2%) an anaplastic astrocytoma. Patient and tumor characteristics can be found in Table 2 and Table 3. The assignment of patients to hospitals according to allocation algorithms can be found in Table 4. Biopsies were performed in 50 centers and surgical resection in 56. There were 73 centers of main treatment. Over 4 years, the median number of patients treated is 15 for biopsy centers, 31 for resection centers and 21 for main treatment centers. During the study period 2016–2019, four hospital fusions occurred.

**TABLE 2:** Patient characteristics of the study population.

Glioma (2016–2019) *N* = 3067	Nr of patients	% of patients
Year of incidence		
2016	752	24.5
2017	742	24.2
2018	749	24.4
2019	824	26.9
Age at diagnosis		
18–29 years	141	4.6
30–39 years	219	7.1
40–49 years	324	10.6
50–59 years	550	17.9
60–69 years	816	26.6
70–79 years	696	22.7
80+ years	321	10.5
Sex		
Male	1838	59.9
Female	1229	40.1
WHO – Performance Status		
0 – Asymptomatic	176	5.7
1 – Symptomatic but completely ambulatory	1906	62.2
2 – Symptomatic. <50% in bed during the day	462	15.1
3 – Symptomatic. >50% in bed but not bedbound	153	5.0
4 – Bedbound	41	1.3
Missing	329	10.7
Diabetes*		
No	2717	88.6
Yes	350	11.4
Respiratory disease*		
No	2835	92.4
Yes	232	7.6
Cardiovascular disease*		
No	1718	56.0
Yes	1349	44.0
Multiple tumors**		
No	2764	90.1
Yes	303	9.9
*Outside the CNS*	*26*	*8.6*
*CNS*	*277*	*91.4*

* Identification of comorbidities (diabetes, respiratory disease and cardiovascular disease) is based on medication use in the year prior to the glioma diagnosis.

** Multiple tumors refer to the presence of additional tumors within a timeframe of 5 years before to maximum 2 years after the incidence date of the glioma diagnosis (CNS: benign, borderline or malignant tumor within the CNS – Outside the CNS: malignant tumor outside the CNS).

**TABLE 3:** Tumor characteristics of the study population.

Glioma (2016–2019) *N* = 3067			
Morfology/behavior	Glioma subtype	Nr of patients	% of patients
(ICDO‐3)			
Glioma (*N* = 67)			
9380/1	Borderline glioma	1	0,0
9380/3	Malignant glioma	65	2,1
9381/3	Gliomatosis cerebri	1	0,0
Mixed glioma (*N* = 6)			
9382/3	(Anaplastic) oligoastrocytoma. NOS	6	0,2
Diffuse midline glioma (*N* = 9)			
9385/3	Diffuse midline glioma H3 K27‐mutant**	9	0,3
Ependymoma (*N* = 39)			
9391/3	Ependymoma	32	1,0
9392/3	Anaplastic ependymoma	6	0,2
9393/3	Papillary ependymoma	1	0,0
Astrocytoma (*N* = 218)			
9400/3	Diffuse astrocytoma*	185	6,0
9411/3	Gemistocytic astrocytoma*	16	0,5
9420/3	Fibrillary astrocytoma*	17	0,6
Anaplastic astrocytoma (159)			
9401/3	Anaplastic astrocytoma**	159	5,2
Other type of astrocytoma (*N* = 108)			
9383/1	Subependymoma	43	1,4
9384/1	Subependymal giant cell astrocytoma	3	0,1
9421/1	Pilocytic astrocytoma	52	1,7
9424/3	(anaplastic) pleiomorphic xantroastrocytoma	9	0,3
9425/3	Pilomyxoid astrocytoma	1	0,0
Glioblastoma (*N* = 2270)			
9440/3	Glioblastoma**	2181	71,1
9441/3	Giant cell glioblastoma**	37	1,2
9442/3	Gliosarcoma**	36	1,2
9445/3	IDH‐mutant glioblastoma**	16	0,5
Oligodendroglioma (*N* = 191)			
9450/3	Oligodendroglioma*	112	3,7
9451/3	Anaplastic oligodendroglioma**	79	2,6

* Low‐grade gliomas.

** High‐grade gliomas.

**TABLE 4:** Allocation of patients and patient distribution for ‘center of biopsy’, ‘center of surgical resection’, ‘center of main treatment’ or ‘center of biopsy or first treatment’ for all patients diagnosed with gliomas, incidence years 2016–2019.

	Number of distinct known centers	Number of patients with center		Number of patients by center					
	*N*	Known	Unknown	Mean	Min	Q1	Median	Q3	Max
		*N* (%)	*N* (%)						
Center of biopsy	50	906 (100.0)	0 (0.0)	18,1	1	6	15	25	77
Center of surgical resection	56	2210 (99.9)	2 (0.1)	38.8	1	12	31	53	160
Center of main treatment	73	3059 (99.7)	8 (0.3)	41.4	1	4	21	54	242
Center of biopsy or 1st treatment	58	2921 (99.9)	2 (0.1)	49.5	1	13	42	74	229

### 3.2 Measurability of process indicators and validation

**FIGURE 1:**
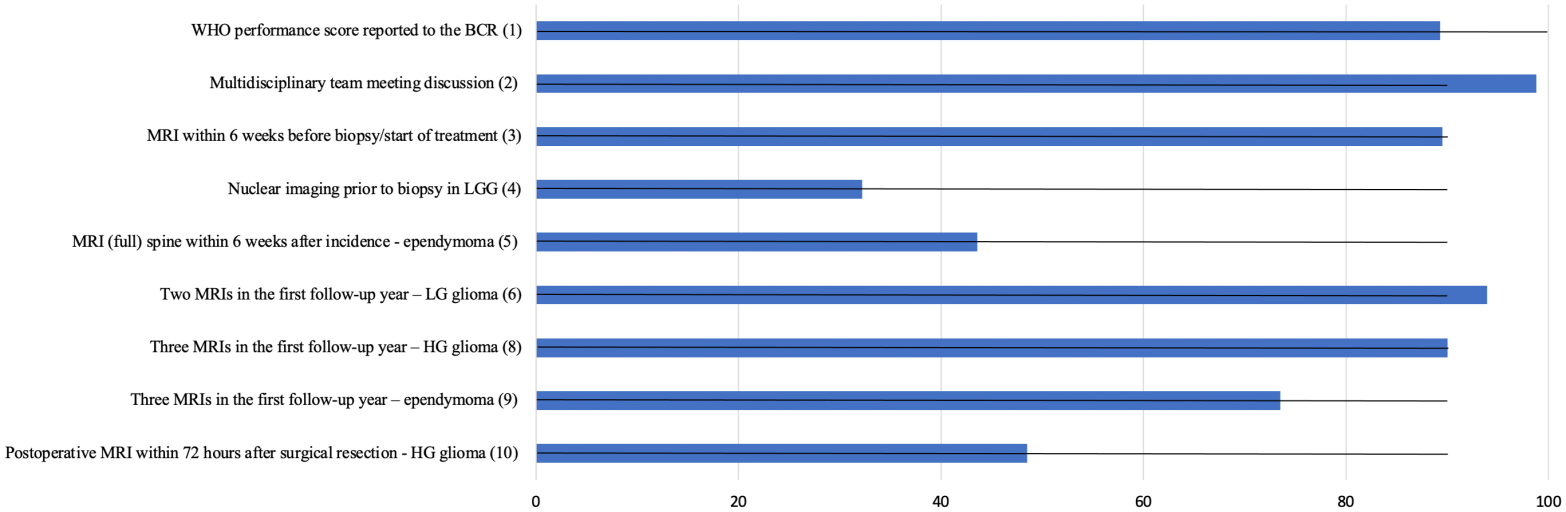
Overview of indicator results on population level. Targets are represented by horizontal lines.

As illustrated in Table 1, for 2 of the 11 process indicators, the initial formulation could be retained, while 7 process indicators needed rephrasing because of shortcomings in the available data, such as the impossibility to differentiate MRI sequences or to assess residual tumor volumes. For 2 process indicators (indicator 7 and 10) assessing follow‐up after the first 5 years after diagnosis, calculation was impossible since follow‐up time is currently too short and, even for the earliest incidence year, IMA‐data access authorization is still limited to the 5th year after the incidence year. In addition, for the indicator on *multidisciplinary team meeting* (MDT) discussion and the indicator on PET imaging prior to biopsy in low‐grade glioma (LGG), the multi‐disciplinary expert board responsible for the validation of the TDS and the target setting agreed that these indicators should be considered as pure descriptive indicators (i.e., without target, benchmarking, and funnel plot presentation). For those indicators only the national result is presented. Detailed calculation for all indicators including subgroup analysis, historical comparison, and sensitivity analysis can be found in Appendix S3. The results on population level and targets for all indicators are illustrated in Figure 1.

The validation phase revealed some discordances that could potentially had a small impact on the descriptive analyses, indicator results, and/or patient allocation. First, some procedures were not billed by hospitals or were billed using a (inappropriate) nomenclature code not included in the project selection. Second, some procedures (e.g., CT or MRI) were performed for other indications than for the diagnosis or treatment of the glioma. Besides that, some minor discordances without impact on the data set were encountered, such as small date deviations. Only changes that were applicable to the whole study cohort (= methodological changes) were implemented following this validation phase. No in‐ or exclusion of individual patients, punctual changes of tumor characteristics, or adaptations of procedures at individual level were considered, as it would lead to a bias in favor of the hospitals who participated to the validation. The concordance between the administrative data and the hospital data was considered good. More than 90% of the information as calculated/identified based on the cancer registry database and the IMA database was confirmed by the hospitals based on their information sources such as the medical files. The less than 10% discrepancies in information had no or only a very limited impact on the final indicator result.

### 3.3 Proportion of glioma patients who have a WHO performance status reported to the BCR

**FIGURE 2:**
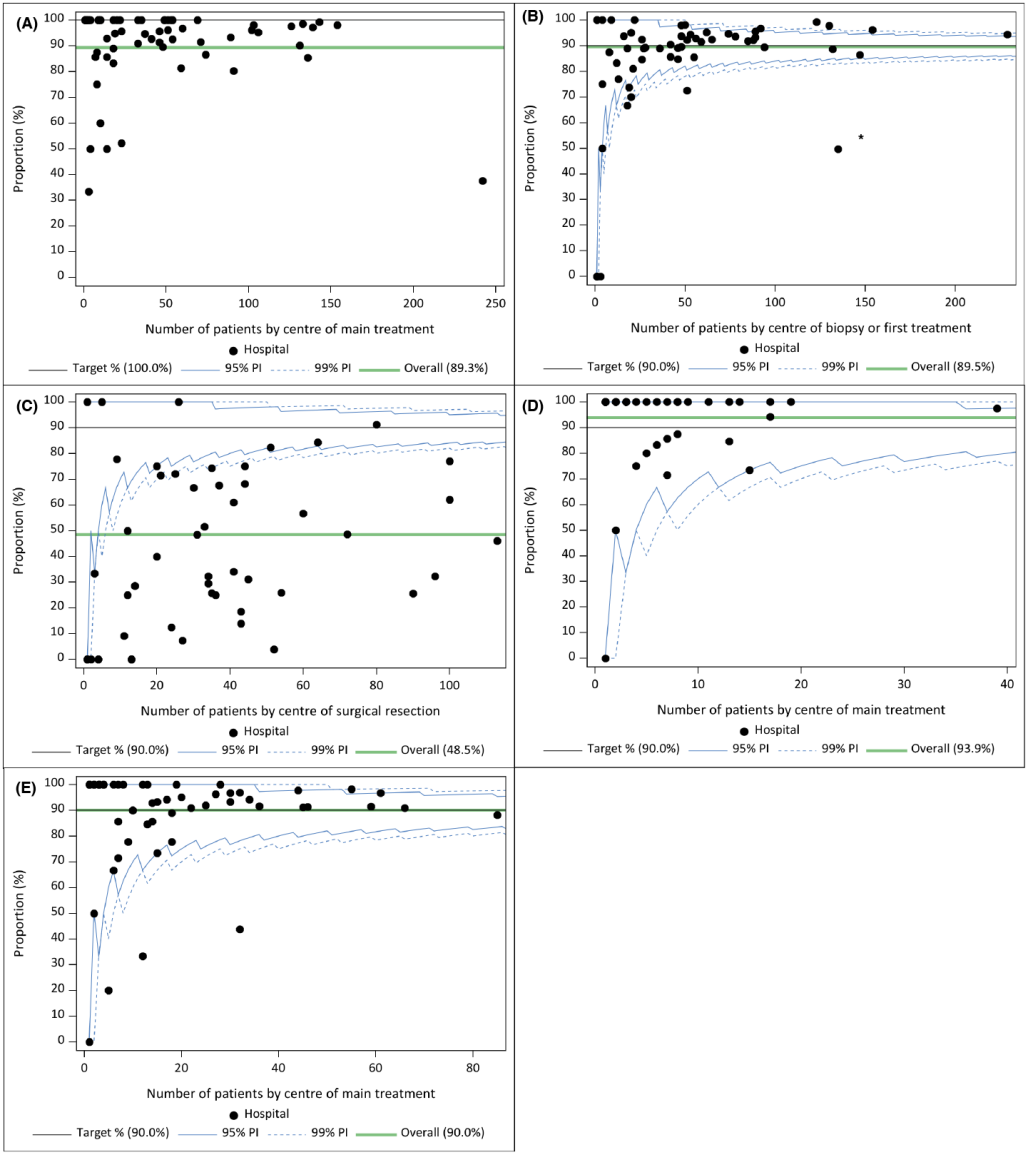
Funnel plots for process indicators on diagnosis and follow‐up imaging. Each dot represents a different hospital. The horizontal axis represents the number of patients, while the vertical axis show the percentage scored for the indicator. By definition, for the same unit size, the precision intervals are broadest when the mean is around 50% and get narrower as the mean approaches more extreme values (0 or 100%). If the precision interval does not cross the reference value, the estimate for that center is statistically significantly different from the reference (at the significance level applied). In a funnel plot, the funnel shape arises due to the expected distribution of the effect size around the predefined target. For a target set at 100%, it is statistically infeasible to calculate meaningful prediction limits. (A) Funnel plot of proportion of glioma patients who have a WHO performance status reported to the BCR, by center of main treatment (73 hospitals reported in plot; 26 hospitals <10 patients in denominator; 8 patients could not be allocated to a center and are thus not represented in the graph). (B) Funnel plot of proportion of glioma patients who underwent MRI (MRI brain or fMRI) before a diagnostic biopsy or before start of treatment in the absence of a diagnostic biopsy, by center of biopsy or first treatment in the absence of a biopsy (58 hospitals reported in plot. 13 hospitals <10 patients in denominator; 2 patients could not be allocated to a center of main treatment and are thus not represented in the graph). * The outlier (indicator result of 49.6% for hospital with 135 patients) appears to be an underestimation of actual practice in the hospital due to recurrent administrative misclassification of MRI. The actual indicator result for that hospital is, based on patient's health records, assumed to be within precision limits. (C) Funnel plot of proportion of patients with high‐grade (grade 3/4) glioma who had postoperative MRI (MRI brain or fMRI) within 3 days after surgical resection, by center of surgery.(53 hospitals reported in funnel plot; 10 hospitals <10 patients in the denominator; and 2 patients could not be allocated to a center of surgery and thus not represented in the graph) (D) Funnel plot of proportion of patients with low‐grade (grade 2) glioma undergoing at least two MRI's (MRI brain or fMRI) in the first year of follow‐up, by center of main treatment (45 hospitals reported in this funnel plot; 36 hospitals <10 patients in the denominator). (E) Funnel plot of proportion of patients with high‐grade (grade 3/4) glioma undergoing at least three MRI's (MRI brain or fMRI) in the first year of follow‐up, by center of main treatment (53 hospitals reported in this funnel plot; 20 hospitals <10 patients in the denominator).

Although the target for this indicator was set at 100%, the population level result for patients diagnosed between 2016 and 2019 was only 89.3%. This was similar (89.0%) in the comparison period 2012–2015, but is a 10% improvement compared to the period 2008–2011 (80.9%). The variability among the different centers is illustrated in Figure 2A. Most hospitals reach a score above 90%, while 6 fall below 60%.

### 3.4 Proportion of glioma patients who are discussed at a multidisciplinary team meeting (MDT)

At the national level, 92.8% of patients were discussed in an MDT within the timeframe of 1 month before to 9 months after the incidence date. Comparison with earlier cohorts shows a gradual improvement over time: 87.7% for 2012–2015 and 75.0% for 2008–2011.

### 3.5 Proportion of glioma patients who underwent MRI before biopsy or before start of treatment

At the national level, during the current study period (2016–2019), 89.5% of glioma patients underwent an MRI scan within the time frame of 6 weeks before until 1 day before diagnostic biopsy or before the commencement of oncological treatment (including surgical resection). These findings were consistent with the results observed in the previous cohorts (2008–2011 and 2012–2015). In 80.6% a diagnostic MRI was performed, while 1.4% had a functional MRI and 7.5% underwent both MRI modalities. As illustrated in Figure 2B, the majority of centers score within random variability around the target of 90%, with only 2 outliers are present.

### 3.6 Proportion of LGG patients in whom PET scan was performed prior to biopsy

At the population level, 32.2% of patients (39 of 121) underwent a PET scan within the time frame of 12 weeks before the biopsy up to the day before. Based on administrative data, identification of the used radiopharmaceutical was not possible in almost half of cases (41.0%).

### 3.7 Proportion of patients with intracranial ependymoma undergoing full spine MR

Only 43.6% (*n* = 17) of patients with an intracranial ependymoma underwent a full spine MRI within the specified time frame of 1 month before to 6 weeks after the incidence date, falling short of the 90% target. Extending the time frame up to 12 weeks after the incidence date increased the proportion to 48.7%. Hospital benchmarking was not performed due to a low number of cases (*n* = 39).

### 3.8 Proportion of high‐grade glioma (HGG) patients undergoing postoperative MRI within 72 h after resection

In the cohort diagnosed during 2016–2019, only 48.5% of HGG underwent an MRI scan within 72 h of surgical resection, while the target was set at 90%. In 38.7% of patients only a CT scan was performed, while in 13.9% both imaging modalities were performed within the first 3 days. We notice a gradual improvement in this indicator in the studied comparison periods: from 30.3% in 2008–2011 to 41.8% in 2012–2015. If the time frame after surgery was extended to 7 days, the proportion increased to 55.4% for the main study cohort, while the proportion for the narrower time frame of 48 h dropped to 39.1%. Figure 2C shows that for the majority of hospitals, this indicator result is below random variability around the target.

### 3.9 Proportion of LGG patients undergoing at least 2 MRIs in the first year of follow‐up

For this indicator, only LGG patients with at least 1 year of follow‐up in the data were taken into account. The result for this indicator at national level is 93.9%, reaching the predefined target of 90%. All hospitals score within random variability around the target of 90%, with 33 hospitals having a maximal score of 100% (Figure 2D). We notice a gradual improvement of this indicator over time in the studied comparison periods: from 89.4% in 2008–2011 to 93.1% in 2012–2015. In the sensitivity analysis assessing the number of patients from the main cohort (2016–2019) in whom at least two MRIs were performed in the second and third follow‐up year, for those who survive the complete second or third year, a decline is noted: 72.5% and 57.4%, respectively.

### 3.10 Proportion of HGG patients undergoing at least 3 MRIs in the first year of follow‐up

For patients with HGG with at least 1 year of follow‐up, in 90.0% at least 3 MRIs were performed (target 90%). As illustrated in Figure 2E, on hospital level the majority of centers score within random variability around the target of 90%. Seventeen hospitals reached the maximum score of 100%, while only three outliers were identified. If compared to earlier cohorts, the result was comparable (92.0%) in 2012–2015, while lower (86.2%) in 2008–2011. In the sensitivity analysis for the main study cohort assessing the second and third years of follow‐up, for those who survive the complete second or third year, the proportion drops respectively to 74.4% and 61.3%.

### 3.11 Proportion of ependymoma patients undergoing at least 3 MRIs in the first year of follow‐up

Only 25 of the 34 ependymoma patients (73.5%) in the cohort diagnosed during 2016–2019 underwent at least 3 follow‐up MRIs in the first year, which is below the predefined target of 90%. However, the proportions rose from 43.8% (2008–2011) to 66.7% (2012–2015) in the historical comparison periods. Hospital benchmarking was not performed because of the low number of cases (*n* = 34).

## 4 DISCUSSION

In this study, for the first time in Belgium, a series of process indicators related to diagnosis and follow‐up imaging in glioma patients has been calculated. The results for the most recent cohort (2016–2019) were at the national level compared with earlier study periods (2008–2011 and 2012–2015), and where possible also hospital‐level results were presented in funnel plots. The number of hospitals involved in the diagnosis and treatment of glioma patients highlights the dispersed of care for glioma patients in Belgium. Since centralization of brain tumor management is currently not mandatory, patient numbers per center remain relatively low. Although the results for most indicators seem satisfying, the results for other indicators are suboptimal.

Since registration of new cancer diagnoses is compulsory in Belgium and WHO performance status is among the required data, a target of 100% was set for the process indicator assessing the availability of the WHO performance score at BCR. However, the result for this cohort is only 89.3% and thus below the predefined target. It is difficult to understand that in about 10% this easily accessible item is left blank on the cancer registration form although it has not been verified whether similar prognosticators as KPS (Karnofsky performance score) were available in the blank cases or whether a WHO score was available in the hospital patient file. For some hospitals failing to provide this WHO score, an important prognostic factor, a clear opportunity for improvement is present. When compared internationally, the result of this indicator is comparable to the reported proportion in Scotland (2020) of 91.7% but inferior to the reported proportion in Sweden (1999–2012) of 97.7%. In both countries there is far more centralization in neuro‐oncological care.^19^, ^20^

Multidisciplinary team discussions (MDTs) have been recognized internationally as an added value in cancer care.^21^ They improve clinical decision‐making and facilitate communication/coordination/continuity of care between healthcare providers. MDTs may also result in better treatment selection, timely treatment, increased recruitment into clinical trials, higher referral to palliative care, and over‐treatment avoidance.^14^ Although the result at national level for the discussion of patients diagnosed with glioma at an MDT meeting seems to provide room for improvement (92.8%), interpretation of the results of this indicator should be done with caution: the proportion of patients discussed at an MDT meeting within the timeframe of 1 month before until 9 months after the incidence date is an underestimation due to billing rules (although patients are often discussed during multiple MDT meetings, only one per patient per diagnosis can be billed per year, sometimes being the one outside the timeframe taken into consideration for the indicator).^14^ Consequently, for this indicator, no target was defined, and no benchmarking between hospitals was performed. In Scotland, where the proportion of patients with brain cancer who are discussed at an MDT meeting prior to any surgical procedure is assessed, the 2020 Clinical Audit Data of the Scottish Adult Neuro Oncology Network (SANON) reports a proportion of 77.1% in 2018, 74.4% in 2019, and 79.6% in 2020. Regional scores in this time frame range from 63.4% to 95.8%.^22^

MRI is the gold standard imaging modality for brain tumors and should precede a diagnostic biopsy or treatment initiation, including surgical resection. Only in an emergency situation or for patients with implanted medical devices MRI can be omitted. For this indicator, the result at national level (89.5%) is in line with the predefined target of 90%. In Scotland, the proportion of patients with brain cancer undergoing MRI in 2020 is 98.3%, while in Denmark it is 99%. This is better than in our cohort. However, it was not specified whether the cohorts in Scotland and Denmark excluded patients with MRI‐incompatible devices.^22^, ^23^ In a national cohort study in England (2013–2014), 93% of patients were considered MRI compatible, while only 80% underwent an MRI before diagnosis.^24^ Across different regions in Sweden, the availability of preoperative MRI for all brain tumors varies between 82% and 96%.^20^

Incorporation of nuclear imaging with amino‐acid PET in defining the target for (stereotactic) biopsy in patients with suspected LGG can reduce the risk of sampling bias and underestimation of the tumor grade.^25^, ^26^, ^27^ However, for this indicator, the multidisciplinary expert board charged with the validation of the TDS judged that, based on absence of clear criteria in the literature, it is impossible to delineate which proportion of LGG patients would benefit from an amino‐acid PET to define a target for biopsy or resection. The calculation of this indicator should be considered as pure descriptive and therefore without target and benchmark. Nevertheless, since PET scan was only performed for 32.2% of patients with a LGG undergoing a biopsy, the performance of amino‐acid PET imaging in glioma should be encouraged and the access to PET imaging, should be facilitated for patients with CNS tumors. Moreover, because in 41% of PET scans, the used tracer could not be identified, hospitals and health authorities should take initiatives to render radiopharmaceuticals administered to patients identifiable in administrative databases.

Given the risk of ependymoma dissemination through the cerebrospinal fluid (CSF), staging of the disease by MRI of the entire neuroaxis is highly recommended. There is no consensus on the timing of spinal MRI with regard to surgery, but it is recommended before the start of adjuvant treatment. Prognosis and treatment are defined based on the grade and the molecular characteristics of the tumor and the stage of the disease.^8^, ^28^, ^29^, ^30^ The low proportion of patients undergoing full spine MRI (43.6%) is far below the 90% target. Moreover, it has to be noted that identification of full spine MRI as such is only reliably possible from December 2018 onward and that an overestimation is most probably made for the preceding period where the billing code for full spine MRI and for an MRI of a segment of spine (cervical, thoracic, or lumbosacral) was the same. Also for follow‐up imaging in ependymoma (73.5% vs. target 90%), a clear opportunity for improvement is identified. For the indicators assessing imaging in ependymoma (i.e., full spine diagnostic MRI and follow‐up MRI in the first year after biopsy or treatment), international comparison is not possible since no data are available.

Assessing the extent of resection is essential, not only from a prognostic point of view or to have a baseline for further follow‐up, but also to identify a possible, albeit rare indication for a second‐look surgery. For the indicator assessing postoperative imaging within 72 h, the result (48.5%) is notably below the predefined target of 90%. This outcome aligns with the UK (45%), but trails Denmark (89%) and Sweden (90%).^5^, ^20^, ^24^ It is important to point out that the result in Denmark increased substantially since the onset of scoring this indicator in 2010 (from 63% in 2010 to 89% in 2014). In this cohort, an immediate (within 3 days) postoperative CT scan was performed as the sole imaging modality in 38.7% of HGG patients, while both CT and MRI were performed in 13.9%. Although CT scan imaging can be indicated in the postoperative course of glioma surgery to identify (hemorrhagic) complications in emergency settings, it is not the imaging modality of choice for routine postoperative evaluation. Neurosurgeons, together with radiologists, should organize their practice in order to pursue timely (within 72 h) MRI for all high‐grade glioma patients rather than CT scans in the postoperative setting, unless in emergency situations.

To monitor the evolution of the disease after treatment or biopsy and to detect potential evolution towards HGG, sequential imaging with MRI is considered good practice. For the indicators assessing follow‐up imaging in LGG (2/year) and HGG (3/year), at the national level, the predefined target is reached in the first year of follow‐up: 93.9% and 90.0%, respectively. A remarkable decline of the proportion is noted for the second follow‐up year (71.4% for LGG and 74.6% for HGG). For LGG, this could be explained by the fact that for patients with complete removal of the tumor, the follow‐up can be already loosened after 1 year. For HGG patients alive at 2 years follow‐up, follow‐up with imaging could have been halted due noted disease progression, making repetitive MRI burdensome and less meaningful. Also, for these indicators, international data for comparison are not available.

For all process indicators assessing imaging in the postoperative course, the use of different MRI sequences, the administration of contrast agents and a volumetric assessment of the residual tumor, as demanded in the original formulation, cannot be evaluated based on the administrative code of MRI brain.

A first major strength of this analysis is the fact that for the first time process indicators related to the diagnosis of glioma are calculated for Belgium. In this way, this work can induce awareness for quality of care for these patients and eventual improvement in care processes. A second important strength is that the study cohort was selected from the BCR database, a national population‐based cancer registry with high completeness, and therefore includes nearly all adult glioma cases fulfilling the inclusion criteria. In this way, the care patterns across all Belgian hospitals treating glioma patients could be described and benchmarked.

This study also incorporates some limitations. Most important, process indicators on population level are calculated by using administrative data. First, this implies that adaptations to and simplifications of the original formulation of the indicators were needed. Second, the clinical intention behind a registered administrative code is unknown; therefore, timeframes have to be set, which might inadvertently lead to incorrect inclusion or exclusion of diagnostic or therapeutic procedures. Thirdly, the interpretation of administrative data is not always straightforward (for instance, the same billing code referring to both a full spine MRI and an MRI of a segment of the spine until December 2018 or the limitation of reimbursement of only one MDT discussion per calendar year). Fourth, important clinical events such as recurrence/progression or participation in clinical trials, that could provide an explication for an alternation in patients' care paths, cannot be identified in administrative reimbursement databases. Next, in a ‘Fee for Service’ healthcare system some variation in the use of billing codes or misclassifications could be present, disabling the identification of an exact diagnostic and therapeutic pathway. Also, data can be missing (even obligatory cancer registration data such as WHO performance score) or, although rare, be incorrect or incomplete, as previously illustrated with regard to the ICD‐O‐3 topography and morphology codes upon cancer registration.^10^ Another limitation is the retrospective nature of the calculation. The main study cohort in this publication dates to 2016–2019. Results at national level and feedback reports to hospitals were available in Q1 of 2024, due to the inherent delay for population databases to reach completeness and the considerable time necessary for data preparation, calculation, and validation of the results. In this way, measures to be taken by hospitals based on the received feedback report can only be implemented from the start of 2024 onwards and thus will only become beneficial and evaluable in the years to come. By that time, scientific evidence and thus the rationale for certain indicators might have changed. On the other hand, now that the methodology has been developed in detail and since administrative data are generated and stored continuously, repeating this assessment to evaluate the extent of improvement will be possible in a faster and more efficient way.

## 5 CONCLUSION

This pioneering study on neuro‐oncological care in Belgium offers a comprehensive assessment of diagnostic care and follow‐up imaging practices in glioma patients using a set of carefully designed process indicators. This analysis provides valuable insights into the care paths of glioma patients across Belgian hospitals. Benchmarking against previous cohorts and with international results where possible, allow a nuanced evaluation.

Both exemplary practices and areas for improvement in neuro‐oncological care are revealed. Notable achievements such as the performance of MDT discussions and the gradual improvement in MRI utilization at diagnosis and for follow‐up in LGG, and HGG are described. On the other hand, also some challenges are identified, such as the incomplete reporting of the WHO performance status, the need for more PET imaging in LGG and the suboptimal rates of both full spine MRI around diagnosis and follow‐up MRI after resection in ependymoma patients. Especially variations in early postoperative imaging practices in HGG are detected, creating a substantial opportunity for improvement for a lot of centers. Individual feedback reports on the assessment of these process indicators were provided to all Belgian hospitals, enabling reflection among the physicians involved in neuro‐oncology care programs and the hospital management.

As mentioned earlier, this first calculation of process indicators serves as a crucial starting point and should not be used to criticize individual hospitals. Contrarily, it creates opportunities for quality improvement or could act as a motivator to continue good clinical practices for those already performing well. It is clear from these data that steps for further improvement will require a joint effort from physicians involved in neuro‐oncology care programs, hospitals, and health care administrators and policymakers in Belgium. Monitoring these process indicators in the future will be an important instrument to support this effort. Moreover, the authors anticipate that the methodology established in this study could also initiate or intensify quality of care enhancements in neuro‐oncology internationally.

## AUTHOR CONTRIBUTIONS

**Dimitri Vanhauwaert:** Conceptualization (equal); data curation (equal); formal analysis (equal); methodology (equal); project administration (equal); visualization (equal); writing – original draft (lead); writing – review and editing (supporting). **Katrijn Vanschoenbeek:** Data curation (lead); formal analysis (equal); methodology (equal); project administration (equal); visualization (lead); writing – review and editing (lead). **Frank Weyns:** Validation (supporting); writing – review and editing (supporting). **Ludo Vanopdenbosch:** Validation (supporting); writing – review and editing (supporting). **Ann Tieleman:** Validation (supporting); writing – review and editing (supporting). **Alex Michotte:** Validation (supporting); writing – review and editing (supporting). **Karolien Goffin:** Validation (supporting); writing – review and editing (supporting). **Cindy De Gendt:** Methodology (lead); project administration (equal); supervision (lead); validation (lead); writing – review and editing (lead). **Steven De Vleeschouwer:** Supervision (lead); validation (lead); writing – review and editing (lead). **Tom Boterberg:** Supervision (lead); validation (lead); writing – review and editing (lead).

## FUNDING INFORMATION

This research was financially supported by *Stichting tegen Kanker* and *AZ Delta vzw*. We would also like to acknowledge the *Ghent University Arne Lannoy, A.K.A. Zorro Fund* for contributing to the publication fee.

## CONFLICT OF INTEREST STATEMENT

SDV is a certified Gliolan (Medac GmbH) Trainer and has performed consultancy for Lamepro NV (current Pharmanovia) (Gliolan) for which a fee was received. All other authors have no relevant financial or non‐financial interests to disclose.

## ETHICS STATEMENT

This study was approved by the Ethics Committee of Ghent University Hospital (DA 2020‐042). Written informed consent from participants was not required in accordance with local/national guidelines.

## Supporting information


Appendix S1.

Appendix S2.

Appendix S3.

## Data Availability

The cancer cohort data used and analyzed during the study are available via the Belgian Cancer Registry (BCR) upon reasonable request. The pseudonymized data can be provided within the secured environment of the BCR after having been guaranteed that the applicable GDPR regulations are applied.
