# Supplementary material for: Measuring the diagnostic management and follow‐up imaging for glioma patients across Belgian hospitals between 2016 and 2019
Source: Cancer Med. 2024 Oct 30;13(21):e70045. doi: 10.1002/cam4.70045 (PMC11523142; doi:10.1002/cam4.70045)
Supplement: Supplementary file 1 — Appendix S1. [file CAM4-13-e70045-s001.docx]

# appendix 1: technical documentation sheets Quality of DIAGNOSTICS IN GLIOMA

## Documented WHO performance status (D01)

| Title | Proportion of glioma patients who have a documented WHO performance status or Karnofsky performance score at time of multidisciplinary team discussion |
| --- | --- |
| Rationale | The WHO performance status (or Karnofsky performance score) is an important prognostic factor for clinical outcomes of glioma patients. Judging performance status is the key to determine optimal treatment for each individual patient (1-10). Furthermore, complete data on performance status can be used for case-mix correction, e.g. to investigate the effect of treatment in a population. |
| Type of indicator | Process indicator with benchmarking |
| Reformulation | Proportion of glioma patients who have a WHO performance status reported to the Belgian Cancer Registry (BCR) |
| Calculation | Numerator: Glioma patients who have a WHO performance status reported to the BCR  Denominator: All glioma patients |
| Target | 100%  Since the registration of the WHO performance status is mandatory in cancer registration, the target is set at 100% (11). |
| Data source | Belgian Cancer Registry (BCR): incidence years 2016-2019 2 (main study period) and incidence years 2012-2015 and 2008-2011 (comparison periods for sensitivity analysis)  IMA data: 2015-2021(Q2) |
| Technical definition | - Diagnosis of glioma: ICD-O-3: 938-945 (C71) - Incidence date as registered at BCR (= date of first microscopic confirmation of malignancy, if not available, the incidence date is determined in decreasing priority by 1) first hospitalization for cancer, 2) first consultation because of malignancy, 3) initial clinical or technical diagnosis, 4) start of treatment for cancer and 5) death) - Diagnostic biopsy - Timeframe: from 3 months before until 3 months after incidence date (and before surgical resection in case surgical resection was performed): nomenclature codes (IMA, table 1) - Oncological treatment - Surgical resection (timeframe: from 1 month before until 9 months after incidence date. Note: in case a biopsy was followed by chemo- and/or radiotherapy and subsequently by a surgical resection, the surgical resection was not withheld as primary treatment): nomenclature codes (IMA, table 2) - Chemotherapy (timeframe in the presence of surgical resection from day of surgical resection until 12 months after surgical resection (adjuvant); timeframe in the absence of surgical resection: from 1 month before until 9 months after incidence date): ATC codes (IMA, table 3) - Radiotherapy (timeframe in the presence of surgical resection: from day of surgical resection until 12 months after surgical resection (adjuvant); timeframe in the absence of surgical resection: from 1 month before until 9 months after incidence date): nomenclature codes (IMA, table 4) - Multiple tumours:   If additional tumours around the diagnosis of the glioma (i.e. from 5 years before to maximum 2 years after the incidence date of the glioma) are present (Note: only information about tumours with an incidence <= the year 2021 is available)   - Inside the CNS, benign, borderline or malignant tumours are taken into account - Outside the CNS, only malignant tumours are considered - Comorbidities (diabetes, cardiovascular disease and respiratory disease) - Calculation is based on medication use in the year prior to the glioma diagnosis - Per comorbidity, a set of ATC codes is withheld (IMA, table 5). If the annual use of this set of ATC codes by a patient exceeds a certain threshold, this comorbidity is considered to be present |
| Risk adjustment | None (process indicator) |
| Limitations | The original indicator mentions Karnofsky performance score or WHO performance status to classify the clinical status of patients at time of multidisciplinary team (MDT) discussion. The Karnofsky performance score (10 categories), which is more sensitive to assess clinical performance than the WHO performance status (only 5 categories), is not available in the BCR data. Only the WHO performance status reported to the BCR is available for this study. This corresponds to the performance status at time of diagnosis, which can be different from the WHO performance score at the moment of the MDT. For a more detailed neurological status of patients with glioma, assessment of the NANO-score could be considered. However, also this score is not available in the BCR data. |
| Subgroup analyses | - Year of incidence - Age at time of diagnosis - Sex - Multiple tumours - Comorbidities (diabetes, cardiovascular disease, respiratory disease) - Treatment scheme |
| Sensitivity analyses | - Incidence year 2008-2011 vs 2012-2015 vs 2016-2019 |
| Benchmarking | Centre of main treatment |
| International results | See Table 1.1 – International results |

Table 1.1 – International results

| Author | Period covered | Country | Results |
| --- | --- | --- | --- |
| Liaquat (12) | 2018-2020 | Scotland | In the 2022 report of the Scottish Neuro Oncology Network (SANON) the proportion of patients with brain cancer who have a **documented WHO status at time of multidisciplinary team discussion** for Scotland (population based) is **91.1% in 2019** and **91.7% in 2020**. |
| Liaquat (13) | 2018 | Scotland | In the report of the 2018 Clinical Audit Data of the Scottish Adult Neuro Oncology Network (SANON), the proportion of patients with brain cancer who have a **documented WHO status at time of multidisciplinary team discussion** is **92.0%** for Scotland (population based). Regional scores range from 90.0% to 94.1%. |
| Asklund (14) | 1999-2012 | Sweden | In Sweden reporting of every newly detected cancer is compulsory and WHO performance status is among the required data. The time point for which the WHO performance score is required is not specified. According to the data provided in the supplementary material to the article, **the reporting rate of the WHO status is 97.7%** |

References

1. Scottish Cancer Taskforce. Brain and Central Nervous System Cancer Clinical Quality Performance Indicators 2018 [Available from: https://consult.gov.scot/nhs/brain-cns-cancer-qpis/consult_view/.

2. Weller M, van den Bent M, Hopkins K, Tonn JC, Stupp R, Falini A, et al. EANO guideline for the diagnosis and treatment of anaplastic gliomas and glioblastoma. Lancet Oncol. 2014;15(9):e395-403.

3. Landelijke werkgroep neuro-oncologie. Kwaliteitscriteria neuro-oncologie. Diagnostiek, behandeling en begeleiding van patiënten met een glioom. 2014 [Available from: https://richtlijnendatabase.nl/uploaded/docs/IKNL_in_ontw/Kwaliteitscriteria_Gliomen_def_mei2014.pdf.

4. Weller M, Deutsche Gesellschaft für Neurologie. Leitlinien für Diagnostik und Therapie in der Neurologie 2015 [Available from: https://www.awmf.org/uploads/tx_szleitlinien/030-099l_S2k_Gliome_2015-06-abgelaufen.pdf.

5. Anocef. Référentiel Glioblastome ( grade IV OMS) [Available from: http://oncologik.fr/referentiels/anocef.

6. Anocef. Recommandations Anocef 2018 Traitement des Gliomes malins Grade III et IV OMS 2018 [Available from: https://www.anocef.org/index.php?pageID=sommaire_accueil.

7. Watts C. Surgical management of high-grade glioma: a standard of care. CNS oncology. 2012;1(2):181-92.

8. Vanhauwaert D, Pinson H, Sweldens C, Du Four S, Van Eycken L, De Schutter H, et al. Quality indicators in neuro-oncology: review of the literature and development of a new quality indicator set for glioma care through a two-round Delphi survey. Journal of neuro-oncology. 2022.

9. Oken MM, Creech RH, Tormey DC, Horton J, Davis TE, McFadden ET, et al. Toxicity and response criteria of the Eastern Cooperative Oncology Group. Am J Clin Oncol. 1982;5(6):649-55.

10. Karnofsky D, Burchenal J. The clinical evaluation of chemotherapeutic agents in cancer. In: MacLeod C, editor. Evaluation of chemotherapeutic agents. New York: Columbia University Press; 1949. p. 191–205.

11. Scottish Cancer Taskforce. Brain and Central Nervous System Cancer Clinical Quality Performance Indicatores V2021 2021 [Available from: https://www.healthcareimprovementscotland.org/our_work/cancer_care_improvement/cancer_qpis/quality_performance_indicators.aspx.

12. Liaquat I, Calmpbell L, New D. Brain and Central Nervous System Cancers Quality Performance Indicators. Report of the 2018 Clinical Audit Data. 2019 [

13. Liaquat I, Campbell L, McMahon J. Audit Report: Brain and Central Nervous System Cancers Quality Performance Indicators. Report of the 2020 Clinical Audit Data. Scottish Adult Neuro Oncology Network,; 2022. p. 41.

14. Asklund T, Malmstrom A, Bergqvist M, Bjor O, Henriksson R. Brain tumours in Sweden: data from a population-based registry 1999-2012. Acta Oncol. 2015;54(3):377-84.

## MULTIDISCIPLINARY TEAM MEETING PRIOR TO DEFINITIVE MANAGEMENT (X01)

| Title | Proportion of glioma patients who are discussed at a multidisciplinary team (MDT) meeting after obtaining pathological diagnosis prior to definitive management (chemotherapy, radiotherapy, resection after initial biopsy or second look surgery) |
| --- | --- |
| Rationale | A multidisciplinary team (MDT) meeting to assess the optimal treatment strategy for patients with glioma is highly recommended. The most reasonable moment for this MDT is after a pathological diagnosis is obtained and before definitive management is started. Not only chemo and/or radiotherapy are considered as definitive management but also resection after initial biopsy and second look surgery. Also when (further) treatment is not indicated, discussion at an MDT is mandatory (1-15). |
| Type of indicator | Process indicator without benchmarking (‘descriptive indicator’) |
| Reformulation | Proportion of glioma patients who are discussed at a multidisciplinary team (MDT) meeting between 1 month before incidence date and 9 months after incidence date |
| Calculation | Numerator: Glioma patients who were discussed at an MDT meeting between 1 month before incidence date and 9 months after incidence date  Denominator: All glioma patients |
| Target | No target.  (Given the limitations regarding the administrative data on MDT and its billing rules, this process indicator is considered as purely descriptive (without calculation of the national average, target and benchmarking).) |
| Data source | Belgian Cancer Registry (BCR): incidence years 2016-2019  IMA data: 2015-2021(Q2) |
| Technical definition | - Diagnosis of glioma: ICD-O-3: 938-945 (C71) - Incidence date as registered at BCR (= date of first microscopic confirmation of malignancy, if not available, the incidence date is determined in decreasing priority by 1) first hospitalization for cancer, 2) first consultation because of malignancy, 3) initial clinical or technical diagnosis, 4) start of treatment for cancer and 5) death) - Diagnostic biopsy - Timeframe: from 3 months before until 3 months after incidence date (and before surgical resection in case surgical resection was performed): nomenclature codes (IMA, table 1) - Oncological treatment - Surgical resection (timeframe: from 1 month before until 9 months after incidence date. Note in case a biopsy was followed by chemo- and/or radiotherapy and subsequently by a surgical resection, the surgical resection was not withheld as primary treatment): nomenclature codes (IMA, table 2) - Chemotherapy (timeframe in the presence of surgical resection: from day of surgical resection until 12 months after surgical resection (adjuvant); timeframe in the absence of surgical resection: from 1 month before until 9 months after incidence date): ATC codes (IMA, table 3) - Radiotherapy (timeframe in the presence of surgical resection: from day of surgical resection until 12 months after surgical resection (adjuvant); timeframe in the absence of surgical resection: from 1 month before until 9 months after incidence date): nomenclature codes (IMA, table 4) - Multiple tumours:   If additional tumours around the diagnosis of the glioma (i.e. from 5 years before to maximum 2 years after the incidence date of the glioma) are present (Note: only information about tumours with an incidence <= the year 2021 is available)   - Inside the CNS, benign, borderline or malignant tumours are taken into account - Outside the CNS, only malignant tumours are considered   - Comorbidities (diabetes, cardiovascular disease and respiratory disease)   - Calculation is based on medication use in the year prior to the glioma diagnosis - Per comorbidity, a set of ATC codes is withheld (IMA, table 5). If the annual use of this set of ATC codes by a patient exceeds a certain threshold, this comorbidity is considered to be present   - MDT meeting: nomenclature codes (IMA, table 6) |
| Risk adjustment | None (process indicator) |
| Limitations | Based on the administrative data, only the MDT meetings that have been charged can be identified. Due to the reimbursement rules, which imply that only one MDT meeting can be billed per patient and per calendar year, these data may underestimate the real frequency of MDT meetings (16). For that reason, it is possible that an MDT meeting after biopsy or surgical resection is not identifiable, because an earlier MDT meeting (e.g. after imaging and before first surgical intervention) was held and billed. The time frame in which an MDT meeting is captured is defined around the incidence date and not around date of pathological confirmation. Another limitation is that we do not have any information on the quality of the MDT meeting itself, nor on the multidisciplinary approach during the entire care pathway. |
| Subgroup analyses | - Year of incidence - Age at time of diagnosis - Sex - Multiple tumours - Glioma subtype - Comorbidities (diabetes, cardiovascular disease, respiratory disease) - Treatment scheme - Basis of diagnosis |
| Sensitivity analyses | - Incidence year 2008-2011 vs 2012-2015 vs 2016-2019 - MDT meeting within different timeframes around incidence date - MDT meeting within 1 month before incidence date until the day before start of first treatment |
| Benchmarking | / ( descriptive indicator) |
| International results | See Table 2.1 – International results |

Table 2.1 – International results

| Author | Period covered | Country | Results |
| --- | --- | --- | --- |
| Liaquat (17) | 2018-2020 | Scotland | In the report of the 2020 Clinical Audit Data of the Scottish Adult Neuro Oncology Network (SANON), the **proportion of patients with brain cancer who are discussed at a multidisciplinary meeting (MDT) prior to any surgical procedure** for Scotland (population based) is **77.1% in 2018, 74.4% in 2019 and 79.6% in 2020**. Regional scores in this time frame range from 95.8% to 63.4%. |

References

1. Riblet NB, Schlosser EM, Homa K, Snide JA, Jarvis LA, Simmons NE, et al. Improving the quality of care for patients diagnosed with glioma during the perioperative period. J Oncol Pract. 2014;10(6):365-70.

2. Jordan JT, Sanders AE, Armstrong T, Asher T, Bennett A, Dunbar E, et al. Quality improvement in neurology: Neuro-Oncology Quality Measurement Set. Neuro Oncol. 2018;20(4):531-7.

3. Wright FC, De Vito C, Langer B, Hunter A, Expert Panel on Multidisciplinary Cancer Conference S. Multidisciplinary cancer conferences: a systematic review and development of practice standards. Eur J Cancer. 2007;43(6):1002-10.

4. Lutterbach J, Pagenstecher A, Spreer J, Hetzel A, Velthoven V, Nikkhah G, et al. The brain tumour board: lessons to be learned from an interdisciplinary conference. Onkologie. 2005;28(1):22-6.

5. Integraal Kankercentrum Nederland. Gliomen, landelijke richtlijn, versie:3.0 2015 [Available from: https://www.oncoline.nl/gliomen.

6. Landelijke werkgroep neuro-oncologie. Kwaliteitscriteria neuro-oncologie. Diagnostiek, behandeling en begeleiding van patiënten met een glioom. 2014 [Available from: https://richtlijnendatabase.nl/uploaded/docs/IKNL_in_ontw/Kwaliteitscriteria_Gliomen_def_mei2014.pdf.

7. Weller M, Deutsche Gesellschaft für Neurologie. Leitlinien für Diagnostik und Therapie in der Neurologie 2015 [Available from: https://www.awmf.org/uploads/tx_szleitlinien/030-099l_S2k_Gliome_2015-06-abgelaufen.pdf.

8. Corso CD, Bindra RS, Mehta MP. The role of radiation in treating glioblastoma: here to stay. Journal of neuro-oncology. 2017;134(3):479-85.

9. Berrocal A, Gil M, Gallego Ó, Balaña C, Pérez Segura P, García-Mata J, et al. SEOM guideline for the treatment of malignant glioma. Clin Transl Oncol. 2012;14(7):545-50.

10. Kim YZ, Kim CY, Lim J, Sung KS, Lee J, Oh HJ, et al. The Korean Society for Neuro-Oncology (KSNO) Guideline for WHO Grade III Cerebral Gliomas in Adults: Version 2019.01. Brain Tumour Res Treat. 2019;7(2):63-73.

11. Kim YZ, Kim CY, Lim J, Sung KS, Lee J, Oh HJ, et al. The Korean Society for Neuro-Oncology (KSNO) Guideline for Glioblastomas: Version 2018.01. Brain Tumour Res Treat. 2019;7(1):1-9.

12. Belgian College of Oncology. National Clinical Practice Guidelines Neuro-Oncology version 1.2008. 2008.

13. Vanhauwaert D, Pinson H, Sweldens C, Du Four S, Van Eycken L, De Schutter H, et al. Quality indicators in neuro-oncology: review of the literature and development of a new quality indicator set for glioma care through a two-round Delphi survey. Journal of neuro-oncology. 2022.

14. Khalafallah AM, Jimenez AE, Romo CG, Kamson DO, Kleinberg L, Weingart J, et al. Quantifying the utility of a multidisciplinary neuro-oncology tumour board. J Neurosurg. 2020:1-6.

15. Snyder J, Schultz L, Walbert T. The role of tumour board conferences in neuro-oncology: a nationwide provider survey. J Neurooncol. 2017;133(1):1-7.

16. Vrijens F, Kohn L, Dubois C, Leroy R, I V, Stordeur S. Ten years of multidisciplinary team meetings in oncology: current situation and perspectives. In: (KCE) BHCKC, editor. KCE reports 239C2015.

17. Liaquat I, Campbell L, McMahon J. Audit Report: Brain and Central Nervous System Cancers Quality Performance Indicators. Report of the 2020 Clinical Audit Data. Scottish Adult Neuro Oncology Network,; 2022. p. 41.

## DIAGNOSTIC MRI (D02)

| Title | Proportion of patients with suspected brain tumour who underwent MRI with T2, T1 and T1 contrast enhanced weighted images |
| --- | --- |
| Rationale | MRI is the gold standard and the modality of choice for imaging in brain tumours and all patients with central nervous system tumours should receive an MRI prior to both diagnostic biopsy and oncological treatment (1-15). The minimum MRI exam should include T1 weighted images pre- and post-gadolinium contrast and T2 weighted images (16-21). |
| Type of indicator | Process indicator with benchmarking |
| Reformulation | Proportion of glioma patients who underwent MRI (MRI brain or fMRI) before a diagnostic biopsy or before start of treatment in the absence of a diagnostic biopsy |
| Calculation | Numerator: Glioma patients who underwent MRI (MRI brain or fMRI) from 6 weeks before until 1 day before diagnostic biopsy or before start of oncological treatment in the absence of a diagnostic biopsy  Denominator: Glioma patients who received a diagnostic biopsy and/or oncological treatment (surgical resection, chemotherapy and/or (chemo)radiotherapy) |
| Target | 90%  Tolerance considers:   - Surgical interventions in an emergency setting - Patients with implanted devices with contra-indications for MRI |
| Data source | Belgian Cancer Registry (BCR): incidence years 2016-2019 (main study period) and incidence years 2012-2015 and 2008-2011 (comparison periods for sensitivity analysis)  IMA data: 2015-2021(Q2) |
| Technical definition | - Diagnosis of glioma: ICD-O-3: 938-945 (C71) - Incidence date as registered at BCR (= date of first microscopic confirmation of malignancy, if not available, the incidence date is determined in decreasing priority by 1) first hospitalization for cancer, 2) first consultation because of malignancy, 3) initial clinical or technical diagnosis, 4) start of treatment for cancer and 5) death) - Diagnostic biopsy - Timeframe: from 3 months before until 3 months after incidence date (and before surgical resection in case surgical resection was performed): nomenclature codes (IMA, table 1) - Oncological treatment - Surgical resection (timeframe: from 1 month before until 9 months after incidence date. Note: in case a biopsy was followed by chemo- and/or radiotherapy and subsequently by a surgical resection, the surgical resection was not withheld as primary treatment): nomenclature codes (IMA, table 2) - Chemotherapy (timeframe in the presence of surgical resection: from day of surgical resection until 12 months after surgical resection (adjuvant); timeframe in the absence of surgical resection: from 1 month before until 9 months after incidence date): ATC codes (IMA, table 3) - Radiotherapy (timeframe in the presence of surgical resection: from day of surgical resection until 12 months after surgical resection (adjuvant); timeframe in the absence of surgical resection: from 1 month before until 9 months after incidence date): nomenclature codes (IMA, table 4) - Multiple tumours   If additional tumours around the diagnosis of the glioma (i.e. from 5 years before to maximum 2 years after the incidence date of the glioma) are present (Note: only information about tumours with an incidence <= the year 2021 is available)   - Inside the CNS, benign, borderline or malignant tumours are taken into account - Outside the CNS, only malignant tumours are considered   - Comorbidities (diabetes, cardiovascular disease and respiratory disease)   - Calculation is based on medication use in the year prior to the glioma diagnosis - Per comorbidity, a set of ATC codes is withheld (IMA, table 5). If the annual use of this set of ATC codes by a patient exceeds a certain threshold, this comorbidity is considered to be present - Diagnostic MRI (MRI brain or fMRI) - Timeframe: from 6 weeks before until the day before diagnostic biopsy or before start of treatment in the absence of a diagnostic biopsy: nomenclature codes (IMA, table 7) - Contrast administration: ATC code (IMA, table 8) - Alternative diagnostic imaging - Timeframe PET: from 6 weeks before until the day before diagnostic biopsy or before start of treatment in the absence of a diagnostic biopsy: nomenclature codes (IMA, table 9) - Timeframe CT: from 6 weeks before until the day before diagnostic biopsy or before start of treatment in the absence of a diagnostic biopsy: nomenclature codes (IMA, table 11) |
| Risk adjustment | None (process indicator) |
| Limitations | Only patients with confirmed brain tumours (through histopathology or imaging) are registered and are amenable for assessment of indicators. Therefore, the term “suspected” is omitted in the formulation of the indicator.  Different MRI (such as T1 and T2) sequences cannot be defined from the administrative databases. |
| Subgroup analyses | - Year of incidence - Age at time of diagnosis - Sex - Multiple tumours - Glioma subtype - WHO performance status - Comorbidities (diabetes, cardiovascular disease, respiratory disease) - Treatment scheme |
| Sensitivity analyses | - Incidence year 2008-2011 vs 2012-2015 vs 2016-2019 - Contrast administration (only MRI with contrast versus only MRI without contrast versus both MRI with and without contrast) - Type of MRI (only MRI brain versus only fMRI versus both MRI brain and fMRI) - MRI within different timeframes before diagnostic biopsy or before start of oncological treatment in the absence of a diagnostic biopsy - Type of imaging (MRI, PET and/or CT) before diagnostic biopsy or before start of oncological treatment in the absence of a diagnostic biopsy) |
| Benchmarking | Centre of biopsy or first treatment (in the absence of a biopsy) |
| International results | See Table 3.1 – International results |

Table 3.1 – International results

| Author | Period covered | Country | Results |
| --- | --- | --- | --- |
| Liaquat (22) | 2018 | Scotland | In the report of the 2020 Clinical Audit Data of the Scottish Adult Neuro Oncology Network (SANON), the **proportion of patients with brain cancer undergoing surgery who have contrast enhanced MRI prior to treatment** is **98.3%**. |
| Dumba (23) | 2013-2014 | England | In this national cohort study on imaging in glioblastoma patients, 93% of patients were considered MRI compatible (i.e. received at least 1 MRI in the dataset). **80%** **of all patients received MRI before diagnosis**. |
| Rasmussen (24) | 2009-2014 | Denmark | The Danish Neuro-Oncology Registry reports **availability of a preoperative MRI in 1 911 (99%) of 1 930 of glioma patients and data on contrast administration in 1 873 (97%)**. However, it is not specified whether the cohort consisted of MRI compatible patients. |
| Asklund (25) | 1999-2012 | Sweden | In Sweden reporting of every newly detected cancer is compulsory. Preoperative symptoms, pre- and postoperative neuroradiological investigations, tumour size and location, and extent of surgery are registered in the Swedish Brain Tumour Registry (SBTR). **In the study period 2004-2007 availability of preoperative MRI for all brain tumours** (i.e. not only gliomas) **ranges between 44% and 87%** depending on the region. **For the 2008-2012 cohort the availability ranges between 82% and 96%**. |

References

1. National Institute for Health and Care Excellence (NICE). Brain tumours (primary) an brain metastases in adults. 2018 [Available from: https://www.nice.org.uk/guidance/ng99.

2. Landelijke werkgroep neuro-oncologie. Kwaliteitscriteria neuro-oncologie. Diagnostiek, behandeling en begeleiding van patiënten met een glioom. 2014 [Available from: https://richtlijnendatabase.nl/uploaded/docs/IKNL_in_ontw/Kwaliteitscriteria_Gliomen_def_mei2014.pdf.

3. Weller M, van den Bent M, Hopkins K, Tonn JC, Stupp R, Falini A, et al. EANO guideline for the diagnosis and treatment of anaplastic gliomas and glioblastoma. Lancet Oncol. 2014;15(9):e395-403.

4. Weller M, Deutsche Gesellschaft für Neurologie. Leitlinien für Diagnostik und Therapie in der Neurologie 2015 [Available from: https://www.awmf.org/uploads/tx_szleitlinien/030-099l_S2k_Gliome_2015-06-abgelaufen.pdf.

5. Berrocal A, Gil M, Gallego Ó, Balaña C, Pérez Segura P, García-Mata J, et al. SEOM guideline for the treatment of malignant glioma. Clin Transl Oncol. 2012;14(7):545-50.

6. Mason WP, Maestro RD, Eisenstat D, Forsyth P, Fulton D, Laperrière N, et al. Canadian recommendations for the treatment of glioblastoma multiforme. Curr Oncol. 2007;14(3):110-7.

7. Essig M, Anzalone N, Combs SE, Dörfler À, Lee SK, Picozzi P, et al. MR imaging of neoplastic central nervous system lesions: review and recommendations for current practice. AJNR Am J Neuroradiol. 2012;33(5):803-17.

8. Jiang T, Mao Y, Ma W, Mao Q, You Y, Yang X, et al. CGCG clinical practice guidelines for the management of adult diffuse gliomas. Cancer Lett. 2016;375(2):263-73.

9. Mukundan S, Holder C, Olson JJ. Neuroradiological assessment of newly diagnosed glioblastoma. Journal of neuro-oncology. 2008;89(3):259-69.

10. Kim YZ, Kim CY, Lim J, Sung KS, Lee J, Oh HJ, et al. The Korean Society for Neuro-Oncology (KSNO) Guideline for WHO Grade III Cerebral Gliomas in Adults: Version 2019.01. Brain Tumour Res Treat. 2019;7(2):63-73.

11. Kim YZ, Kim CY, Lim J, Sung KS, Lee J, Oh HJ, et al. The Korean Society for Neuro-Oncology (KSNO) Guideline for Glioblastomas: Version 2018.01. Brain Tumour Res Treat. 2019;7(1):1-9.

12. Stupp R, Pavlidis N, Jelic S, Force EGT. ESMO Minimum Clinical Recommendations for diagnosis, treatment and follow-up of malignant glioma. Annals of oncology : official journal of the European Society for Medical Oncology. 2005;16:i64-i5.

13. Sepúlveda-Sánchez JM, Muñoz Langa J, Arráez MÁ, Fuster J, Hernández Laín A, Reynés G, et al. SEOM clinical guideline of diagnosis and management of low-grade glioma (2017). Clin Transl Oncol. 2018;20(1):3-15.

14. Scottish Cancer Taskforce. Brain and Central Nervous System Cancer Clinical Quality Performance Indicators 2018 [Available from: https://consult.gov.scot/nhs/brain-cns-cancer-qpis/consult_view/.

15. Scottish Cancer Taskforce. Brain and Central Nervous System Cancer Clinical Quality Performance Indicatores V2021 2021 [Available from: https://www.healthcareimprovementscotland.org/our_work/cancer_care_improvement/cancer_qpis/quality_performance_indicators.aspx.

16. Fouke SJ, Benzinger T, Gibson D, Ryken TC, Kalkanis SN, Olson JJ. The role of imaging in the management of adults with diffuse low grade glioma: A systematic review and evidence-based clinical practice guideline. Journal of neuro-oncology. 2015;125(3):457-79.

17. Liang S, Fan X, Zhao M, Shan X, Li W, Ding P, et al. Clinical practice guidelines for the diagnosis and treatment of adult diffuse glioma-related epilepsy. Cancer Med. 2019;8(10):4527-35.

18. Martínez-Garcia M, Álvarez-Linera J, Carrato C, Ley L, Luque R, Maldonado X, et al. SEOM clinical guidelines for diagnosis and treatment of glioblastoma (2017). Clin Transl Oncol. 2018;20(1):22-8.

19. Olson JJ, Fadul CE, Brat DJ, Mukundan S, Ryken TC. Management of newly diagnosed glioblastoma: guidelines development, value and application. Journal of neuro-oncology. 2009;93(1):1-23.

20. Belgian College of Oncology. National Clinical Practice Guidelines Neuro-Oncology version 1.2008. 2008.

21. Vanhauwaert D, Pinson H, Sweldens C, Du Four S, Van Eycken L, De Schutter H, et al. Quality indicators in neuro-oncology: review of the literature and development of a new quality indicator set for glioma care through a two-round Delphi survey. Journal of neuro-oncology. 2022.

22. Liaquat I, Calmpbell L, New D. Brain and Central Nervous System Cancers Quality Performance Indicators. Report of the 2018 Clinical Audit Data. 2019 [

23. Dumba M, Fry A, Shelton J, Booth T, Jones B, Shuaib H, et al. Imaging in patients with glioblastoma: A national cohort study. Neuro-Oncology Practice. 2022.

24. Rasmussen BK, Hansen S, Laursen RJ, Kosteljanetz M, Schultz H, Norgard BM, et al. Epidemiology of glioma: clinical characteristics, symptoms, and predictors of glioma patients grade I-IV in the the Danish Neuro-Oncology Registry. J Neurooncol. 2017;135(3):571-9.

25. Asklund T, Malmstrom A, Bergqvist M, Bjor O, Henriksson R. Brain tumours in Sweden: data from a population-based registry 1999-2012. Acta Oncol. 2015;54(3):377-84.

26. Liaquat I, Campbell L, McMahon J. Audit Report: Brain and Central Nervous System Cancers Quality Performance Indicators. Report of the 2020 Clinical Audit Data. Scottish Adult Neuro Oncology Network,; 2022. p. 41.

## Amino acid PET incorporated in biopsy target (X02)

| Title | Proportion of patients with suspected low grade glioma in whom nuclear imaging with amino acid PET was incorporated in defining target for biopsy |
| --- | --- |
| Rationale | Incorporation of nuclear imaging with amino acid PET in defining the target for (stereotactic) biopsy in patients with a suspected low grade glioma can reduce the risk of sampling bias and underestimation of the tumour grade (1-3). |
| Type of indicator | Process indicator without benchmarking (‘descriptive’ indicator) |
| Reformulation | Proportion of patients with low grade (grade 2) glioma in whom nuclear imaging with PET was performed before a diagnostic biopsy |
| Calculation | Numerator: Low grade glioma patients undergoing PET from 12 weeks before until the day before a diagnostic biopsy  Denominator: Low grade (grade 2) glioma patients who underwent a diagnostic biopsy |
| Target | No target.  (Given the absence of clear criteria in the literature about the proportion of low grade glioma patients who would benefit from a PET before biopsy, this process indicator is considered as purely descriptive (without calculation of the national average, target and benchmarking).) |
| Data source | Belgian Cancer Registry (BCR): incidence years 2016-2019 (main study period) and incidence years 2012-2015 and 2008-2011 (comparison periods for sensitivity analysis)  IMA data: 2015-2021(Q2) |
| Technical definition | - Diagnosis of low grade (grade 2) glioma: ICD-O-3: 9400/3; 9410/3; 9411/3; 9420/3; 9450/3 (C71) - Incidence date as registered at BCR (= date of first microscopic confirmation of malignancy, if not available, the incidence date is determined in decreasing priority by 1) first hospitalization for cancer, 2) first consultation because of malignancy, 3) initial clinical or technical diagnosis, 4) start of treatment for cancer and 5) death) - Diagnostic biopsy - Timeframe: from 3 months before until 3 months after incidence date (and before surgical resection in case surgical resection was performed): nomenclature codes (IMA, table 1) - Oncological treatment - Surgical resection (timeframe: from 1 month before until 9 months after incidence date. Note: in case a biopsy was followed by chemo- and/or radiotherapy and subsequently by a surgical resection, the surgical resection was not withheld as primary treatment): nomenclature codes (IMA, table 2) - Chemotherapy (timeframe in the presence of surgical resection: from day of surgical resection until 12 months after surgical resection (adjuvant); timeframe in the absence of surgical resection: from 1 month before until 9 months after incidence date): ATC codes (IMA, table 3) - Radiotherapy (timeframe in the presence of surgical resection: from day of surgical resection until 12 months after surgical resection (adjuvant); timeframe in the absence of surgical resection: from 1 month before until 9 months after incidence date): nomenclature codes (IMA, table 4) - Multiple tumours   If additional tumours around the diagnosis of the glioma (i.e. from 5 years before to maximum 2 years after the incidence date of the glioma) are present (Note: only information about tumours with an incidence <= the year 2021 is available)   - Inside the CNS, benign, borderline or malignant tumours are taken into account - Outside the CNS, only malignant tumours are considered   - Comorbidities (diabetes, cardiovascular disease and respiratory disease)   - Calculation is based on medication use in the year prior to the glioma diagnosis - Per comorbidity, a set of ATC codes is withheld (IMA, table 5). If the annual use of this set of ATC codes by a patient exceeds a certain threshold, this comorbidity is considered to be present - PET prior to diagnostic biopsy - Timeframe: from 12 weeks before until the day before diagnostic biopsy: nomenclature codes (IMA, table 9) - Tracer administration: pseudonomenclature codes (IMA table 10) - Alternative imaging prior to diagnostic biopsy - Timeframe CT: from 12 weeks before until the day before diagnostic biopsy: nomenclature codes (IMA, table 11) - Timeframe MRI: from 12 weeks before until the day before diagnostic biopsy: nomenclature codes (IMA, table 7) |
| Risk adjustment | None (descriptive indicator) |
| Limitations | Patients with a suspected tumour cannot be identified based on the available data. Only patients with confirmed brain tumours (through histopathology or imaging) are registered and are amenable for assessment of indicators. Therefore, the term “suspected” is omitted in the formulation of the indicator.  Moreover, patients with a “suspected” low grade glioma where the biopsy reveals a higher grade tumour are not included in the denominator, since patient selection is based on histological confirmation and therefore the denominator only consists of confirmed grade 2 tumours.  The incorporation of amino acid PET into the planning and targeting for (stereotactic) biopsy cannot be identified, only the performance of amino acid PET prior to the biopsy is known.  The use of radiopharmaceuticals is difficult to identify (which also emerged during the validation phase of this study), among others since they are often produced in-house. ATC codes for diagnostic radiopharmaceuticals (V09IX) cannot be found in the IMA database. Pseudo-nomenclature codes for diagnostic radioisotopes are available but are underreported. Therefore it is not in all cases possible to identify whether a 18F-FDG-PET or Amino Acid-PET (11C-Methionine, 18F-FET,…) isotope was used.  For assessing the use of amino-acid PET in low grade glioma, the multidisciplinary expert board charged with the validation of the technical documentation sheets, judged that, based on absence of clear criteria in the literature, it is impossible to delineate which proportion of low grade glioma patients would benefit from an amino-acid PET to define a target. Therefore, this indicator remains a pure descriptive indicator without mean, target and benchmarking. |
| Subgroup analyses | - Year of incidence - Age at time of diagnosis - Sex - Multiple tumours - WHO performance status - Comorbidities (diabetes, cardiovascular disease, respiratory disease) - Treatment scheme |
| Sensitivity analyses | - Incidence year 2008-2011 vs 2012-2015 vs 2016-2019 - Tracer administration (PET with amino acid tracer versus PET with glucose tracer versus PET with no tracer) - PET with different timeframes before biopsy - Type of imaging (MRI, PET and/or CT) before diagnostic biopsy |
| Benchmarking | Centre of biopsy |
| International results | / |

References

1. Pirotte B, Goldman S, Brucher JM, Zomosa G, Baleriaux D, Brotchi J, et al. PET in stereotactic conditions increases the diagnostic yield of brain biopsy. Stereotact Funct Neurosurg. 1994;63(1-4):144-9.

2. Kunz M, Thon N, Eigenbrod S, Hartmann C, Egensperger R, Herms J, et al. Hot spots in dynamic (18)FET-PET delineate malignant tumour parts within suspected WHO grade II gliomas. Neuro Oncol. 2011;13(3):307-16.

3. Albert NL, Weller M, Suchorska B, Galldiks N, Soffietti R, Kim MM, et al. Response Assessment in Neuro-Oncology working group and European Association for Neuro-Oncology recommendations for the clinical use of PET imaging in gliomas. Neuro-oncology. 2016;18(9):1199-208.

## MRI FULL SPINE EPENDYMOMA (D03)

| Title | Proportion of patients with diagnosis of intracranial ependymoma who had full spine MRI |
| --- | --- |
| Rationale | Because of the risk of dissemination of ependymoma through the cerebrospinal fluid (CSF), staging of the disease by MRI of the entire neuroaxis is highly recommended. There is no consensus on the timing of spinal MRI with regards to surgery, but it is recommended before the start of adjuvant treatment. CSF dissemination occurs in 3-15% of ependymomas and is present at time of diagnosis in less than 5% (1). Prognosis and treatment are defined based on grade of the tumour and the stage of the disease (2-5). |
| Type of indicator | Process indicator with benchmarking |
| Reformulation | Proportion of patients with diagnosis of intracranial ependymoma who had (full) spine MRI |
| Calculation | Numerator: Intracranial ependymoma patients who received (full) spine MRI from 1 month before until 6 weeks after incidence date  Denominator: Intracranial ependymoma patients |
| Target | 90 %  Tolerance for patients with medical devices where MRI is contra-indicated |
| Data source | Belgian Cancer Registry (BCR): incidence years 2016-2019 (main study period) and incidence years 2012-2015 and 2008-2011 (comparison periods for sensitivity analysis)  IMA data: 2015-2021(Q2) |
| Technical definition | - Diagnosis of ependymoma: ICD-O-3: 9391/3; 9392/3; 9393/3; 9396/3 (C71) - Incidence date as registered at BCR (= date of first microscopic confirmation of malignancy, if not available, the incidence date is determined in decreasing priority by 1) first hospitalization for cancer, 2) first consultation because of malignancy, 3) initial clinical or technical diagnosis, 4) start of treatment for cancer and 5) death) - Diagnostic biopsy - Timeframe: from 3 months before until 3 months after incidence date (and before surgical resection in case surgical resection was performed): nomenclature codes (IMA, table x) - Oncological treatment - Surgical resection (timeframe: from 1 month before until 9 months after incidence date. Note: in case a biopsy was followed by chemo- and/or radiotherapy and subsequently by a surgical resection, the surgical resection was not withheld as primary treatment): nomenclature codes (IMA, table 2) - Chemotherapy (timeframe in the presence of surgical resection: from day of surgical resection until 12 months after surgical resection (adjuvant); timeframe in the absence of surgical resection: from 1 month before until 9 months after incidence date): ATC codes (IMA, table 3) - Radiotherapy (timeframe in the presence of surgical resection: from day of surgical resection until 12 months after surgical resection (adjuvant); timeframe in the absence of surgical resection: from 1 month before until 9 months after incidence date): nomenclature codes (IMA, table 4) - Multiple tumours   If additional tumours around the diagnosis of the glioma (i.e. from 5 years before to maximum 2 years after the incidence date of the glioma) are present (Note: only information about tumours with an incidence <= the year 2021 is available)   - Inside the CNS, benign, borderline or malignant tumours are taken into account - Outside the CNS, only malignant tumours are considered   - Comorbidities (diabetes, cardiovascular disease and respiratory disease)   - Calculation is based on medication use in the year prior to the glioma diagnosis - Per comorbidity, a set of ATC codes is withheld (IMA, table x). If the annual use of this set of ATC codes by a patient exceeds a certain threshold, this comorbidity is considered to be present - MRI (full spine or segments of the spine) - Timeframe: from 1 month before until 6 weeks after incidence date - Nomenclature codes (IMA, table 7) - <2019: 459491/459502 (at least one part of the spine) - 2019: 457951/457962 (full spine) OR combination of at least two different codes of 459491/459502-457914/457925-457936/457940 (~full spine) - Alternative imaging - Timeframe CT vertebral column: from 1 month before until 6 weeks after incidence date: nomenclature codes (IMA, table 11) |
| Risk adjustment | None (process indicator) |
| Limitations | Before December 1, 2018 there was only 1 nomenclature code for MRI of the spine that could be used for MRI of the cervical, thoracic, lumbosacral or full spine. Consequently, it can only be assessed whether at least once this code was used to evaluate (parts of) the spinal cord (see also table 7).  After December 1, 2018 different codes were introduced for different segments of the spine. In clinical practice, imaging of the thoracic spine is sometimes covered by a combination of an MRI of the cervical and the lumbar spine, each visualising half of the thoracic spinal cord. Therefore, the following combinations are considered to cover MRI of the entire neuraxis: MRI full spine OR (MRI cervical spine + MRI thoracic spine + MRI lumbosacral spine) OR (MRI cervical spine + MRI lumbosacral spine). Because of the time frame allowed for the MRI scan, this was only evaluated for patients with an incidence date in 2019. |
| Subgroup analyses | - Year of incidence - Age at time of diagnosis - Sex - Multiple tumours - WHO performance status - Comorbidities (diabetes, cardiovascular disease, respiratory disease) - Treatment scheme |
| Sensitivity analyses | - Incidence year 2008-2011 vs 2012-2015 vs 2016-2019 - (Full) spine MRI with different timeframes around incidence date - Type of (full) spine imaging (MRI, CT) |
| Benchmarking | Centre of main treatment |
| International results | / |

References

1. Ruda R, Gilbert M, Soffietti R. Ependymomas of the adult: molecular biology and treatment. Curr Opin Neurol. 2008;21(6):754-61.

2. Ruda R, Reifenberger G, Frappaz D, Pfister SM, Laprie A, Santarius T, et al. EANO guidelines for the diagnosis and treatment of ependymal tumours. Neuro Oncol. 2018;20(4):445-56.

3. Vanhauwaert D, Pinson H, Sweldens C, Du Four S, Van Eycken L, consortium Q, et al. Quality indicators in neuro-oncology: Review of the literature and development of a new quality indicator set for glioma care through a two-round Delphi survey. J Neurooncol. 2022(2):365-76.

4. Louis DN, Perry A, Reifenberger G, von Deimling A, Figarella-Branger D, Cavenee WK, et al. The 2016 World Health Organization Classification of Tumours of the Central Nervous System: a summary. Acta Neuropathol. 2016;131(6):803-20.

5. Landelijke werkgroep neuro-oncologie. Ependymoma in Adults. Evaluation and Treatment Protocol. Utrecht2019 [

:[Available from: https://lwno.nl/media/1075/treatment-protocol-ependymoma_20191010_definitief.pdf.

## FOLLOW-UP MRI LGG (F02)

| Title | Proportion of patients with low grade glioma undergoing follow-up with MRI 2 to 4 times yearly in the first 5 years and 1 to 2 times yearly after the first 5 years after diagnosis. MRI should include T2/flair and T1 with and without contrast and volumetric assessment of residual tumour |
| --- | --- |
| Rationale | Because of the risk of evolution of a low grade (grade 2) glioma into a high grade (grade 3/4) variant, follow-up with sequential MRI is indicated. In the first 5 years after diagnosis, an MRI is recommended every 3 to 6 months, or when clinically indicated. After 5 years, this is every 6 to 12 months (1-5). |
| Type of indicator | Process indicator with benchmarking |
| Reformulation | Proportion of patients with low grade (grade 2) glioma undergoing at least two MRI’s (MRI brain or fMRI) in the first year of follow-up |
| Calculation | Numerator: Low grade (grade 2) glioma patients with at least 1 year of follow-up in IMA-data undergoing at least two MRI’s (MRI brain or fMRI) in the first follow-up year  Denominator: Low grade (grade 2) glioma patients with at least 1 year of follow-up in IMA-data (follow-up starting from start date of oncological treatment or from day of diagnostic biopsy in the absence of oncological treatment) |
| Target | 90% (Tolerance for patients with MRI incompatible medical devices) |
| Data source | Belgian Cancer Registry (BCR): incidence years 2016-2019 (main study period) and incidence years 2012-2015 and 2008-2011 (comparison periods for sensitivity analysis)  IMA data: 2015-2021(Q2)  Crossroads Bank of Social Security (Kruispuntbank van de Sociale Zekerheid (KSZ) - Banque Carrefour de la Sécurité Sociale (BCSS)) for vital status: follow up until 2023/01/16 |
| Technical definition | - Diagnosis of low grade (grade 2) glioma: ICD-O-3: 9400/3; 9410/3; 9411/3; 9420/3; 9450/3 (C71) - Incidence date as registered at BCR (= date of first microscopic confirmation of malignancy, if not available, the incidence date is determined in decreasing priority by 1) first hospitalization for cancer, 2) first consultation because of malignancy, 3) initial clinical or technical diagnosis, 4) start of treatment for cancer and 5) death) - Diagnostic biopsy - Timeframe: from 3 months before until 3 months after incidence date (and before surgical resection in case surgical resection was performed): nomenclature codes (IMA, table x) - Oncological treatment - Surgical resection (timeframe: from 1 month before until 9 months after incidence date. Note: in case a biopsy was followed by chemo- and/or radiotherapy and subsequently by a surgical resection, the surgical resection was not withheld as primary treatment): nomenclature codes (IMA, table 2) - Chemotherapy (timeframe in the presence of surgical resection: from day of surgical resection until 12 months after surgical resection (adjuvant); timeframe in the absence of surgical resection: from 1 month before until 9 months after incidence date): ATC codes (IMA, table 3) - Radiotherapy (timeframe in the presence of surgical resection: from day of surgical resection until 12 months after surgical resection (adjuvant); timeframe in the absence of surgical resection: from 1 month before until 9 months after incidence date): nomenclature codes (IMA, table 4) - Multiple tumours:   If additional tumours around the diagnosis of the glioma (i.e. from 5 years before to maximum 2 years after the incidence date of the glioma) are present (Note: only information about tumours with an incidence <= the year 2021 is available)   - Inside the CNS, benign, borderline or malignant tumours are taken into account - Outside the CNS, only malignant tumours are considered   - Comorbidities (diabetes, cardiovascular disease and respiratory disease)   - Calculation is based on medication use in the year prior to the glioma diagnosis - Per comorbidity, a set of ATC codes is withheld (IMA, table 5). If the annual use of this set of ATC codes by a patient exceeds a certain threshold, this comorbidity is considered to be present - Follow-up MRI (MRI brain or fMRI): - Timeframe: from start date of oncological treatment or from day of biopsy in the absence of an oncological treatment: nomenclature codes (IMA, table 7) - Availability of follow-up data: - Calculated since start date of oncological treatment (or since date of diagnostic biopsy in the absence of oncological treatment) - Both survival time of the patient and IMA availability are considered |
| Risk adjustment | None (process indicator) |
| Limitations | Different MRI sequences (T1, T2…) cannot be defined from the administrative databases.  IMA data of cancer patients are only available at the BCR for the period of 1 January of the year preceding the incidence year until 31 December of the 5th year following the incidence year. Therefore, follow-up more than 5 years after diagnosis cannot be assessed.  IMA data become available with a delay of approximately 2 years. IMA data available at the BCR are considered to be 98 % complete till the end of the second quarter of 2021.  Since the number of MRI scans is calculated from the start date of oncological treatment (if oncological treatment was given), an immediate post-surgical MRI scan (within first 3 days) is also counted in the numerator of this indicator. An immediate postoperative MRI scan can be considered as follow-up after surgical resection. |
| Subgroup analyses | - Year of incidence - Age at time of diagnosis - Sex - Multiple tumours - WHO performance status - Comorbidities (diabetes, cardiovascular disease, respiratory disease) - Treatment scheme |
| Sensitivity analyses | - Incidence year 2008-2011 vs 2012-2015 vs 2016-2019 - Follow-up with different number of MRI’s in the first follow-up year   - Follow-up with MRI in consecutive years after start of oncological treatment or biopsy (first year versus second year versus third year)  - Type of MRI (only MRI brain versus only fMRI versus both MRI brain and fMRI)  - IDH molecular status of a subset of low grade (grade 2) glioma i.e. diffuse astrocytoma |
| Benchmarking | Centre of main treatment |
| International results | / |

References

1. Soffietti R. Baumert BG. Bello L. von Deimling A. Duffau H. Frenay M. et al. Guidelines on the Management of Low-grade Gliomas: EANOTask Force Report. Eur Assoc Neurooncol Mag. 2011;1 (1):37–44.

2. Soffietti R. Baumert BG. Bello L. von Deimling A. Duffau H. Frenay M. et al. Guidelines on management of low-grade gliomas: report of an EFNS-EANO Task Force. Eur J Neurol. 2010;17(9):1124-33.

3. National Comprehensive Cancer Network. Central Nervous System Cancers 2019 [Available from: https://www.nccn.org/professionals/physician_gls/pdf/cns.pdf.

4. Vanhauwaert D. Pinson H. Sweldens C. Du Four S. Van Eycken L. consortium Q. et al. Quality indicators in neuro-oncology: Review of the literature and development of a new quality indicator set for glioma care through a two-round Delphi survey. J Neurooncol. 2022.

5. Belgian College of Oncology. National Clinical Practice Guidelines Neuro-Oncology version 1.2008. 2008.

## FOLLOW-UP MRI HGG (F03)

| Title | Proportion of patients with high grade glioma undergoing follow-up with MRI with volumetric assessment of residual tumour every 2 to 4 months |
| --- | --- |
| Rationale | After resection of a high grade glioma, close iconographic follow-up is recommended to evaluate treatment and to diagnose recurrent disease (1-6). |
| Type of indicator | Process indicator with benchmarking |
| Reformulation | Proportion of patients with high grade (grade 3/4) glioma undergoing at least three MRI’s (MRI brain or fMRI) in the first year of follow-up |
| Calculation | Numerator: High grade (grade 3/4) glioma patients with at least 1 year of follow-up in IMA-data undergoing at least three MRI’s (MRI brain or fMRI) in the first follow-up year  Denominator: High grade (grade 3/4) glioma patients with at least 1 year of follow-up in IMA-data (follow-up starting from start date of oncological treatment or from day of diagnostic biopsy in the absence of oncological treatment) |
| Target | 90% (Tolerance for patients with MRI incompatible medical devices) |
| Data source | Belgian Cancer Registry (BCR): incidence years 2016-2019 (main study period) and incidence years 2012-2015 and 2008-2011 (comparison periods for sensitivity analysis)  IMA data: 2015-2021(Q2)  Crossroads Bank of Social Security (Kruispuntbank van de Sociale Zekerheid (KSZ) - Banque Carrefour de la Sécurité Sociale (BCSS)) for vital status: follow up until 2023/01/16 |
| Technical definition | - Diagnosis of high grade (grade 3/4) glioma: ICD-O-3: 9401/3; 9440/3; 9441/3; 9442/3; 9445/3; 9451/3; 9385/3 (C71) - Incidence date as registered at BCR (= date of first microscopic confirmation of malignancy, if not available, the incidence date is determined in decreasing priority by 1) first hospitalization for cancer, 2) first consultation because of malignancy, 3) initial clinical or technical diagnosis, 4) start of treatment for cancer and 5) death) - Diagnostic biopsy - Timeframe: from 3 months before until 3 months after incidence date (and before surgical resection in case surgical resection was performed): nomenclature codes (IMA, table 1) - Oncological treatment - Surgical resection (timeframe: from 1 month before until 9 months after incidence date. Note: in case a biopsy was followed by chemo- and/or radiotherapy and subsequently by a surgical resection, the surgical resection was not withheld as primary treatment): nomenclature codes (IMA, table 2) - Chemotherapy (timeframe in the presence of surgical resection: from day of surgical resection until 12 months after surgical resection (adjuvant); timeframe in the absence of surgical resection: from 1 month before until 9 months after incidence date): ATC codes (IMA, table 3) - Radiotherapy (timeframe in the presence of surgical resection: from day of surgical resection until 12 months after surgical resection (adjuvant); timeframe in the absence of surgical resection: from 1 month before until 9 months after incidence date): nomenclature codes (IMA, table 4) - Multiple tumours:   If additional tumours around the diagnosis of the glioma (i.e. from 5 years before to maximum 2 years after the incidence date of the glioma) are present (Note: only information about tumours with an incidence <= the year 2021 is available)   - Inside the CNS, benign, borderline or malignant tumours are taken into account - Outside the CNS, only malignant tumours are considered   - Comorbidities (diabetes, cardiovascular disease and respiratory disease)   - Calculation is based on medication use in the year prior to the glioma diagnosis - Per comorbidity, a set of ATC codes is withheld (IMA, table 5). If the annual use of this set of ATC codes by a patient exceeds a certain threshold, this comorbidity is considered to be present - Follow-up MRI (MRI brain or fMRI): - Timeframe: from start date of oncological treatment or from day of biopsy in the absence of an oncological treatment: nomenclature codes (IMA, table 7) - Availability of follow-up data: - Calculated since start date of oncological treatment (or since date of diagnostic biopsy in the absence of oncological treatment) - Both survival time of the patient and IMA availability are considered |
| Risk adjustment | None (process indicator) |
| Limitations | Volumetric assessment of residual tumour on MRI cannot be defined from administrative databases  IMA data of cancer patients are only available at the BCR for the period of 1 January of the year preceding the incidence year until 31 December of the fifth year following the year of incidence. Therefore, follow-up more than 5 years after diagnosis cannot be assessed.  Complete IMA data are available with a delay of approximately 2 years. So, for the latest incidence years, reliable IMA data are available till end of second quarter of 2021.  Since the number of MRI scans is calculated from the start date of oncological treatment (if oncological treatment was given), an immediate post-surgical MRI scan (within first 3 days) is also counted in the numerator of this indicator. An immediate postoperative MRI scan can be considered as follow-up after surgical resection. |
| Subgroup analyses | - Year of incidence - Age at time of diagnosis - Sex - Multiple tumours - WHO performance status - Comorbidities (diabetes, cardiovascular disease, respiratory disease) - Treatment scheme |
| Sensitivity analyses | - Incidence year 2008-2011 vs 2012-2015 vs 2016-2019 - Follow-up with different number of MRI’s in the first follow-up year   - Follow-up with MRI in consecutive years after start of oncological treatment or biopsy (first year versus second year versus third year)  - Type of MRI (only MRI brain versus only fMRI versus both MRI brain and fMRI) |
| Benchmarking | Centre of main treatment |
| International results | / |

References

1. Balañá C, Alonso M, Hern, ez-Lain A, Hern, ez A, et al. SEOM clinical guidelines for anaplastic gliomas (2017). Clin Transl Oncol. 2018;20(1):16-21.

2. Balañá C, Alonso M, Hern, ez-Lain A, Perez-Segura P, Pineda E, et al. Correction to: SEOM clinical guidelines for anaplastic gliomas (2017). Clin Transl Oncol. 2018;20(7):937-.

3. Kim YZ, Kim CY, Lim J, Sung KS, Lee J, Oh HJ, et al. The Korean Society for Neuro-Oncology (KSNO) Guideline for Glioblastomas: Version 2018.01. Brain Tumour Res Treat. 2019;7(1):1-9.

4. Kim YZ, Kim CY, Lim J, Sung KS, Lee J, Oh HJ, et al. The Korean Society for Neuro-Oncology (KSNO) Guideline for WHO Grade III Cerebral Gliomas in Adults: Version 2019.01. Brain Tumour Res Treat. 2019;7(2):63-73.

5. Vanhauwaert D, Pinson H, Sweldens C, Du Four S, Van Eycken L, consortium Q, et al. Quality indicators in neuro-oncology: Review of the literature and development of a new quality indicator set for glioma care through a two-round Delphi survey. J Neurooncol. 2022(2):365-76.

6. Belgian College of Oncology. National Clinical Practice Guidelines Neuro-Oncology version 1.2008. 2008.

## FOLLOW-UP EPENDYMOMA (F04)

| Title | Proportion of patients with ependymoma undergoing follow-up with MRI with volumetric assessment of residual tumour every 3 months for the first 2 years after diagnosis and every 6 months after the first 2 years after diagnosis |
| --- | --- |
| Rationale | Because of the risk for ependymoma patients of asymptomatic and/or late relapses, patients should be followed long term with an enhanced MRI (1-4). |
| Type of indicator | Process indicator with benchmarking |
| Reformulation | Proportion of patients with ependymoma undergoing at least three MRI’s (MRI brain or fMRI) in the first year of follow-up |
| Calculation | Numerator: Ependymoma patients with at least 1 year of follow-up in IMA-data undergoing at least three MRI’s (MRI brain or fMRI) in the first follow-up year  Denominator: Ependymoma patients with at least 1 year of follow-up in IMA-data (follow-up starting from start date of oncological treatment or from day of diagnostic biopsy in the absence of oncological treatment) |
| Target | 90% (Tolerance for patients with MRI incompatible medical devices) |
| Data source | Belgian Cancer Registry (BCR): incidence years 2016-2019 (main study period) and incidence years 2012-2015 and 2008-2011 (comparison periods for sensitivity analysis)  IMA data: 2015-2021(Q2)  Crossroads Bank of Social Security (Kruispuntbank van de Sociale Zekerheid (KSZ) - Banque Carrefour de la Sécurité Sociale (BCSS)) for vital status: follow up until 2023/01/16 |
| Technical definition | - Diagnosis of ependymoma: ICD-O-3: 9391/3; 9392/3; 9393/3; 9396/3 (C71) - Incidence date as registered at BCR (= date of first microscopic confirmation of malignancy, if not available, the incidence date is determined in decreasing priority by 1) first hospitalization for cancer, 2) first consultation because of malignancy, 3) initial clinical or technical diagnosis, 4) start of treatment for cancer and 5) death) - Diagnostic biopsy - Timeframe: from 3 months before until 3 months after incidence date (and before surgical resection in case surgical resection was performed): nomenclature codes (IMA, table 1) - Oncological treatment - Surgical resection (timeframe: from 1 month before until 9 months after incidence date. Note: in case a biopsy was followed by chemo- and/or radiotherapy and subsequently by a surgical resection, the surgical resection was not withheld as primary treatment): nomenclature codes (IMA, table 2) - Chemotherapy (timeframe in the presence of surgical resection: from day of surgical resection until 12 months after surgical resection (adjuvant); timeframe in the absence of surgical resection: from 1 month before until 9 months after incidence date): ATC codes (IMA, table 3) - Radiotherapy (timeframe in the presence of surgical resection: from day of surgical resection until 12 months after surgical resection (adjuvant); timeframe in the absence of surgical resection: from 1 month before until 9 months after incidence date): nomenclature codes (IMA, table 4) - Multiple tumours:   If additional tumours around the diagnosis of the glioma (i.e. from 5 years before to maximum 2 years after the incidence date of the glioma) are present (Note: only information about tumours with an incidence <= the year 2021 is available)   - Inside the CNS, benign, borderline or malignant tumours are taken into account - Outside the CNS, only malignant tumours are considered   - Comorbidities (diabetes, cardiovascular disease and respiratory disease)   - Calculation is based on medication use in the year prior to the glioma diagnosis - Per comorbidity, a set of ATC codes is withheld (IMA, table 5). If the annual use of this set of ATC codes by a patient exceeds a certain threshold, this comorbidity is considered to be present - Follow-up MRI (MRI brain or fMRI): - Timeframe: from start date of oncological treatment or from day of biopsy in the absence of an oncological treatment: nomenclature codes (IMA, table 7) - Availability of follow-up data: - Calculated since start date of oncological treatment (or since date of diagnostic biopsy in the absence of oncological treatment) - Both survival time of the patient and IMA availability are considered |
| Risk adjustment | None (process indicator) |
| Limitations | Volumetric assessment of residual tumour on MRI cannot be defined from administrative databases  IMA data of cancer patients are only available at the BCR for the period of 1 January of the year preceding the incidence year until 31 December of the fifth year following the year of incidence. Therefore, follow-up more than 5 years after diagnosis cannot be assessed.  Complete IMA data are available with a delay of approximately 2 years. So, for the latest incidence years, reliable IMA data are available till end of second quarter of 2021.  Since the number of MRI scans is calculated from the start date of oncological treatment (if oncological treatment was given), an immediate post-surgical MRI scan (within first 3 days) is also counted in the numerator of this indicator. An immediate postoperative MRI scan can be considered as follow-up after surgical resection. |
| Subgroup analyses | - Year of incidence - Age at time of diagnosis - Sex - Multiple tumours - WHO performance status - Comorbidities (diabetes, cardiovascular disease, respiratory disease) - Treatment scheme |
| Sensitivity analyses | - Incidence year 2008-2011 vs 2012-2015 vs 2016-2019 - Follow-up with different number of MRI’s in the first follow-up year - Follow-up with MRI in first follow-up period (prolonging first year: 365 vs 375 vs 385 vs 395 days) - Follow-up with MRI in consecutive years after start of oncological treatment or biopsy (first year versus second year versus third year) - Type of MRI (only MRI brain versus only fMRI versus both MRI brain and fMRI) |
| Benchmarking | Centre of main treatment |
| International results | / |

References

1. Vanhauwaert D, Pinson H, Sweldens C, Du Four S, Van Eycken L, consortium Q, et al. Quality indicators in neuro-oncology: Review of the literature and development of a new quality indicator set for glioma care through a two-round Delphi survey. J Neurooncol. 2022.

2. Delgado-López PD, Corrales-García EM, Alonso-García E, García-Leal R, González-Rodrigálvarez R, Araus-Galdós E, et al. Central nervous system ependymoma: clinical implications of the new molecular classification, treatment guidelines and controversial issues. Clin Transl Oncol. 2019;21(11):1450-63.

3. Ruda R, Reifenberger G, Frappaz D, Pfister SM, Laprie A, Santarius T, et al. EANO guidelines for the diagnosis and treatment of ependymal tumours. Neuro Oncol. 2018;20(4):445-56.

4. National Comprehensive Cancer Network. Central Nervous System Cancers 2019 [Available from: https://www.nccn.org/professionals/physician_gls/pdf/cns.pdf.

## IMMEDIATE POSTOP MRI IN HGG (F01)

| Title | Proportion of patients with HGG who had postoperative MRI in which volumetric assessment of residual tumour is performed within 72 hours after surgical resection |
| --- | --- |
| Rationale | Postoperative imaging provides an estimation of the postoperative volume, and yet prognosis. It helps to determine what further treatment is appropriate and it can help target radiotherapy. It also provides a measurement of surgical performance (1-6). |
| Type of indicator | Process indicator with benchmarking |
| Reformulation | Proportion of patients with high grade (grade 3/4) glioma who had postoperative MRI (MRI brain or fMRI) within 72 hours after surgical resection |
| Calculation | Numerator: High grade (grade 3/4) glioma patients who underwent a surgical resection undergoing a postoperative MRI (MRI brain or fMRI) performed within the first 3 days after surgical resection  Denominator: All high grade (grade 3/4) glioma patients who underwent a surgical resection |
| Target | 90%  Tolerance considers:   - Patients with implanted devices with contra-indications for MRI - Patients deemed unfit to undergo MRI |
| Data source | Belgian Cancer Registry (BCR): incidence years 2016-2019 (main study period) and incidence years 2012-2015 and 2008-2011 (comparison periods for sensitivity analysis)  IMA data: 2015-2021(Q2) |
| Technical definition | - Diagnosis of high grade (grade 3/4) glioma: ICD-O-3: 9385/3; 9401/3; 9440/3; 9441/3; 9442/3; 9445/3; 9451/3 (C71) - Incidence date as registered at BCR (= date of first microscopic confirmation of malignancy, if not available, the incidence date is determined in decreasing priority by 1) first hospitalization for cancer, 2) first consultation because of malignancy, 3) initial clinical or technical diagnosis, 4) start of treatment for cancer and 5) death) - Diagnostic biopsy - Timeframe: from 3 months before until 3 months after incidence date (and before surgical resection in case surgical resection was performed): nomenclature codes (IMA, table 1) - Oncological treatment - Surgical resection (timeframe: from 1 month before until 9 months after incidence date. Note: in case a biopsy was followed by chemo- and/or radiotherapy and subsequently by a surgical resection, the surgical resection was not withheld as primary treatment): nomenclature codes (IMA, table 2) - Chemotherapy (timeframe in the presence of surgical resection: from day of surgical resection until 12 months after surgical resection (adjuvant); timeframe in the absence of surgical resection: from 1 month before until 9 months after incidence date): ATC codes (IMA, table 3) - Radiotherapy (from day of surgical resection until 12 months after surgical resection (adjuvant); timeframe in the absence of surgical resection: from 1 month before until 9 months after incidence date): nomenclature codes (IMA, table 4) - Multiple tumours:   If additional tumours around the diagnosis of the glioma (i.e. from 5 years before to maximum 2 years after the incidence date of the glioma) are present (Note: only information about tumours with an incidence <= the year 2021 is available)   - Inside the CNS, benign, borderline or malignant tumours are taken into account - Outside the CNS, only malignant tumours are considered   - Comorbidities (diabetes, cardiovascular disease and respiratory disease)   - Calculation is based on medication use in the year prior to the glioma diagnosis - Per comorbidity, a set of ATC codes is withheld (IMA, table 5). If the annual use of this set of ATC codes by a patient exceeds a certain threshold, this comorbidity is considered to be present - Post-operative MRI (MRI brain or fMRI) - Timeframe: from day of surgical resection until maximum three days after surgical resection: nomenclature codes (IMA, table 7) - Priority is given to an MRI on one to three days after surgical resection - If no MRI on one to three days after surgical resection is reimbursed, an MRI on day of surgical resection is withheld as post-operative MRI (but only if another MRI is registered in the timeframe 6 weeks before until the day before surgical resection; which is considered then as pre-operative) - Post-operative CT - Timeframe: from day of surgical resection until maximum three days after surgical resection: nomenclature codes (IMA, table 11) - Priority is given to a CT on one to three days after surgical resection - If no CT on one to three days after surgical resection is reimbursed, a CT on day of surgical resection is withheld as post-operative CT (but only if another CT is registered in the timeframe 6 weeks before until the day before surgical resection; which is considered then as pre-operative) |
| Risk adjustment | None (process indicator) |
| Limitations | A postoperative MRI on day of surgical resection cannot be differentiated from a pre-operative (neuronavigation) MRI on day of surgical resection. Therefore, MRI on day of surgical resection is considered as a postoperative MRI, only for those patients who underwent also a pre-operative MRI within 6 weeks before surgical resection. If there is no pre-operative MRI, the MRI on day of surgical resection is considered as a diagnostic MRI or an MRI for neuronavigation. If an MRI is performed on day of surgical resection and on one to three days after surgical resection, then the MRI on day of surgical resection is considered as a preoperative MRI. |
| Subgroup analyses | - Year of incidence - Age at time of diagnosis - Sex - Multiple tumours - WHO performance status - Comorbidities (diabetes, cardiovascular disease, respiratory disease) - Treatment scheme |
| Sensitivity analyses | - Incidence year 2008-2011 vs 2012-2015 vs 2016-2019 - Postoperative MRI with different timeframes after surgical resection - Type of postoperative MRI (only MRI brain versus only fMRI versus both MRI brain and fMRI) - Type of postoperative imaging (only MRI versus only CT versus both MRI and CT) – max 3 and max 5 days after surgical resection |
| Benchmarking | Centre of surgical resection |
| International results | See Table 6.1 – International results |

Table 9.1 – International results

| Author | Period covered | Country | Results |
| --- | --- | --- | --- |
| Booth (7) | 2020 | UK | In a nationwide survey in the U.K. **87%** of neuro-oncology centers reported the **routine performance of EPMRI** (early postoperative MRI, i.e. within 72 hours of surgery) |
| Dumba (8) | 2013-2014 | England | In this national cohort study on imaging in glioblastoma patients, early (within 72 hours of surgery) **postoperative MR imaging rates in the MRI-compatible cohort** (i.e. received at least 1 MRI in the dataset) was **45%.** In the group of patients who went on to receive chemoradiation this was 51%, while for patients not receiving further oncological treatment this was 31%. |
| Hansen (6) | 2010-2014 | Denmark | In the Danish Neuro-Oncology Registry the rate of **postoperative MRI within 3 days after surgery increased from 63% in 2010 to 89% in 2014**. |
| Asklund (9) | 1999-2012 | Sweden | In Sweden, the **radiological estimation of surgical radicality** with **CT and/or MRI** for patients with high grade gliomas classified as radically or partially resected is **90%.** There are no data about the number of patients receiving only MRI after resection. |

References

1. Scottish Cancer Taskforce. Brain and Central Nervous System Cancer Clinical Quality Performance Indicators 2018 [Available from: https://consult.gov.scot/nhs/brain-cns-cancer-qpis/consult_view/.

2. Scottish Cancer Taskforce. Brain and Central Nervous System Cancer Clinical Quality Performance Indicatores V2021 2021 [Available from: https://www.healthcareimprovementscotland.org/our_work/cancer_care_improvement/cancer_qpis/quality_performance_indicators.aspx.

3. Jordan JT, ers AE, Armstrong T, Asher T, Bennett A, Dunbar E, et al. Quality improvement in neurology: Neuro-oncology quality measurement set. Neurology. 2018;90(14):652-8.

4. Jordan JT, Sanders AE, Armstrong T, Asher T, Bennett A, Dunbar E, et al. Quality improvement in neurology: Neuro-Oncology Quality Measurement Set. Neuro Oncol. 2018;20(4):531-7.

5. Hansen S. The Danish Neuro-Oncology Registry. Clinical epidemiology. 2016;8:629-32.

6. Hansen S, Nielsen J, Laursen RJ, Rasmussen BK, Norgard BM, Gradel KO, et al. The Danish Neuro-Oncology Registry: establishment, completeness and validity. BMC Res Notes. 2016;9(1):425.

7. Booth TC, Luis A, Brazil L, Thompson G, Daniel RA, Shuaib H, et al. Glioblastoma post-operative imaging in neuro-oncology: current UK practice (GIN CUP study). Eur Radiol. 2021;31(5):2933-43.

8. Dumba M, Fry A, Shelton J, Booth T, Jones B, Shuaib H, et al. Imaging in patients with glioblastoma: A national cohort study. Neuro-Oncology Practice. 2022.

9. Asklund T, Malmstrom A, Bergqvist M, Bjor O, Henriksson R. Brain tumours in Sweden: data from a population-based registry 1999-2012. Acta Oncol. 2015;54(3):377-84.

Tables

| **Table 1: nomenclature selection for diagnostic biopsy (BIOP)** | | | | | | |  | | | |  | | |  |  |  |  |  |  |  |  |
| --- | --- | --- | --- | --- | --- | --- | --- | --- | --- | --- | --- | --- | --- | --- | --- | --- | --- | --- | --- | --- | --- |
|  |  | |  | | | |  | | | |  | | |  |  |  |  |  |  |  |  |
| **code ambulatory** | **code hospitalisation** | | **dutch description** | | | | **french description** | | | | **creation date** | | | **suppression date** |  |  |  |  |  |  |  |
| 232971 | 232982 | | /*Stereotaxie van één of meer endocraniale zones, met gebruik van een rigide systeem met merktekens, gefixeerd op invasieve wijze*/ | | | | /*Stéréotaxie d'une ou de plusieurs zones endocrâniennes, au moyen d'un système de repérage rigide fixé par voie invasive*/ | | | | 1/08/1988 | | |  |  |  |  |  |  |  |  |
| 230495 | 230506 | | /*Stereotaxis van één of meer hersenzones*/ | | | | /*Stéréotaxie d'une ou de plusieurs zones cérébrales*/ | | | | 1/04/1985 | | | 1/01/2013 |  |  |  |  |  |  |  |
| 230311* | 230322* | | /*Cerebrale trepaanpunctie*/ | | | | /*Trépano-ponction cérébrale*/ | | | | 1/04/1985 | | |  |  |  |  |  |  |  |  |
| 232330* | 232341* | | /*Stereotactische implantatie van een definitieve elektrode voor diepe hersenstimulatie - unilateraal*/ | | | | /*Implantation stéréotaxique d'une électrode définitive pour la stimulation cérébrale profonde - unilatérale*/ | | | | 1/01/2012 | | |  |  |  |  |  |  |  |  |
| 232352* | 232363* | | /*Stereotactische implantatie van een definitieve elektrode voor diepe hersenstimulatie - bilateraal*/ | | | | /*Implantation stéréotaxique d'une électrode définitive pour la stimulation cérébrale profonde - bilatérale*/ | | | | 1/01/2012 | | |  |  |  |  |  |  |  |  |
| ** withheld as a diagnostic biopsy if nomenclature code is reimbursed in combination with at least one nomenclature code related to pathological anatomy/genetic testing on day 0 until day 5 after registration of the biopsy nomenclature code.* | | | | | | | | | | |  | | |  |  |  |  |  |  |  |  |
|  | | | | | | | | | | |  | | |  |  |  |  |  |  |  |  |
|  |  | |  | | | |  | | | |  | | |  |  |  |  |  |  |  |  |
| **Table 2: nomenclature selection for surgical resection (SURG)** | | | | | | |  | | | |  | | |  |  |  |  |  |  |  |  |
|  |  | |  | | | |  | | | |  | | |  |  |  |  |  |  |  |  |
| **code ambulatory** | **code hospitalisation** | | **dutch description** | | | | **french description** | | | | **creation date** | | | **suppression date** |  |  |  |  |  |  |  |
| 230473 | 230484 | | /*Heelkundige bewerking langs trepanatieluik wegens supratentoriële intracraniële expansieve processus*/ | | | | /*Intervention chirurgicale par volet de trépanation pour un processus expansif supratentoriel intracrânien*/ | | | | 1/04/1985 | | |  |  |  |  |  |  |  |  |
| 231033 | 231044 | | /*Heelkundige bewerking voor expansieve brughoekletsels of infratentoriele intracraniële expansieve processus*/ | | | | /*Intervention chirurgicale pour lésions expansives de l'angle pontocérébelleux ou processus expansif infratentoriel intracrânien*/ | | | | 1/07/1986 | | |  |  |  |  |  |  |  |  |
| 230355 | 230366 | | /*Trepanatie, decompressieve, of voor draineren*/ | | | | /*Trépanation décompressive ou pour drainage*/ | | | | 1/04/1985 | | |  |  |  |  |  |  |  |  |
| 230436 | 230440 | | /*Volledige heelkundige behandeling van een of meer intracerebrale hematomen langs groot trepanatieluik*/ | | | | /*Cure chirurgicale d'un ou plusieurs hématomes intracérébraux par grand volet de trépanation*/ | | | | 1/04/1985 | | |  |  |  |  |  |  |  |  |
| 230392 | 230403 | | /*Volledige heelkundige behandeling van een of meer extracerebrale intracraniële hematomen door eenvoudige trepanatie (breder gemaakt met holle beitel of trepaankroon), ongeacht het aantal trepaangaten*/ | | | | /*Cure chirurgicale d'un ou plusieurs hématomes intracrâniens extra cérébraux par simple trépanation (élargie à la pince gouge ou couronne tréphine) quel que soit le nombre de trous de trépan*/ | | | | 1/04/1985 | | |  |  |  |  |  |  |  |  |
| 230532 | 230543 | | /*Topectomie*/ | | | | /*Topectomie*/ | | | | 1/04/1985 | | |  |  |  |  |  |  |  |  |
| 232735* | 232746* | | /*Extra-intracraniale anastomoses : Extra-intracraniale vasculaire anastomose door microchirurgische techniek*/ | | | | /*Anastomose extra-intracrâniennes : Anastomose vasculaire extra-intra crânienne par technique microchirurgicale*/ | | | | 1/08/1988 | | |  |  |  |  |  |  |  |  |
| 232551** | 232562** | | /*Aneurysma of arterioveneuze verbinding : Heelkundige behandeling door trepanatie of laminectomie van een aneurysma of van een arterioveneuze verbinding van de hersenen, van het ruggemerg en van de meningeale omhulsels ervan*/ | | | | /*Anévrysme ou communication artérioveineuse : Traitement chirurgical par trépanation ou laminectomie d'un anévrysme ou d'une communication artérioveineuse de l'encéphale, de la moelle épinière ainsi que de leurs enveloppes méningées*/ | | | | 1/08/1988 | | |  |  |  |  |  |  |  |  |
| ** Nomenclature code is each time reimbursed in combination with at least one nomenclature code related to pathological anatomy/genetic testing on day 0 until day 5 after registration of the surgical procedure (for 7/11 patients with this nomenclature code, surgery is also validated based on pathological protocol(s))* | | | | | | | | | | | | | | |  |  |  |  |  |  |  |
| *** Nomenclature code is withheld for all patients where this code was reimbursed, except for one patient (based on checks protocol(s) and check pattern of biopsy/surgery)* | | | | | | | | | | |  | | |  |  |  |  |  |  |  |  |
|  |  | |  | | | |  | | | |  | | |  |  |  |  |  |  |  |  |
| **Table 3: ATC selection for chemotherapy (ChT)** | | | | | | | | |  |  |  |  |  |  |  |  |  |  |  |  |  |
|  | |  | | |  | | | |  |  |  |  |  |  |  |  |  |  |  |  |  |
| **Substance name** | | **ATC code** | | |  | | | |  |  |  |  |  |  |  |  |  |  |  |  |  |
| temozolomide | | L01AX03 | | |  | | | |  |  |  |  |  |  |  |  |  |  |  |  |  |
| procarbazine* | | L01XB01 | | |  | | | |  |  |  |  |  |  |  |  |  |  |  |  |  |
| lomustine /CCNU* | | L01AD02 | | |  | | | |  |  |  |  |  |  |  |  |  |  |  |  |  |
| vincristine* | | L01CA02 | | |  | | | |  |  |  |  |  |  |  |  |  |  |  |  |  |
| carmustine/BCNU | | L01AD01 | | |  | | | |  |  |  |  |  |  |  |  |  |  |  |  |  |
| ** PCV chemotherapy*  *Remark: for identification of chemotherapy prior to death all CNK codes corresponding to an L01 ATC codes are considered with the exception of 1) CNK codes corresponding to L01XD04 and 2) CNK codes corresponding with both an L01 code and one of the following two ATC codes: M01AH01 or L04AX03* | | | | | | | | | | | | | | |  | |  | |  |  |  |
| **Table 4: nomenclature selection for radiotherapy (RT)** | | | | | | |  | | | |  | | |  |  |  |  |  |  |  |  |
|  |  | |  | | | |  | | | |  | | |  |  |  |  |  |  |  |  |
| **code ambulatory** | **code hospitalisation** | | **dutch description** | | | | **french description** | | | | **creation date** | | | **suppression date** |  |  |  |  |  |  |  |
| 444113 | 444124 | | Forfaitair honorarium voor een eenvoudige uitwendige bestralingsreeks van 1 tot 10 fracties voor een patiënt die beantwoordt aan de criteria of lijdt aan een aandoening opgenomen in categorie 1 | | | | Honoraires forfaitaires pour une série d'irradiations externes simples de 1 à 10 fractions chez un patient qui répond aux critères ou pathologie repris en catégorie 1 | | | | 1/06/2001 | | |  |  |  |  |  |  |  |  |
| 444135 | 444146 | | Forfaitair honorarium voor een eenvoudige uitwendige bestralingsreeks van minstens 11 tot 35 fracties voor een patiënt die beantwoordt aan de criteria of lijdt aan een aandoening opgenomen in categorie 2 | | | | Honoraires forfaitaires pour une série d'irradiations externes simples de 11 à 35 fractions chez un patient qui répond aux critères ou pathologie repris en catégorie 2 | | | | 1/06/2001 | | |  |  |  |  |  |  |  |  |
| 444150 | 444161 | | Forfaitair honorarium voor een complexe uitwendige bestralingsreeks voor een patiënt die beantwoordt aan de criteria of lijdt aan een aandoening opgenomen in categorie 3 | | | | Honoraires forfaitaires pour une série d'irradiations externes complexes chez un patient qui répond aux critères ou pathologie repris en catégorie 3 | | | | 1/06/2001 | | |  |  |  |  |  |  |  |  |
| 444172 | 444183 | | Forfaitair honorarium voor een complexe uitwendige bestralingsreeks voor een patiënt die beantwoordt aan de criteria of lijdt aan een aandoening opgenomen in categorie 4 | | | | Honoraires forfaitaires pour une série d'irradiations externes complexes chez un patient qui répond aux critères ou pathologie repris en catégorie 4 | | | | 1/06/2001 | | |  |  |  |  |  |  |  |  |
|  |  | |  | | | |  | | | |  | | |  |  |  |  |  |  |  |  |
| **Table 5: ATC codes and required doses for comorbidity identification**   \|  \|  \|  \| \| \| \|  \|  \| \| --- \| --- \| --- \| --- \| --- \| --- \| --- \| --- \| \|  \| \| \|  \|  \| \|  \|  \|  \| \| \| \|  \|  \| \| **comorbidity** \| **ATC code** \|  \| \| \| \|  \|  \| \| Diabetes \| A10A – A10B – A10X \|  \| \| \| \|  \|  \| \| Cardiovascular disease \| C01 – C02 – C03 – C04 – C07 – C08 – C09 – B01 (exclusief B01AB) \|  \| \| \| \|  \|  \| \| respiratory disease \| R03 \|  \| \| \| \|  \|  \| \|  \|  \|  \| \| \| \|  \|  \|   **Table 6: nomenclature selection for multidisciplinary team meeting (MDT)** | | | | | | |  | | | |  | | |  |  |  |  |  |  |  |  |
|  |  | |  | | | |  | | | |  | | |  |  |  |  |  |  |  |  |
| **code ambulatory** | **code hospitalisation** | | **dutch description** | | | | **french description** | | | | **creation date** | | | **suppression date** |  |  |  |  |  |  |  |
| 350372 | 350383 | | Eerste multidisciplinair oncologisch consult (eerste MOC), geattesteerd door de geneesheer-coördinator | | | | Première consultation oncologique multidisciplinaire (première COM), attestée par le médecin-coordinateur | | | | 1/02/2003 | | |  |  |  |  |  |  |  |  |
| 350394 | 350405 | | deelname aan multidisciplinair oncologisch consult | | | | Participation à la concertation oncologique multidisciplinaire | | | | 1/02/2003 | | |  |  |  |  |  |  |  |  |
| 350453 | 350464 | | Bijkomend honorarium bij de verstrekking 350372-350383, 350276-350280 en 350291-350302 aanrekenbaar door de geneesheer-specialist in de medische oncologie, of houder van de bijzondere beroepstitel in de klinische hematologie of in de pediatrische hematologie en oncologie, wanneer deze het multidisciplinair oncologisch consult coördineert | | | | Supplément d'honoraires à la prestation 350372-350383, 350276-350280 et 350291-350302, attestable par le médecin spécialiste en oncologie médicale ou porteur du titre professionnel particulier en hématologie clinique ou en hématologie et oncologie pédiatriques, lorsque celui-ci coordonne la consultation oncologique multidisciplinaire | | | | 1/02/2003 | | |  |  |  |  |  |  |  |  |
| 350475 | 350486 | | Bijkomend honorarium bij de verstrekking 350394-350405 of 350416-350420 aanrekenbaar door de geneesheer-specialist in de medische oncologie, of houder van de bijzondere beroepstitel in de klinische hematologie of in de pediatrische hematologie en oncologie, wanneer deze het multidisciplinair oncologisch consult bijwoont | | | | Supplément d’honoraires à la prestation 350394-350405 ou 350416-350420, attestable par le médecin spécialiste en oncologie médicale ou porteur du titre professionnel particulier en hématologie clinique ou en hématologie et oncologie pédiatriques, lorsque celui-ci assiste à la consultation oncologique multidisciplinaire | | | | 1/02/2003 | | |  |  |  |  |  |  |  |  |
| 350416 | 350420 | | Deelname aan het multidisciplinair oncologisch consult door een arts die geen deel uitmaakt van de staf van ziekenhuisgeneesheren | | | | ° Participation à la concertation oncologique multidisciplinaire par un médecin qui n'est pas membre de l'équipe de médecins hospitaliers | | | | 1/02/2003 | | |  |  |  |  |  |  |  |  |
| 350276 | 350280 | | Opvolgings-multidisciplinair oncologisch consult (opvolgingsMOC), geattesteerd door de geneesheer-coördinator | | | | Concertation oncologique multidisciplinaire de suivi (COM de suivi), attestée par le médecin-coordinateur | | | | 1/11/2010 | | |  |  |  |  |  |  |  |  |
| 350291 | 350302 | | Bijkomend multidisciplinair oncologisch consult (bijkomende MOC) in een ander ziekenhuis dan dit van het eerste MOC, op doorverwijzing, geattesteerd door de geneesheercoördinator | | | | Concertation oncologique multidisciplinaire supplémentaire (COM supplémentaire) dans un hôpital autre que celui de la première COM, sur renvoi, attestée par le médecin-coordinateur | | | | 1/11/2010 | | |  |  |  |  |  |  |  |  |
|  |  | |  | | | |  | | | |  | | |  |  |  |  |  |  |  |  |
| **Table 7: nomenclature selection for magnetic resonance imaging (MRI)** | | | | | | |  | | | |  | | |  |  |  |  |  |  |  |  |
|  |  | |  | | | |  | | | |  | | |  |  |  |  |  |  |  |  |
| **code ambulatory** | **code hospitalisation** | | **dutch description** | | | | **french description** | | | | **creation date** | | | **suppression date** |  |  |  |  |  |  |  |
| ***MRI (brain)*** | | | | | | | | | | | | | | |  |  |  |  |  |  |  |
| 459395 | 459406 | | NMR-onderzoek van het hoofd (schedel, hersenen, rotsbeen, hypofyse, sinussen,orbita(e) of kaakgewrichten), minstens drie sequenties, met of zonder contrast, met registratie op optische of elektromagnetische drager | | | | Examen d'IRM de la tête (crâne, encéphale, rocher, hypophyse, sinus, orbite(s) ou articulations de la mâchoire), minimum 3 séquences avec ou sans contraste, avec enregistrement soit sur support optique, soit électromagnétique | | | | 13/08/1999 | | |  |  |  |  |  |  |  |  |
| ***fMRI*** | | | | | | | | | | | | | | |  |  |  |  |  |  |  |
| 459535 | 459546 | | Functionele MR-studie van de hersenen (BOLD-techniek) met sequentiële inzameling van de gegevens met kwantitatieve analyse via telsysteem (computer) met activiteitscurven in de tijd en/of cijfermatige tabellen en/of parametrische beelden, minstens drie sequenties, met registratie op optische of elektromagnetische drager | | | | Étude fonctionnelle par résonance magnétique de l'encéphale (technique Bold) avec collecte séquentielle des données avec analyse quantitative via un système de comptage (ordinateur) avec courbes d'activité dans le temps et/ou tableaux de mesures et/ou images paramétriques, minimum 3 séquences, avec enregistrement sur support soit optique, soit électromagnétique | | | | 13/08/1999 | | |  |  |  |  |  |  |  |  |
| ***MRI (spine)**** | | | | | | | | | | | | | | |  |  |  |  |  |  |  |
| 459491** | 459502** | | NMR-onderzoek van de cervicale wervelzuil, minstens drie sequenties, met of zonder contrast, met registratie op optische of elektromagnetische drager | | | | Examen d'IRM du rachis cervical, minimum 3 séquences, avec ou sans contraste, avec enregistrement sur support, soit optique, soit électromagnétique | | | | 13/08/1999 | | |  |  |  |  |  |  |  |  |
| 457914 | 457925 | | NMR-onderzoek van de thoracale wervelzuil, minstens drie sequenties, met of zonder contrast, met registratie op optische of elektromagnetische drager | | | | Examen d’IRM du rachis thoracique, minimum 3 séquences, avec ou sans contraste, avec enregistrement sur support, soit optique, soit électromagnétique | | | | 1/12/2018 | | |  |  |  |  |  |  |  |  |
| 457936 | 457940 | | NMR-onderzoek van de lumbosacrale wervelzuil, minstens drie sequenties, met of zonder contrast, met registratie op optische of elektromagnetische drager | | | | Examen d’IRM du rachis lombosacré, minimum 3 séquences, avec ou sans contraste, avec enregistrement sur support, soit optique, soit électromagnétique | | | | 1/12/2018 | | |  |  |  |  |  |  |  |  |
| 457951 | 457962 | | NMR-onderzoek van de volledige wervelzuil of een combinatie van twee van de NMR-onderzoeken van de cervicale, de thoracale of de lumbosacrale wervelzuil, minstens drie sequenties, met of zonder contrast, met registratie op optische of elektromagnetische drager | | | | Examen d’IRM du rachis entier ou combinaison de deux des examens d’IRM des rachis cervical, thoracique ou lombosacré , minimum 3 séquences, avec ou sans contraste, avec enregistrement sur support, soit optique, soit électromagnétique | | | | 1/12/2018 | | |  |  |  |  |  |  |  |  |
| ** These codes cannot be cumulated within a 30-day period unless a justification is included in the patient medical file*  ** The words "of thoracale of lumbosacrale" have been lifted (<12/2018: codes used for the full vertebral column or for parts of it, >=12/2018: codes only used for cervical vertebrae) | | | | | | | | | |  |  | | |  |  |  |  |  |  |  |  |
|  |  | |  | | | | | | |  |  | | |  |  |  |  |  |  |  |  |
| **Table 8: ATC code for contrast medium for magnetic resonance imaging (MRI)** | | | | | | |  | | | |  | | |  |  |  |  |  |  |  |  |
|  |  | |  | | | |  | | | |  | | |  |  |  |  |  |  |  |  |
| **Drug name** | **ATC code** | |  | | | |  | | | |  | | |  |  |  |  |  |  |  |  |
| Paramagnetisch contrastmedium | V08CA | |  | | | |  | | | |  | | |  |  |  |  |  |  |  |  |
|  |  | |  | | | |  | | | |  | | |  |  |  |  |  |  |  |  |
| **Table 9: nomenclature selection for positron emission tomography (PET)** | | | | | | |  | | | |  | | |  |  |  |  |  |  |  |  |
|  |  | |  | | | |  | | | |  | | |  |  |  |  |  |  |  |  |
| **code ambulatory** | **code hospitalisation** | | **dutch description** | | | | **french description** | | | | **creation date** | | | **suppression date** |  |  |  |  |  |  |  |
| ***PET (onco)*** | | | | | | | | | | | | | | |  |  |  |  |  |  |  |
| 442971 | 442982 | | Positronentomografisch onderzoek door coïncidentiedetectie met protocol en documenten, voor het geheel van het onderzoek, voor oncologische indicaties | | | | Tomographie à émission de positons par détection en coïncidence avec protocole et documents, pour l'ensemble de l'examen, pour des indications oncologiques | | | | 1/10/1991 | | |  |  |  |  |  |  |  |  |
|  |  | |  | | | |  | | | |  | | |  |  |  |  |  |  |  |  |
| **Table 10: pseudonomenclature selection for tracers for positron emission tomography (PET)** | | | | | | |  | | | |  | | |  |  |  |  |  |  |  |  |
|  |  | |  | | | |  | | | |  | | |  |  |  |  |  |  |  |  |
| **Drug name** | | | | **Pseudonomenclature code** | |  | |  | | | | | | |  |  | |  |  |  |  |
| ***amino acid tracer*** | | | | | |  | |  | | | | | | |  |  | |  |  |  |  |
| Methionine (C-11) | | | | 747515 | |  | |  | | | | | | |  |  | |  |  |  |  |
| Methionine (C-11) | | | | 747390 | |  | |  | | | | | | |  |  | |  |  |  |  |
| Fluoroethyltyrosine (F-18) | | | | 747250 | |  | |  | | | | | | |  |  | |  |  |  |  |
| Fluoroethyltyrosine (F-18) | | | | 746992 | |  | |  | | | | | | |  |  | |  |  |  |  |
| Fluoroethyltyrosine (F-18) | | | | 747095 | |  | |  | | | | | | |  |  | |  |  |  |  |
| Fluoroethyltyrosine (F-18) | | | | 747655 | |  | |  | | | | | | |  |  | |  |  |  |  |
| Fluoroethyltyrosine (F-18) | | | | 747003 | |  | |  | | | | | | |  |  | |  |  |  |  |
| Fluoroethyltyrosine (F-18) | | | | 747261 | |  | |  | | | | | | |  |  | |  |  |  |  |
| Methionine (C-11) | | | | 747526 | |  | |  | | | | | | |  |  | |  |  |  |  |
| Fluoroethyltyrosine (F-18) | | | | 746340 | |  | |  | | | | | | |  |  | |  |  |  |  |
| Fluoroethyltyrosine (F-18) | | | | 747106 | |  | |  | | | | | | |  |  | |  |  |  |  |
| Fluoroethyltyrosine (F-18) | | | | 746336 | |  | |  | | | | | | |  |  | |  |  |  |  |
| Fluorocholine (F-18) | | | | 747224 | |  | |  | | | | | | |  |  | |  |  |  |  |
| ***glucose tracer*** | | | | | |  | |  | | | | | | |  |  | |  |  |  |  |
| Fludeoxyglucose (F-18) | | | | 747180 | |  | |  | | | | | | |  |  | |  |  |  |  |
| Fludeoxyglucose (F-18) | | | | 747821 | |  | |  | | | | | | |  |  | |  |  |  |  |
| Fludeoxyglucose (F-18) | | | | 747176 | |  | |  | | | | | | |  |  | |  |  |  |  |
| Fludeoxyglucose (F-18) | | | | 747014 | |  | |  | | | | | | |  |  | |  |  |  |  |
| Fludeoxyglucose (F-18) | | | | 747762 | |  | |  | | | | | | |  |  | |  |  |  |  |
| Fludeoxyglucose (F-18) | | | | 747810 | |  | |  | | | | | | |  |  | |  |  |  |  |
| Fludeoxyglucose (F-18) | | | | 746981 | |  | |  | | | | | | |  |  | |  |  |  |  |
| Fludeoxyglucose (F-18) | | | | 747025 | |  | |  | | | | | | |  |  | |  |  |  |  |
| Fludeoxyglucose (F-18) | | | | 746970 | |  | |  | | | | | | |  |  | |  |  |  |  |
|  |  | |  | | | |  | | | |  | | |  |  |  |  |  |  |  |  |
| **Table 11: nomenclature selection for computed tomography (CT)** | | | | | | |  | | | |  | | |  |  |  |  |  |  |  |  |
|  |  | |  | | | |  | | | |  | | |  |  |  |  |  |  |  |  |
| **code ambulatory** | **code hospitalisation** | | **dutch description** | | | | **french description** | | | | **creation date** | | | **suppression date** |  |  |  |  |  |  |  |
| ***CT (brain)*** | | | | | | | | | | | | | | |  |  |  |  |  |  |  |
| 458673 | 458684 | | Computergestuurde tomografie van de schedel met of zonder contrast, met registreren en clichés, minimum 10 coupes, voor het hele onderzoek | | | | Tomographie du crâne commandée par ordinateur, avec ou sans moyen de contraste, avec enregistrement et clichés, 10 coupes au minimum pour l'ensemble de l'examen | | | | 5/05/2016 | | |  |  |  |  |  |  |  |  |
| 458732 | 458743 | | Computergestuurde tomografie van de rotsbeenderen en/of sella torsica met of zonder contrastmiddel, met registreren en clichés, in een opeenvolgende reeks coupes, gelijk aan of minder dan 2 mm : minimum 20 coupes | | | | Tomographie des rochers et/ou de la selle turcique, commandée par ordinateur, avec ou sans moyen de contraste, avec enregistrement et clichés, dans une série successive de coupes égales ou inférieures à 2 mm : 20 coupes au minimum | | | | 31/12/1986 | | |  |  |  |  |  |  |  |  |
| 459675 | 459686 | | Computergestuurde tomografie (CT) met contrast van het faciaal massief | | | | Tomographie commandée par ordinateur (CT) avec moyen de contraste du massif facial | | | | 1/02/2012 | | |  |  |  |  |  |  |  |  |
| 459690 | 459701 | | Computergestuurde tomografie (CT) zonder contrast van het faciaal massief | | | | Tomographie commandée par ordinateur (CT) sans moyen de contraste du massif facial | | | | 1/06/2015 | | |  |  |  |  |  |  |  |  |
| ***CT (spine)**** | | | | | | | | | | | | | | |  |  |  |  |  |  |  |
| 457855** | 457866** | | Computergestuurde tomografie van een niveau in de vorm van een wervellichaam of een tussenwervelruimte, met of zonder contrastmiddel, minimum 6 coupes : - voor twee of meer niveaus van de lumbosacrale wervelzuil | | | | Tomographie commandée par ordinateur, d'un niveau sous forme d'un corps vertébral ou d'un espace intervertébral avec ou sans moyen de contraste, 6 coupes au minimum : - pour deux ou plusieurs niveaux du rachis thoracique | | | | 1/12/2018 | | |  |  |  |  |  |  |  |  |
| 457870 | 457881 | | Computergestuurde tomografie van een niveau in de vorm van een wervellichaam of een tussenwervelruimte, met of zonder contrastmiddel, minimum 6 coupes : - voor twee of meer niveaus van de lumbosacrale wervelzuil | | | | Tomographie commandée par ordinateur, d'un niveau sous forme d'un corps vertébral ou d'un espace intervertébral avec ou sans moyen de contraste, 6 coupes au minimum : - pour deux ou plusieurs niveaux du rachis lombosacré | | | | 1/12/2018 | | |  |  |  |  |  |  |  |  |
| 457892 | 457903 | | Computergestuurde tomografie van een niveau in de vorm van een wervellichaam of een tussenwervelruimte, met of zonder contrastmiddel, minimum 6 coupes : - voor een onderzoek van de volledige wervelzuil of voor een combinatie van twee van de onderzoeken van de cervicale, de thoracale of de lumbosacrale wervelzuil | | | | Tomographie commandée par ordinateur, d'un niveau sous forme d'un corps vertébral ou d'un espace intervertébral avec ou sans moyen de contraste, 6 coupes au minimum : - pour un examen du rachis entier ou pour une combinaison de deux des examens des rachis cervical, thoracique ou lombosacré | | | | 1/12/2018 | | |  |  |  |  |  |  |  |  |
| 458850 | 458861 | | Computergestuurde tomografie van een niveau in de vorm van een wervellichaam of een tussenwervelruimte, met of zonder contrastmiddel, minimum 6 coupes : - voor twee of meer niveaus van de cervicale wervelzuil | | | | Tomographie commandée par ordinateur, d'un niveau sous forme d'un corps vertébral ou d'un espace intervertébral avec ou sans moyen de contraste, 6 coupes au minimum : - pour deux ou plusieurs niveaux du rachis cervical | | | | 31/12/1986 | | |  |  |  |  |  |  |  |  |
| ** These codes cannot be cumulated within a 30-day period unless a justification is included in the patient medical file* | | | | | | | | | | | |  |  |  |  |  |  |  |  |  |  |
| ** The words "of thoracale of lumbosacrale" have been lifted (<12/2018: codes used for the full vertebral column or for parts of it, >=12/2018: codes only used for cervical vertebrae) | | | | | | | | | | | | |  |  |  |  |  |  |  |  |  |
