# Supplementary material for: Measuring the diagnostic management and follow‐up imaging for glioma patients across Belgian hospitals between 2016 and 2019
Source: Cancer Med. 2024 Oct 30;13(21):e70045. doi: 10.1002/cam4.70045 (PMC11523142; doi:10.1002/cam4.70045)
Supplement: Supplementary file 2 — Appendix S2. [file CAM4-13-e70045-s002.docx]

# appendix 2: Patient allocation algorithms

## **Centre of Biopsy**

The Centre of Biopsy was defined as the centre where the diagnostic biopsy was administered. Only glioma patients who received a diagnostic biopsy are, based on this algorithm, assigned to a centre.

A timeframe of 3 months before until 3 months after the date of incidence is considered. An additional requirement is that, in case a surgical resection took place, the biopsy must have taken place before that surgical resection.

The centre of biopsy will be considered for the following indicator(s):

- X02: Proportion of low grade glioma patients undergoing PET before diagnostic biopsy

Table 1.1: Centre of biopsy and patient distribution, all glioma who received a diagnostic biopsy, incidence years 2016-2019.

| Number of distinct centres of biopsy  N | Number of patients with centre of biopsy | | Number of patients by centre of biopsy | | | | | |
| --- | --- | --- | --- | --- | --- | --- | --- | --- |
|  | Known  N (%) | Unknown  N (%) | Mean | Min | Q1 | Median | Q3 | Max |
| 50 | 906 (100.0) | 0 (0.0) | 18.1 | 1 | 6 | 15 | 25 | 77 |

Remark:

For 4/906 patients for who a diagnostic biopsy was identified, this identification was based on information extracted from the pathology reports. Identification of the centre of biopsy is thereby based on IMA hosp data. The centre equals the centre where the patients were hospitalized around the biopsy date.

## **Centre of Surgical Resection**

The Centre of Surgical Resection was defined as the centre where the first surgical resection as treatment for the primary glioma was administered. Only glioma patients who received a surgical resection are, based on this algorithm, assigned to a centre.

A timeframe of 1 month before until 9 months after the date of incidence is considered. In case a diagnostic biopsy was first followed by chemo- and/or radiotherapy and then by a surgical resection, the surgical resection was not considered as treatment for the primary glioma.

The centre of surgical resection will be considered for the following indicator(s):

- F01: Proportion of high grade glioma patients undergoing postoperative MRI

Table 2.1: Centre of surgical resection and patient distribution, all glioma who received a surgical resection, incidence years 2016-2019.

| Number of known distinct centres of surgical resection  N | Number of patients with centre of surgical resection | | Number of patients by centre of surgical resection | | | | | |
| --- | --- | --- | --- | --- | --- | --- | --- | --- |
|  | Known  N (%) | Unknown  N (%) | Mean | Min | Q1 | Median | Q3 | Max |
| 56 | 2 210 (99.9) | 2 (0.1) | 38.8 | 1 | 12 | 31 | 53 | 160 |

Remark:

For 7/2 212 patients for who a surgical resection was identified, this identification was based on information extracted from the pathology reports.

- - For 5/7 patients, identification of the centre of surgical resection is based on IMA hosp data. The centre equals the centre where the patients were hospitalized around the surgical resection date.
  - For 2/7 patients, identification was not possible based on IMA hosp data.The centre of surgical resection stays unknown for these patients.

## **Centre of main treatment**

The centre of main treatment is the centre where the main oncological treatment was given. All patients are assigned to a centre of main treatment, even if the patient didn’t receive any active oncological treatment.

A timeframe of 1 month before until 9 months after the date of incidence is considered for defining the start of oncological treatment (surgery, chemo- or radiotherapy). A timeframe of 3 months before until 3 months after the date of incidence is considered for identification of a diagnostic biopsy.

The centre of main treatment will be considered for the following indicators:

- D01: Proportion of glioma patients with WHO performance score reported to BCR
- X01: Proportion of glioma patients discussed via MOC
- D03: Proportion of intracranial ependymoma patients who received (full) spine MRI
- F02: Proportion of low grade glioma patients undergoing at least two MRI’s in the first follow-up year
- F03: Proportion of high grade glioma undergoing at least three MRI’s in the first follow-up year
- F04: Proportion of ependymoma patients undergoing at least three MRI’s in the first follow-up year

To define the centre of main treatment, the following procedures were considered:

| Procedure type | Timeframe | Remark |
| --- | --- | --- |
| Surgical resection | 1 month before until 9 months after the date of incidence | In case a diagnostic biopsy was followed first by chemo- and/or radiotherapy and then by a surgical resection, the surgical resection was not considered as treatment for the primary glioma |
| Chemotherapy | If surgical resection was performed:  Neo-adj: from 1 month before the incidence date until the day before surgical resection  Adj: from the day of surgical resection until 12 months after surgical resection  If no surgical resection was performed:  From 1 month before until 9 months after the date of incidence | / |
| Radiotherapy | If surgical resection was performed:  Neo-adj: from 1 month before the incidence date until the day before surgical resection  Adj: from the day of surgical resection until 12 months after surgical resection  If no surgical resection was performed:  From 1 month before until 9 months after the date of incidence | / |
| Diagnostic biopsy | 3 months before until 3 months after the date of incidence | Only procedures before surgical resection were considered as a diagnostic biopsy |

This does not mean that a patient had to receive all listed procedures. If the patient underwent more than one procedure within the considered timeframe, the patient is assigned to the centre who performed the main oncological treatment (see table below).

The following algorithm was constructed to assign patients to a centre of main treatment:

| Rule | Description | All glioma N (%) |  |
| --- | --- | --- | --- |
| Overall | | 3 067 (100.0) |  |
| **In the presence of a surgical resection:** | | | |
| Priority 1 | A surgical resection with known centre was charged | 2 210 (72.1) |  |
| Priority 2 | A diagnostic biopsy with known centre was charged | 0 (0.0) |  |
| Priority 3 | Rest   - *Patient was allocated to the centre who delivered the record to the BCR* | 2 (0.1) |  |
| **In the absence of a surgical resection:** | | | |
| Priority 1 | Primary chemo- and/or radiotherapy was charged:   - *Only chemotherapy: centre of chemotherapy is known* - *Only radiotherapy: centre of radiotherapy is known* - *Both chemo- and radiotherapy; centre of both procedures is known* - *Both therapies were given in the same centre* - *Both therapies were given in different centres; patient is allocated to the centre of radiotherapy* | 66 (2.2)  43 (1.4)  277 (9.0)  111 (3.6) |  |
| Priority 2 | The patient was not treated by primary chemo- and or radiotherapy, but a diagnostic biopsy with a known centre was charged | 214 (7.0) |  |
| Priority 3 | The patient did not receive oncological treatment, nor a diagnostic biopsy   - *The record was delivered to the BCR by only one centre* - *The record was delivered to the BCR by more than one centre 🡪 centre stays unknown* | 136 (4.4)  8 (0.3) |  |

Table 3.1: Centers of main treatment and patient distribution, all glioma, incidence years 2016-2019.

| Number of known distinct centres of main treatment  N | Number of patients with centre of main treatment | | Number of patients by centre of main treatment | | | | | |
| --- | --- | --- | --- | --- | --- | --- | --- | --- |
|  | Known  N (%) | Unknown  N (%) | Mean | Min | Q1 | Median | Q3 | Max |
| 73 | 3 059 (99.7) | 8 (0.3) | 41.4 | 1 | 4 | 21 | 54 | 242 |

## Centre of biopsy or first treatment (in the absence of a biopsy)

The centre of biopsy or first treatment (in the absence of a biopsy) was defined as the centre where the diagnostic biopsy was performed. If the patient did not receive a diagnostic biopsy but received oncological treatment (surgical resection, chemo- and/or radiotherapy), the patient is allocated to the centre who gave the first oncological treatment. All glioma patients who received a biopsy or at least one type of oncological treatment were assigned to a centre of biopsy or first treatment (in the absence of a biopsy).

A timeframe of 3 months before until 3 months after the date of incidence is considered if the diagnosis was based on a diagnostic biopsy. A timeframe of 1 month before until 9 months after the date of incidence is considered for defining the start of oncological treatment (surgical resection, chemo- or radiotherapy).

The centre of biopsy or first treatment (in the absence of a biopsy) will be considered for the following indicator:

- D02: Proportion of glioma patients who underwent MRI before diagnostic biopsy (or before start of oncological treatment)

To define the centre of biopsy or first treatment (in the absence of a biopsy), the following procedures were considered:

| Procedure type | Timeframe | Remark |
| --- | --- | --- |
| Diagnostic biopsy | 3 months before until 3 months after the date of incidence | Only procedures before surgical resection were considered as a diagnostic biopsy |
| Surgical resection | 1 month before until 9 months after the date of incidence | In case a diagnostic biopsy was followed first by chemo- and/or radiotherapy and secondly by a surgical resection, the surgical resection was not withheld as treatment for the primary glioma |
| Chemotherapy | If surgical resection was performed:  Neo-adj: from 1 month before incidence data until the day before surgical resection  Adj: from the day of surgical resection until 12 months after surgical resection  If no surgical resection was performed:  From 1 month before until 9 months after the date of incidence | / |
| Radiotherapy | If surgical resection was performed:  Neo-adj: from 1 month before incidence date until the day before surgical resection  Adj: from the day of surgical resection until 12 months after surgical resection  If no surgical resection was performed:  From 1 month before until 9 months after the date of incidence | / |

Note: This does not mean that a patient had to receive all listed procedures. Procedures were prioritized as mentioned in the table below.

The following algorithm was constructed to allocate patients to the centre of biopsy or first treatment (in the absence of a biopsy):

| Rule | Description | Glioma who received a biopsy or oncological treatment  N = 2 923 (100%) |
| --- | --- | --- |
| Overall | |  |
| **In the presence of a diagnostic biopsy:** | | |
| Priority 1 | A diagnostic biopsy with known centre was charged | 906 (31.0) |
| **In the absence of a diagnostic biopsy, but in the presence of an oncological treatment:** | | |
| Priority 1 | Neo-adjuvant treatment was charged, centre is known:   - *Only chemotherapy* - *Only radiotherapy* - *Both chemo- and radiotherapy*   *Both types of procedures in same centre*  *Both types of procedures in different centre, on same day 🡪 patient is allocated to centre of radiotherapy* | *10 (0.3)*  *0 (0.0)*  *1 (0.0)*  *1 (0.0)* |
| Priority 2 | A surgical resection with known centre was charged | 1 976 (67.6) |
| Priority 3 | Primary chemo- and/or radiotherapy was charged:   - *Only chemotherapy: centre of chemotherapy is known* - *Only radiotherapy: centre of radiotherapy is known* - *Both chemo- and radiotherapy; centre of both procedures is known* - *Both therapies were given in the same centre* - *Both therapies were given in different centres*   *Radiotherapy started before chemotherapy 🡪 patient is allocated to centre of radiotherapy*  *Radiotherapy and chemotherapy started on the same day 🡪 patient is allocated to centre of radiotherapy* | *9 (0.3)*  *3 (0.1)*  *9 (0.3)*  *3 (0.1)*  *3 (0.1)* |
| Priority 4 | Unknown centre | 2 (0.1) |

Table 4.1: Centre of biopsy or first treatment (in the absence of a biopsy), glioma who received a biopsy and/or treatment, incidence years 2016-2019.

| Number of known distinct centres of biopsy or first treatment  N | Number of patients with centre of biopsy or first treatment | | Number of patients by centre of biopsy or first treatment | | | | | |
| --- | --- | --- | --- | --- | --- | --- | --- | --- |
|  | Known  N (%) | Unknown  N (%) | Mean | Min | Q1 | Median | Q3 | Max |
| 58 | 2 921 (99.9) | 2 (0.1) | 49.5 | 1 | 13 | 42 | 74 | 229 |
