# Supplementary material for: Measuring the diagnostic management and follow‐up imaging for glioma patients across Belgian hospitals between 2016 and 2019
Source: Cancer Med. 2024 Oct 30;13(21):e70045. doi: 10.1002/cam4.70045 (PMC11523142; doi:10.1002/cam4.70045)
Supplement: Supplementary file 3 — Appendix S3. [file CAM4-13-e70045-s003.docx]

# appendix 3: flowcharts and additional tables with subgroup analysis, sensitivity analysis and historical comparison

## Documented WHO performance status (DO1)

## Flowchart

WHO performance status reported to the BCR?

## Results

Table 1.1 – Proportion of glioma patients who have a WHO performance status reported to the BCR, by patient, tumour and treatment characteristics

| **Glioma** | | | |
| --- | --- | --- | --- |
|  | **Denominator** | **Numerator** | **Proportion (%)** |
| **Overall** | 3 067 | 2 738 | 89.3 |
| **Year of incidence** |  |  |  |
| 2016 | 752 | 664 | 88.3 |
| 2017 | 742 | 662 | 89.2 |
| 2018 | 749 | 671 | 89.6 |
| 2019 | 824 | 741 | 89.9 |
| **Age at time of diagnosis** |  |  |  |
| 18-29 years | 141 | 112 | 79.4 |
| 30-39 years | 219 | 188 | 85.8 |
| 40-49 years | 324 | 284 | 87.7 |
| 50-59 years | 550 | 496 | 90.2 |
| 60-69 years | 816 | 728 | 89.2 |
| 70-79 years | 696 | 640 | 92.0 |
| 80+ years | 321 | 290 | 90.3 |
| **Sex** |  |  |  |
| male | 1 838 | 1 651 | 89.8 |
| female | 1 229 | 1 087 | 88.4 |
| **Multiple tumors** |  |  |  |
| Absent | 2 764 | 2 467 | 89.3 |
| Present | 303 | 271 | 89.4 |
| **Diabetes** |  |  |  |
| absent | 2 717 | 2 431 | 89.5 |
| present | 350 | 307 | 87.7 |
| **Cardiovascular disease** |  |  |  |
| absent | 1 718 | 1 521 | 88.5 |
| present | 1 349 | 1 217 | 90.2 |
| **Respiratory disease** |  |  |  |
| absent | 2 835 | 2 530 | 89.2 |
| present | 232 | 208 | 89.7 |
| **Treatment scheme** |  |  |  |
| surgery | 476 | 405 | 85.1 |
| surgery<adjuvant | 1 736 | 1 574 | 90.7 |
| primary chemo and/or RT | 497 | 437 | 87.9 |
| no oncological treatment | 358 | 322 | 89.9 |

*<: prior to Source: BCR-IMA*

Table 1.2 – Sensitivity analyses: Proportion of glioma patients who have a WHO performance status reported to the BCR, by time period

| **Glioma** | | | |
| --- | --- | --- | --- |
|  | **Denominator** | **Numerator** | **Proportion (%)** |
| **Study period (2016-2019)** | 3 067 | 2 738 | 89.3 |
| **Comparison period 1 (2012-2015)** | 2 871 | 2 554 | 89.0 |
| **Comparison period 2 (2008-2011)** | 2 755 | 2 228 | 80.9 |

Source: BCR-IMA

## MULTIDISCIPLINARY TEAM MEETING PRIOR TO DEFINITIVE MANAGEMENT (X01)

## Flowchart

####

MDT meeting between 1 month before and 9 months after incidence date?

*.*

## Results

Table 2.1 – Proportion of glioma patients for whom a multidisciplinary team (MDT) meeting was charged within 1 month before until 9 months after incidence date, by patient, tumour and treatment characteristics

| **Glioma** | | | |
| --- | --- | --- | --- |
|  | **Denominator** | **Numerator** | **Proportion (%)** |
| **Overall** | 3 067 | 2 846 | 92.8 |
| **Year of incidence** |  |  |  |
| 2016 | 752 | 692 | 92.0 |
| 2017 | 742 | 682 | 91.9 |
| 2018 | 749 | 702 | 93.7 |
| 2019 | 824 | 770 | 93.4 |
| **Age at time of diagnosis** |  |  |  |
| 18-29 years | 141 | 129 | 91.5 |
| 30-39 years | 219 | 208 | 95.0 |
| 40-49 years | 324 | 310 | 95.7 |
| 50-59 years | 550 | 526 | 95.6 |
| 60-69 years | 816 | 763 | 93.5 |
| 70-79 years | 696 | 634 | 91.1 |
| 80+ years | 321 | 276 | 86.0 |
| **Sex** |  |  |  |
| male | 1 838 | 1 718 | 93.5 |
| female | 1 229 | 1 128 | 91.8 |
| **Multiple tumors** |  |  |  |
| absent | 2 764 | 2 572 | 93.1 |
| present | 303 | 274 | 90.4 |
| **Subtype glioma** |  |  |  |
| HGG | 2 523 | 2 338 | 92.7 |
| LGG | 330 | 310 | 93.9 |
| other | 214 | 198 | 92.5 |
| **Diabetes** |  |  |  |
| absent | 2 717 | 2 533 | 93.2 |
| present | 350 | 313 | 89.4 |
| **Cardiovascular disease** |  |  |  |
| absent | 1 718 | 1 611 | 93.8 |
| present | 1 349 | 1 235 | 91.5 |
| **Respiratory disease** |  |  |  |
| absent | 2 835 | 2 627 | 92.7 |
| present | 232 | 219 | 94.4 |
| **Treatment scheme** |  |  |  |
| surgery | 476 | 407 | 85.5 |
| surgery<adjuvant | 1 736 | 1 678 | 96.7 |
| primary chemo and/or RT | 497 | 471 | 94.8 |
| no oncological treatment | 358 | 290 | 81.0 |
| **Basis of diagnosis** |  |  |  |
| histology | 2 924 | 2 722 | 93.1 |
| technical (eg imaging) | 143 | 124 | 86.7 |

*<: prior to Source: BCR-IMA*

Table 2.2 – Sensitivity analyses: Proportion of glioma patients for whom a multidisciplinary team (MDT) meeting was charged within 1 month before until 9 months after incidence date, by time period

| **Glioma** | | | |
| --- | --- | --- | --- |
|  | **Denominator** | **Numerator** | **Proportion (%)** |
| **Study period (2016-2019)** | 3 067 | 2 846 | 92.8 |
| **Comparison period 1 (2012-2015)** | 2 871 | 2 518 | 87.7 |
| **Comparison period 2 (2008-2011)** | 2 755 | 2 066 | 75.0 |

*Source: BCR-IMA*

Table 2.3 – Sensitivity analyses: Proportion of glioma patients for whom a multidisciplinary team (MDT) meeting was charged, by time frame around incidence date

| **Glioma** | | | |
| --- | --- | --- | --- |
|  | **Denominator** | **Numerator** | **Proportion (%)** |
| **From 1 month before until 2 months after incidence date** | 3 067 | 2 676 | 87.3 |
| **From 1 month before until 3 months after incidence date** | 3 067 | 2 734 | 89.1 |
| **From 1 month before until 6 months after incidence date** | 3 067 | 2 820 | 91.9 |
| **From 1 month before until 9 months after incidence date (main indicator)** | 3 067 | 2 846 | 92.8 |
| **From 1 month before until 12 months after incidence date** | 3 067 | 2 856 | 93.1 |

Source: BCR-IMA

Table 2.4 – Sensitivity analysis: Proportion of glioma patients who received oncological treatment (surgical resection, chemo- and/or radiotherapy) for whom a multidisciplinary team (MDT) meeting was charged before start of treatment

| **Glioma** | | | |
| --- | --- | --- | --- |
|  | **Denominator** | **Numerator** | **Proportion (%)** |
| **MDT meeting charged within 1 month before incidence date until 9 months after incidence date*** | 3 067 | 2 846 | 92.8 |
| **MDT meeting charged within 1 month before incidence date until the day before start of first treatment**** |  |  |  |
| **Overal**l | 2 709 | 733 | 27.1 |
| **Treatment modality** |  |  |  |
| surgery | 476 | 76 | 16.0 |
| surgery<adjuvant | 1 736 | 246 | 14.2 |
| primary chemo and/or RT | 497 | 411 | 82.7 |

*<: prior to Source: BCR-IMA*

** Calculated for all glioma patients; ** Only calculated for glioma patients who received an oncological treatment*

## DIAGNOSTIC MRI (D02)

## 3.1 Flowchart

MRI prior to diagnostic biopsy?

Diagnostic biopsy?

MRI prior to start of oncological treatment?

Oncological treatment?

## Results

Table 3.1 – Proportion of glioma patients who underwent MRI (MRI brain or fMRI) before a diagnostic biopsy or before start of treatment in the absence of a diagnostic biopsy, by patient, tumour and treatment characteristics

| **Glioma** | | | |
| --- | --- | --- | --- |
|  | **Denominator** | **Numerator** | **Proportion (%)** |
| **Overall** | 2 923 | 2 616 | 89.5 |
| **Year of incidence** |  |  |  |
| 2016 | 718 | 640 | 89.1 |
| 2017 | 707 | 645 | 91.2 |
| 2018 | 720 | 643 | 89.3 |
| 2019 | 778 | 688 | 88.4 |
| **Age at time of diagnosis** |  |  |  |
| 18-29 years | 134 | 108 | 80.6 |
| 30-39 years | 213 | 184 | 86.4 |
| 40-49 years | 318 | 292 | 91.8 |
| 50-59 years | 537 | 493 | 91.8 |
| 60-69 years | 802 | 721 | 89.9 |
| 70-79 years | 669 | 602 | 90.0 |
| 80+ years | 250 | 216 | 86.4 |
| **Sex** |  |  |  |
| male | 1 772 | 1 587 | 89.6 |
| female | 1 151 | 1 029 | 89.4 |
| **Multiple tumors** |  |  |  |
| absent | 2 641 | 2 371 | 89.8 |
| present | 282 | 245 | 86.9 |
| **Subtype glioma** |  |  |  |
| HGG | 2 439 | 2 202 | 90.3 |
| LGG | 319 | 286 | 89.7 |
| other | 165 | 128 | 77.6 |
| **WHO - Performance Status** |  |  |  |
| 0 – Asymptomatic | 166 | 146 | 88.0 |
| 1 – Symptomatic but completely ambulatory | 1 840 | 1 660 | 90.2 |
| 2 – Symptomatic, <50% in bed during the day | 433 | 371 | 85.7 |
| 3 – Symptomatic, >50% in bed, but not bedbound | 130 | 112 | 86.2 |
| 4 – Bedbound | 35 | 31 | 88.6 |
| Missing | 319 | 296 | 92.8 |
| **Diabetes** |  |  |  |
| absent | 2 594 | 2 321 | 89.5 |
| present | 329 | 295 | 89.7 |
| **Cardiovascular disease** |  |  |  |
| absent | 1 668 | 1 491 | 89.4 |
| present | 1 255 | 1 125 | 89.6 |
| **Respiratory disease** |  |  |  |
| absent | 2 705 | 2 425 | 89.6 |
| present | 218 | 191 | 87.6 |
| **Treatment scheme** |  |  |  |
| surgery | 476 | 399 | 83.8 |
| surgery<adjuvant | 1 736 | 1 579 | 91.0 |
| primary chemo and/or RT | 497 | 459 | 92.4 |
| no oncological treatment | 214 | 179 | 83.6 |

*<: prior to Source: BCR-IMA*

Table 3.2 – Sensitivity analysis: Proportion of glioma patients who underwent MRI (MRI brain or fMRI) before a diagnostic biopsy or before start of treatment in the absence of a diagnostic biopsy, by time period

| **Glioma** | | | |
| --- | --- | --- | --- |
|  | **Denominator** | **Numerator** | **Proportion (%)** |
| **Study period (2016-2019)** | 2 923 | 2 616 | 89.5 |
| **Comparison period 1 (2012-2015)** | 2 727 | 2 500 | 91.7 |
| **Comparison period 2 (2008-2011)** | 2 640 | 2 353 | 89.1 |

Source: BCR-IMA

Table 3.3 – Sensitivity analysis: Proportion of glioma patients who underwent MRI (MRI brain or fMRI) before a diagnostic biopsy or before start of treatment in the absence of a diagnostic biopsy, by contrast administration

| **Glioma** | | | |
| --- | --- | --- | --- |
|  | **Denominator** | **Numerator** | **Proportion (%)** |
| **Overall** | 2 923 | 2 616 | 89.5 |
| **Contrast administration** |  |  |  |
| only MRI with contrast | 2 923 | 1 413 | 48.3 |
| only MRI without contrast | 2 923 | 179 | 6.1 |
| both MRI with and without contrast | 2 923 | 1 024 | 35.0 |

Source: BCR-IMA

Table 3.4 – Sensitivity analysis: Proportion of glioma patients who underwent MRI (MRI brain or fMRI) before a diagnostic biopsy or before start of treatment in the absence of a diagnostic biopsy, by type of MRI

| **Glioma** | | | |
| --- | --- | --- | --- |
|  | **Denominator** | **Numerator** | **Proportion (%)** |
| **Overall** | 2 923 | 2 616 | 89.5 |
| **Type of MRI** |  |  |  |
| only MRI brain | 2 923 | 2 357 | 80.6 |
| only fMRI | 2 923 | 41 | 1.4 |
| both MRI brain and fMRI | 2 923 | 218 | 7.5 |

Source: BCR-IMA

Table 3.5 – Sensitivity analysis: Proportion of glioma patients (subdivided based on grade) who underwent MRI (MRI brain or fMRI) before a diagnostic biopsy or before start of treatment in the absence of a diagnostic biopsy, by timeframe

| **Glioma** | | | | | | | | | |
| --- | --- | --- | --- | --- | --- | --- | --- | --- | --- |
|  | **HGG** | | | **LGG** | | | **Other** | | |
|  | **Denominator** | **Numerator** | **Proportion (%)** | **Denominator** | **Numerator** | **Proportion (%)** | **Denominator** | **Numerator** | **Proportion (%)** |
| **Max 4 weeks before biopsy/start of treatment** | 2 439 | 2 164 | 88.7 | 319 | 269 | 84.3 | 165 | 120 | 72.7 |
| **Max 6 weeks before biopsy/start of treatment (main QI)** | 2 439 | 2 202 | 90.3 | 319 | 286 | 89.7 | 165 | 128 | 77.6 |
| **Max 8 weeks before biopsy/start of treatment** | 2 439 | 2 209 | 90.6 | 319 | 293 | 91.8 | 165 | 137 | 83.0 |
| **Max 10 weeks before biopsy/start of treatment** | 2 439 | 2 216 | 90.9 | 319 | 296 | 92.8 | 165 | 141 | 85.5 |
| **Max 12 weeks before biopsy/start of treatment** | 2 439 | 2 219 | 91.0 | 319 | 299 | 93.7 | 165 | 144 | 87.3 |

Source: BCR-IMA

Table 3.6 – Sensitivity analysis: Proportion of glioma patients who underwent imaging before a diagnostic biopsy or before start of treatment in the absence of a diagnostic biopsy, by type of imaging

| **Glioma** | | | |
| --- | --- | --- | --- |
|  | **Denominator** | **Numerator** | **Proportion (%)** |
| **Overall** | 2 923 | 2 815 | 96.3 |
| **Type of imaging** |  |  |  |
| only MRI | 2 923 | 478 | 16.4 |
| only PET | 2 923 | 4 | 0.1 |
| only CT | 2 923 | 179 | 6.1 |
| MRI and PET | 2 923 | 115 | 3.9 |
| MRI and CT | 2 923 | 1 719 | 58.8 |
| PET and CT | 2 923 | 16 | 0.5 |
| MRI, PET and CT | 2 923 | 304 | 10.4 |

Source: BCR-IMA

## Amino acid PET incorporated in biopsy target (X02)

## Flowchart

Low grade?

Diagnostic biopsy?

PET prior to diagnostic biopsy?

## Results

Table 4.1 – Proportion of patients with low grade (grade 2) glioma in whom nuclear imaging with PET was performed before a diagnostic biopsy, by patient, tumour and treatment characteristics

| **Low grade glioma** | | | |
| --- | --- | --- | --- |
|  | **Denominator** | **Numerator** | **Proportion (%)** |
| **Overall** | 121 | 39 | 32.2 |
| **Year of incidence** |  |  |  |
| 2016 | 24 | 9 | 37.5 |
| 2017 | 24 | 8 | 33.3 |
| 2018 | 36 | 12 | 33.3 |
| 2019 | 37 | 10 | 27.0 |
| **Age at time of diagnosis** |  |  |  |
| 18-29 years | 10 | 4 | 40.0 |
| 30-39 years | 23 | 8 | 34.8 |
| 40-49 years | 28 | 12 | 42.9 |
| 50-59 years | 20 | 9 | 45.0 |
| 60-69 years | 21 | 4 | 19.0 |
| 70-79 years | 17 | 2 | 11.8 |
| 80+ years | 2 | 0 | 0.0 |
| **Sex** |  |  |  |
| male | 69 | 21 | 30.4 |
| female | 52 | 18 | 34.6 |
| **Multiple tumors** |  |  |  |
| absent | 113 | 35 | 31.0 |
| present | 8 | 4 | 50.0 |
| **WHO - Performance Status** |  |  |  |
| 0 – Asymptomatic | 16 | 7 | 43.8 |
| 1 – Symptomatic but completely ambulatory | 70 | 21 | 30.0 |
| 2 – Symptomatic, <50% in bed during the day | 16 | 4 | 25.0 |
| 3 – Symptomatic, >50% in bed, but not bedbound | 3 | 0 | 0.0 |
| Missing | 16 | 7 | 43.8 |
| **Diabetes** |  |  |  |
| absent | 114 | 36 | 31.6 |
| present | 7 | 3 | 42.9 |
| **Cardiovascular disease** |  |  |  |
| absent | 85 | 33 | 38.8 |
| present | 36 | 6 | 16.7 |
| **Respiratory disease** |  |  |  |
| absent | 114 | 36 | 31.6 |
| present | 7 | 3 | 42.9 |
| **Treatment scheme** |  |  |  |
| surgery | 13 | 2 | 15.4 |
| surgery<adjuvant | 19 | 3 | 15.8 |
| primary chemo and/or RT | 66 | 29 | 43.9 |
| no oncological treatment | 33 | 5 | 21.7 |

*<: prior to Source: BCR-IMA*

Table 4.2 – Sensitivity analysis: Proportion of patients with low grade (grade 2) glioma in whom nuclear imaging with PET was performed before a diagnostic biopsy, by time period

| **Low grade glioma** | | | |
| --- | --- | --- | --- |
|  | **Denominator** | **Numerator** | **Proportion (%)** |
| **Study period (2016-2019)** | 121 | 39 | 32.2 |
| **Comparison period 1 (2012-2015)** | 103 | 4 | 3.9 |
| **Comparison period 2 (2008-2011)** | 99 | 4 | 4.0 |

*Source: BCR-IMA*

Table 4.3 – Sensitivity analysis: Proportion of patients with low grade (grade 2) glioma in whom nuclear imaging with PET was performed before a diagnostic biopsy, by tracer administration

| **Low grade glioma** | | | |
| --- | --- | --- | --- |
|  | **Denominator** | **Numerator** | **Proportion (%)** |
| **Overall** | 121 | 39 | 32.2 |
| **Tracer administration** |  |  |  |
| only PET with amino acid tracer | 121 | 13 | 10.7 |
| only PET with glucose tracer | 121 | 5 | 4.1 |
| only PET without tracer | 121 | 16 | 13.2 |
| combination of PETs | 121 | 5 | 4.1 |

*Source: BCR-IMA*

Table 4.4 – Sensitivity analysis: Proportion of patients with low grade (grade 2) glioma in whom nuclear imaging with PET was performed before a diagnostic biopsy, by time frame

| **Low grade glioma** | | | |
| --- | --- | --- | --- |
|  | **Denominator** | **Numerator** | **Proportion (%)** |
| **Max 6 weeks before biopsy** | 121 | 35 | 28.9 |
| **Max 12 weeks before biopsy (main indicator)** | 121 | 39 | 32.2 |
| **Max 18 weeks before biopsy** | 121 | 40 | 33.1 |
| **Max 24 weeks before biopsy** | 121 | 41 | 33.9 |

*Source: BCR-IMA*

Table 4.5 – Sensitivity analysis: Proportion of patients with low grade (grade 2) glioma in whom imaging was performed before a diagnostic biopsy, by type of imaging

| **Glioma** | | | |
| --- | --- | --- | --- |
|  | **Denominator** | **Numerator** | **Proportion (%)** |
| **Overall** | 121 | 120 | 99.2 |
| **Type of imaging** |  |  |  |
| only PET | 121 | 0 | 0.0 |
| only MRI | 121 | 24 | 19.8 |
| only CT | 121 | 3 | 2.5 |
| PET and MRI | 121 | 12 | 9.9 |
| PET and CT | 121 | 1 | 0.8 |
| MRI and CT | 121 | 54 | 44.6 |
| PET, MRI and CT | 121 | 26 | 21.5 |

*Source: BCR-IMA*

## MRI FULL SPINE EPENDYMOMA (D03)

## Flowchart

(full) spine MRI from 1 month before until 6 weeks after incidence date?

## Results

Table 5.1 – Proportion of intracranial ependymoma patients who had a (full) spine MRI from 1 month before until 6 weeks after incidence date, by patient, tumour and treatment characteristics

| **Ependymoma** | | | |
| --- | --- | --- | --- |
|  | **Denominator** | **Numerator** | **Proportion (%)** |
| **Overall** | 39 | 17 | 43.6 |
| **Year of incidence** |  |  |  |
| 2016 | 8 | 3 | 37.5 |
| 2017 | 11 | 7 | 63.6 |
| 2018 | 9 | 2 | 22.2 |
| 2019 | 11 | 5 | 45.5 |
| **Age at time of diagnosis** |  |  |  |
| 30-39 years | 4 | 1 | 25.0 |
| 40-49 years | 6 | 2 | 33.3 |
| 50-59 years | 13 | 9 | 69.2 |
| 60-69 years | 12 | 5 | 41.7 |
| 70-79 years | 4 | 0 | 0.0 |
| **Sex** |  |  |  |
| male | 27 | 10 | 37.0 |
| female | 12 | 7 | 58.3 |
| **Multiple tumors** |  |  |  |
| absent | 32 | 14 | 43.8 |
| present | 7 | 3 | 42.9 |
| **WHO - Performance Status** |  |  |  |
| 0 – Asymptomatic | 1 | 1 | 100.0 |
| 1 – Symptomatic but completely ambulatory | 30 | 12 | 40.0 |
| 2 – Symptomatic, <50% in bed during the day | 1 | 0 | 0.0 |
| 3 – Symptomatic, >50% in bed, but not bedbound | 1 | 1 | 100.0 |
| Missing | 6 | 3 | 50.0 |
| **Diabetes** |  |  |  |
| absent | 33 | 16 | 48.5 |
| present | 6 | 1 | 16.7 |
| **Cardiovascular disease** |  |  |  |
| absent | 24 | 11 | 45.8 |
| present | 15 | 6 | 40.0 |
| **Respiratory disease** |  |  |  |
| absent | 37 | 17 | 45.9 |
| present | 2 | 0 | 0.0 |
| **Treatment scheme** |  |  |  |
| surgery | 18 | 5 | 27.8 |
| surgery<adjuvant | 17 | 10 | 58.8 |
| primary chemo and/or RT | 1 | 1 | 100.0 |
| no oncological treatment | 3 | 1 | 33.3 |

*<: prior to Source: BCR-IMA*

Table 5.2 – Sensitivity analysis: Proportion of intracranial ependymoma patients who had a (full) spine MRI from 1 month before until 6 weeks after incidence date, by time period

| **Ependymoma** | | | |
| --- | --- | --- | --- |
|  | **Denominator** | **Numerator** | **Proportion (%)** |
| **Study period (2016-2019)** | 39 | 17 | 43.6 |
| **Comparison period 1 (2012-2015)** | 53 | 27 | 50.9 |
| **Comparison period 2 (2008-2011)** | 44 | 22 | 50.0 |

*Source: BCR-IMA*

Table 5.3 – Sensitivity analysis: Proportion of intracranial ependymoma patients who had (full) spine MRI, by timeframe

| **Ependymoma** | | | |
| --- | --- | --- | --- |
|  | **Denominator** | **Numerator** | **Proportion (%)** |
| **From 1 month before until 4 weeks after incidence date** | 39 | 13 | 33.3 |
| **From 1 month before until 6 weeks after incidence date (main QI)** | 39 | 17 | 43.6 |
| **From 1 month before until 8 weeks after incidence date** | 39 | 18 | 46.2 |
| **From 1 month before until 10 weeks after incidence date** | 39 | 19 | 48.7 |
| **From 1 month before until 12 weeks after incidence date** | 39 | 19 | 48.7 |

*Source: BCR-IMA*

Table 5.4 – Sensitivity analysis: Proportion of intracranial ependymoma patients who had (full) spine imaging from 1 month before until 6 weeks after incidence date, by type of imaging

| **Ependymoma** | | | |
| --- | --- | --- | --- |
|  | **Denominator** | **Numerator** | **Proportion (%)** |
| **Overall** | 39 | 18 | 46.2 |
| **Type of imaging** |  |  |  |
| only MRI | 39 | 16 | 41.0 |
| only CT | 39 | 1 | 2.6 |
| both MRI and CT | 39 | 1 | 2.6 |

*Source: BCR-IMA*

## IMMEDIATE POSTOP MRI IN HGG (F01)

## 6.1 Flowchart

High grade?

Surgical resection?

PostoperativeMRI (max 3 days after surgical resection)?

## 6.2 Results

Table 6.1 – Proportion of patients with high grade (grade 3/4) glioma who had postoperative MRI (MRI brain or fMRI) within 3 days after surgical resection, by patient, tumour and treatment characteristics

| **High grade glioma** | | | |
| --- | --- | --- | --- |
|  | **Denominator** | **Numerator** | **Proportion (%)** |
| **Overall** | 1 836 | 890 | 48.5 |
| **Year of incidence** |  |  |  |
| 2016 | 454 | 208 | 45.8 |
| 2017 | 451 | 224 | 49.7 |
| 2018 | 449 | 224 | 49.9 |
| 2019 | 482 | 234 | 48.5 |
| **Age at time of diagnosis** |  |  |  |
| 18-29 years | 39 | 17 | 43.6 |
| 30-39 years | 90 | 50 | 55.6 |
| 40-49 years | 185 | 111 | 60.0 |
| 50-59 years | 364 | 182 | 50.0 |
| 60-69 years | 578 | 284 | 49.1 |
| 70-79 years | 429 | 180 | 42.0 |
| 80+ years | 151 | 66 | 43.7 |
| **Sex** |  |  |  |
| male | 1 127 | 537 | 47.6 |
| female | 709 | 353 | 49.8 |
| **Multiple tumors** |  |  |  |
| absent | 1 652 | 811 | 49.1 |
| present | 184 | 79 | 42.9 |
| **WHO - Performance Status** |  |  |  |
| 0 – Asymptomatic | 100 | 54 | 54.0 |
| 1 – Symptomatic but completely ambulatory | 1 207 | 610 | 50.5 |
| 2 – Symptomatic, <50% in bed during the day | 262 | 110 | 42.0 |
| 3 – Symptomatic, >50% in bed, but not bedbound | 73 | 31 | 42.5 |
| 4 – Bedbound | 25 | 9 | 36.0 |
| Missing | 169 | 76 | 45.0 |
| **Diabetes** |  |  |  |
| absent | 1 620 | 806 | 49.8 |
| present | 216 | 84 | 38.9 |
| **Cardiovascular disease** |  |  |  |
| absent | 1 014 | 526 | 51.9 |
| present | 822 | 364 | 44.3 |
| **Respiratory disease** |  |  |  |
| absent | 1 693 | 828 | 48.9 |
| present | 143 | 62 | 43.4 |
| **Treatment scheme** |  |  |  |
| surgery | 246 | 76 | 30.9 |
| surgery<adjuvant | 1 590 | 814 | 51.2 |

*<: prior to Source: BCR-IMA*

Table 6.2 – Sensitivity analysis: Proportion of patients with high grade (grade 3/4) glioma who had postoperative MRI (MRI brain or fMRI) within 3 days after surgical resection, by time period

| **High grade glioma** | | | |
| --- | --- | --- | --- |
|  | **Denominator** | **Numerator** | **Proportion (%)** |
| **Study period (2016-2019)** | 1 836 | 890 | 48.5 |
| **Comparison period 1 (2012-2015)** | 1 666 | 697 | 41.8 |
| **Comparison period 2 (2008-2011)** | 1 614 | 489 | 30.3 |

*Source: BCR-IMA*

Table 6.3 – Sensitivity analysis: Proportion of patients with high grade (grade 3/4) glioma who had postoperative MRI (MRI brain or fMRI), by timeframe after surgical resection

| **High grade glioma** | | | |
| --- | --- | --- | --- |
|  | **Denominator** | **Numerator** | **Proportion (%)** |
| **Max 2 days after surgical resection** | 1 836 | 718 | 39.1 |
| **Max 3 days after surgical resection (main QI)** | 1 836 | 890 | 48.5 |
| **Max 5 days after surgical resection** | 1 836 | 981 | 53.4 |
| **Max 7 days after surgical resection** | 1 836 | 1 017 | 55.4 |
| **Max 9 days after surgical resection** | 1 836 | 1 040 | 56.6 |

*Source: BCR-IMA*

Table 6.4 – Sensitivity analysis: Proportion of patients with high grade (grade 3/4) glioma who had postoperative MRI (MRI brain or fMRI) within 3 days after surgical resection, by type of MRI

| **High grade glioma** | | | |
| --- | --- | --- | --- |
|  | **Denominator** | **Numerator** | **Proportion (%)** |
| **Overall** | 1 836 | 890 | 48.5 |
| **By type of MRI** |  |  |  |
| only MRI brain | 1 836 | 889 | 48.4 |
| only fMRI | 1 836 | 1 | 0.1 |
| both MRI brain and fMRI | 1 836 | 0 | 0.0 |

*Source: BCR-IMA*

Table 6.5 – Sensitivity analysis: Proportion of patients with high grade (grade 3/4) glioma who had postoperative imaging within 3 days after surgical resection, by type of imaging

| **High grade glioma** | | | |
| --- | --- | --- | --- |
|  | **Denominator** | **Numerator** | **Proportion (%)** |
| **Overall** | 1 836 | 1 601 | 87.2 |
| **By type of imaging** |  |  |  |
| only MRI | 1 836 | 634 | 34.5 |
| only CT | 1 836 | 711 | 38.7 |
| both MRI and CT | 1 836 | 256 | 13.9 |

*Source: BCR-IMA*

Table 6.6 – Sensitivity analysis: Proportion of patients with high grade (grade 3/4) glioma who had postoperative imaging within 5 days after surgical resection. by type of imaging

| **High grade glioma** | | | |
| --- | --- | --- | --- |
|  | **Denominator** | **Numerator** | **Proportion (%)** |
| **Overall** | 1 836 | 1 666 | 90.7 |
| **By type of imaging** |  |  |  |
| only MRI | 1 836 | 647 | 35.2 |
| only CT | 1 836 | 685 | 37.3 |
| both MRI and CT | 1 836 | 334 | 18.2 |

*Source: BCR-IMA*

## FOLLOW-UP MRI LGG (F02)

## 7.1 Flowchart

≥ 1 year of follow-up in IMA-data?

≥ 2 MRI’s in the first follow-up year?

biopsy?

Low grade?

≥ 2 MRI’s in the first follow-up year?

Oncological treatment?

≥ 1 year of follow-up in IMA-data?

## Results

Table 7.1 – Proportion of patients with low grade (grade 2) glioma undergoing at least two MRI’s (MRI brain or fMRI) in the first year of follow-up, by patient, tumour and treatment characteristics

| **Low grade glioma** | | | |
| --- | --- | --- | --- |
|  | **Denominator** | **Numerator** | **Proportion (%)** |
| **Overall** | 277 | 260 | 93.9 |
| **Year of incidence** |  |  |  |
| 2016 | 66 | 61 | 92.4 |
| 2017 | 69 | 64 | 92.8 |
| 2018 | 70 | 69 | 98.6 |
| 2019 | 72 | 66 | 91.7 |
| **Age at time of diagnosis** |  |  |  |
| 18-29 years | 50 | 48 | 96.0 |
| 30-39 years | 79 | 76 | 96.2 |
| 40-49 years | 67 | 61 | 91.0 |
| 50-59 years | 42 | 41 | 97.6 |
| 60-69 years | 21 | 18 | 85.7 |
| 70-79 years | 16 | 14 | 87.5 |
| 80+ years | 2 | 2 | 100.0 |
| **Sex** |  |  |  |
| male | 159 | 148 | 93.1 |
| female | 118 | 112 | 94.9 |
| **Multiple tumors** |  |  |  |
| absent | 262 | 247 | 94.3 |
| present | 15 | 13 | 86.7 |
| **WHO - Performance Status** |  |  |  |
| 0 – Asymptomatic | 37 | 35 | 94.6 |
| 1 – Symptomatic but completely ambulatory | 169 | 160 | 94.7 |
| 2 – Symptomatic, <50% in bed during the day | 29 | 27 | 93.1 |
| 3 – Symptomatic, >50% in bed, but not bedbound | 2 | 2 | 100.0 |
| Missing | 40 | 36 | 90.0 |
| **Diabetes** |  |  |  |
| absent | 269 | 253 | 94.1 |
| present | 8 | 7 | 87.5 |
| **Cardiovascular disease** |  |  |  |
| absent | 236 | 225 | 95.3 |
| present | 41 | 35 | 85.4 |
| **Respiratory disease** |  |  |  |
| absent | 269 | 253 | 94.1 |
| present | 8 | 7 | 87.5 |
| **Treatment scheme** |  |  |  |
| surgery | 108 | 97 | 89.8 |
| surgery<adjuvant | 103 | 103 | 100.0 |
| primary chemo and/or RT | 49 | 47 | 95.9 |
| no oncological treatment | 17 | 13 | 76.5 |

*<: prior to Source: BCR-IMA*

Table 7.2 – Sensitivity analysis: Proportion of patients with low grade (grade 2) glioma undergoing at least two MRI’s (MRI brain or fMRI) in the first year of follow-up, by time period

| **Low grade glioma** | | | |
| --- | --- | --- | --- |
|  | **Denominator** | **Numerator** | **Proportion (%)** |
| **Study period (2016-2019)** | 277 | 260 | 93.9 |
| **Comparison period 1 (2012-2015)** | 247 | 230 | 93.1 |
| **Comparison period 2 (2008-2011)** | 218 | 195 | 89.4 |

*Source: BCR-IMA*

Table 7.3 – Sensitivity analysis: Proportion of patients with low grade (grade 2) glioma undergoing MRI’s (MRI brain or fMRI) in the first year of follow-up, by number of MRI’s

| **Low grade glioma** | | | |
| --- | --- | --- | --- |
|  | **Denominator** | **Numerator** | **Proportion (%)** |
| **At least 1 MRI in the first follow-up year** | 277 | 277 | 100.0 |
| **At least 2 MRI in the first follow-up year (main QI)** | 277 | 260 | 93.9 |
| **At least 3 MRI in the first follow-up year** | 277 | 195 | 70.4 |
| **At least 4 MRI in the first follow-up year** | 277 | 119 | 43.0 |

*Source: BCR-IMA*

Table 7.4 – Sensitivity analysis: Proportion of patients with low grade (grade 2) glioma undergoing at least two MRI’s (MRI brain or fMRI) during follow-up, by consecutive follow-up years

| **Low grade glioma** | | | |
| --- | --- | --- | --- |
|  | **Denominator** | **Numerator** | **Proportion (%)** |
| **At least 2 MRI in the first follow-up year (main QI)** | 277 | 260 | 93.9 |
| **At least 2 MRI in the second follow-up year** | 209 | 151 | 72.2 |
| **At least 2 MRI in the third follow-up year** | 141 | 81 | 57.4 |

*Source: BCR-IMA*

Table 7.5 – Background analysis: Patient characteristics of patients with low grade (grade 2) glioma who have or don’t have at least two MRI’s in the second or third follow-up year

| **Low grade glioma** | | | | | | | | |
| --- | --- | --- | --- | --- | --- | --- | --- | --- |
|  | **< 2 MRI in second**  **FU year (N=58)** | | **>= 2 MRI in the second FU year (N=151)** | | **< 2 MRI in third**  **FU year (N=60)** | | **>= 2 MRI in the third FU year (N=81)** | |
|  | **Nr** | **%** | **Nr** | **%** | **Nr** | **%** | **Nr** | **%** |
| **Age at time of diagnosis** |  |  |  |  |  |  |  |  |
| 18-29 years | 17 | 29.3 | 23 | 15.2 | 9 | 15.0 | 20 | 24.7 |
| 30-39 years | 13 | 22.4 | 48 | 31.8 | 16 | 26.7 | 21 | 25.9 |
| 40-49 years | 16 | 27.6 | 40 | 26.5 | 23 | 38.3 | 22 | 27.2 |
| 50-59 years | 4 | 6.9 | 23 | 15.2 | 3 | 5.0 | 11 | 13.6 |
| 60-69 years | 4 | 6.9 | 10 | 6.6 | 5 | 8.3 | 4 | 4.9 |
| 70-79 years | 3 | 5.2 | 6 | 4.0 | 4 | 6.7 | 2 | 2.5 |
| 80+ years | 1 | 1.7 | 1 | 0.7 | 0 | 0.0 | 1 | 1.2 |
| **Sex** |  |  |  |  |  |  |  |  |
| male | 35 | 60.3 | 89 | 58.9 | 30 | 50.0 | 57 | 70.4 |
| female | 23 | 39.7 | 62 | 41.1 | 30 | 50.0 | 24 | 29.6 |
| **Multiple tumors** |  |  |  |  |  |  |  |  |
| absent | 56 | 96.6 | 143 | 94.7 | 58 | 96.7 | 77 | 95.1 |
| present | 2 | 3.4 | 8 | 5.3 | 2 | 3.3 | 4 | 4.9 |
| **WHO - Performance Status** |  |  |  |  |  |  |  |  |
| 0 – Asymptomatic | 8 | 13.8 | 19 | 12.6 | 7 | 11.7 | 11 | 13.6 |
| 1 – Symptomatic but completely ambulatory | 34 | 58.6 | 97 | 64.2 | 36 | 60.0 | 53 | 65.4 |
| 2 – Symptomatic. <50% in bed during the day | 5 | 8.6 | 18 | 11.9 | 7 | 11.7 | 9 | 11.1 |
| 3 – Symptomatic. >50% in bed, but not bedbound | 0 | 0.0 | 1 | 0.7 | 0 | 0.0 | 0 | 0.0 |
| Missing | 11 | 19.0 | 16 | 10.6 | 10 | 16.7 | 8 | 9.9 |
| **Diabetes** |  |  |  |  |  |  |  |  |
| absent | 57 | 98.3 | 147 | 97.4 | 58 | 96.7 | 80 | 98.8 |
| present | 1 | 1.7 | 4 | 2.6 | 2 | 3.3 | 1 | 1.2 |
| **Respiratory disease** |  |  |  |  |  |  |  |  |
| absent | 56 | 96.6 | 146 | 96.7 | 60 | 100.0 | 77 | 95.1 |
| present | 2 | 3.4 | 5 | 3.3 | 0 | 0.0 | 4 | 4.9 |
| **Cardiovascular disease** |  |  |  |  |  |  |  |  |
| absent | 48 | 82.8 | 129 | 85.4 | 51 | 85.0 | 74 | 91.4 |
| present | 10 | 17.2 | 22 | 14.6 | 9 | 15.0 | 7 | 8.6 |
| **Treatment scheme** |  |  |  |  |  |  |  |  |
| surgery | 41 | 70.7 | 51 | 33.8 | 38 | 63.3 | 32 | 39.5 |
| surgery<adjuvant | 8 | 13.8 | 64 | 42.4 | 9 | 15.0 | 36 | 44.4 |
| primary chemo and/or RT | 3 | 5.2 | 27 | 17.9 | 6 | 10.0 | 9 | 11.1 |
| no oncological treatment | 6 | 10.3 | 9 | 6.0 | 7 | 11.7 | 4 | 4.9 |

*Source: BCR-IMA*

Table 7.6 – Sensitivity analysis: Proportion of patients with low grade (grade 2) glioma undergoing at least two MRI’s (MRI brain or fMRI) in the first year of follow-up. by type of MRI

| **Low grade glioma** | | | |
| --- | --- | --- | --- |
|  | **Denominator** | **Numerator** | **Proportion (%)** |
| **Overall** | 277 | 260 | 93.9 |
| **By type of MRI** |  |  |  |
| only MRI brain | 277 | 256 | 92.4 |
| only fMRI | 277 | 0 | 0.0 |
| both MRI brain and fMRI | 277 | 4 | 1.4 |

*Source: BCR-IMA*

Table 7.7 – Sensitivity analysis: Proportion of patients with diffuse astrocytoma undergoing at least two MRI’s (MRI brain or fMRI) in the first year of follow-up. by IDH molecular status

| **Low grade glioma** | | | |
| --- | --- | --- | --- |
|  | **Denominator** | **Numerator** | **Proportion (%)** |
| **Overall** | 112 | 106 | 94.6 |
| **IDH** |  |  |  |
| negative | 31 | 28 | 90.3 |
| positive | 58 | 57 | 98.3 |
| unknown | 23 | 21 | 91.3 |

**only tumors with an incidence 2017-2019 for which at least one protocol related to the primary tumour was available at the BCR were included*

*Source: BCR-IMA-annotated protocols*

## FOLLOW-UP MRI HGG (F03)

## Flowchart

≥ 1 year of follow-up in IMA-data?

≥ 3 MRI’s in the first follow-up year?

High grade?

biopsy?

Oncological treatment?

≥ 3 MRI’s in the first follow-up year?

≥ 1 year of follow-up in IMA-data?

## Results

Table 8.1 – Proportion of patients with high grade (grade 3/4) glioma undergoing at least three MRI’s (MRI brain or fMRI) in the first year of follow-up, by patient, tumour and treatment characteristics

| **High grade glioma** | | | |
| --- | --- | --- | --- |
|  | **Denominator** | **Numerator** | **Proportion (%)** |
| **Overall** | 1 059 | 953 | 90.0 |
| **Year of incidence** |  |  |  |
| 2016 | 257 | 225 | 87.5 |
| 2017 | 260 | 236 | 90.8 |
| 2018 | 258 | 234 | 90.7 |
| 2019 | 284 | 258 | 90.8 |
| **Age at time of diagnosis** |  |  |  |
| 18-29 years | 35 | 28 | 80.0 |
| 30-39 years | 88 | 77 | 87.5 |
| 40-49 years | 155 | 140 | 90.3 |
| 50-59 years | 252 | 232 | 92.1 |
| 60-69 years | 325 | 297 | 91.4 |
| 70-79 years | 175 | 156 | 89.1 |
| 80+ years | 29 | 23 | 79.3 |
| **Sex** |  |  |  |
| male | 662 | 591 | 89.3 |
| female | 397 | 362 | 91.2 |
| **Multiple tumors** |  |  |  |
| absent | 971 | 879 | 90.5 |
| present | 88 | 74 | 84.1 |
| **WHO - Performance Status** |  |  |  |
| 0 – Asymptomatic | 67 | 62 | 92.5 |
| 1 – Symptomatic but completely ambulatory | 738 | 671 | 90.9 |
| 2 – Symptomatic, <50% in bed during the day | 113 | 96 | 85.0 |
| 3 – Symptomatic, >50% in bed, but not bedbound | 26 | 24 | 92.3 |
| 4 – Bedbound | 11 | 11 | 100.0 |
| Missing | 104 | 89 | 85.6 |
| **Diabetes** |  |  |  |
| absent | 968 | 874 | 90.3 |
| present | 91 | 79 | 86.8 |
| **Cardiovascular disease** |  |  |  |
| absent | 696 | 625 | 89.8 |
| present | 363 | 328 | 90.4 |
| **Respiratory disease** |  |  |  |
| absent | 1 000 | 902 | 90.2 |
| present | 59 | 51 | 86.4 |
| **Treatment scheme** |  |  |  |
| surgery | 20 | 8 | 40.0 |
| surgery<adjuvant | 925 | 846 | 91.5 |
| primary chemo and/or RT | 111 | 98 | 88.3 |
| no oncological treatment | 3 | 1 | 33.3 |

*<: prior to Source: BCR-IMA*

Table 8.2 – Sensitivity analysis: Proportion of patients with high grade (grade 3/4) glioma undergoing at least three MRI’s (MRI brain or fMRI) in the first year of follow-up, by time period

| **High grade glioma** | | | |
| --- | --- | --- | --- |
|  | **Denominator** | **Numerator** | **Proportion (%)** |
| **Study period (2016-2019)** | 1 059 | 953 | 90.0 |
| **Comparison period 1 (2012-2015)** | 948 | 872 | 92.0 |
| **Comparison period 2 (2008-2011)** | 917 | 790 | 86.2 |

*Source: BCR-IMA*

Table 8.3 – Sensitivity analysis: Proportion of patients with high grade (grade 3/4) glioma undergoing MRI’s (MRI brain or fMRI) in the first year of follow-up, by number of MRI’s

| **High grade glioma** | | | |
| --- | --- | --- | --- |
|  | **Denominator** | **Numerator** | **Proportion (%)** |
| **At least 1 MRI in the first follow-up year** | 1 059 | 1 047 | 98.9 |
| **At least 2 MRI in the first follow-up year** | 1 059 | 1 031 | 97.4 |
| **At least 3 MRI in the first follow-up year (main QI)** | 1 059 | 953 | 90.0 |
| **At least 4 MRI in the first follow-up year** | 1 059 | 821 | 77.5 |

*Source: BCR-IMA*

Table 8.4 – Sensitivity analysis: Proportion of patients with high grade (grade 3/4) glioma undergoing at least three MRI’s (MRI brain or fMRI) during follow-up, by consecutive follow-up years

| **High grade glioma** | | | |
| --- | --- | --- | --- |
|  | **Denominator** | **Numerator** | **Proportion (%)** |
| **At least 3 MRI in the first follow-up year (main QI)** | 1 059 | 953 | 90.0 |
| **At least 3 MRI in the second follow-up year** | 445 | 331 | 74.4 |
| **At least 3 MRI in the third follow-up year** | 225 | 138 | 61.3 |

** Denominator decreases because requested time alive since start of follow-up increases*

*Source: BCR-IMA*

Table 8.5– Background analysis: Patient characteristics of patients with high grade (grade 3/4) glioma who have or don’t have at least three MRI in the second or third follow-up year

| **High grade glioma** | | | | | | | | |
| --- | --- | --- | --- | --- | --- | --- | --- | --- |
|  | **< 3 MRI in second**  **FU year (N=114)** | | **>= 3 MRI in the second FU year (N=331)** | | **< 3 MRI in third**  **FU year (N=87** | | **>= 3 MRI in the third**  **FU year (N=138)** | |
|  | **Nr** | **%** | **Nr** | **%** | **Nr** | **%** | **Nr** | **%** |
| **Age at time of diagnosis** |  |  |  |  |  |  |  |  |
| 18-29 years | 5 | 4.4 | 19 | 5.7 | 8 | 9.2 | 7 | 5.1 |
| 30-39 years | 16 | 14.0 | 40 | 12.1 | 19 | 21.8 | 24 | 17.4 |
| 40-49 years | 20 | 17.5 | 56 | 16.9 | 14 | 16.1 | 24 | 17.4 |
| 50-59 years | 22 | 19.3 | 75 | 22.7 | 13 | 14.9 | 37 | 26.8 |
| 60-69 years | 29 | 25.4 | 95 | 28.7 | 26 | 29.9 | 32 | 23.2 |
| 70-79 years | 20 | 17.5 | 42 | 12.7 | 6 | 6.9 | 12 | 8.7 |
| 80+ years | 2 | 1.8 | 4 | 1.2 | 1 | 1.1 | 2 | 1.4 |
| **Sex** |  |  |  |  |  |  |  |  |
| male | 67 | 58.8 | 199 | 60.1 | 48 | 55.2 | 80 | 58.0 |
| female | 47 | 41.2 | 132 | 39.9 | 39 | 44.8 | 58 | 42.0 |
| **Multiple tumors** |  |  |  |  |  |  |  |  |
| absent | 108 | 94.7 | 310 | 93.7 | 82 | 94.3 | 134 | 97.1 |
| present | 6 | 5.3 | 21 | 6.3 | 5 | 5.7 | 4 | 2.9 |
| **WHO - Performance Status** |  |  |  |  |  |  |  |  |
| 0 – Asymptomatic | 8 | 7.0 | 16 | 4.8 | 2 | 2.3 | 7 | 5.1 |
| 1 – Symptomatic but completely ambulatory | 71 | 62.3 | 233 | 70.4 | 52 | 59.8 | 108 | 78.3 |
| 2 – Symptomatic, <50% in bed during the day | 13 | 11.4 | 40 | 12.1 | 11 | 12.6 | 10 | 7.2 |
| 3 – Symptomatic, >50% in bed, but not bedbound | 2 | 1.8 | 5 | 1.5 | 1 | 1.1 | 1 | 0.7 |
| 4 – Bedbound | 1 | 0.9 | 4 | 1.2 | 2 | 2.3 | 1 | 0.7 |
| Missing | 19 | 16.7 | 33 | 10.0 | 19 | 21.8 | 11 | 8.0 |
| **Diabetes** |  |  |  |  |  |  |  |  |
| absent | 106 | 93.0 | 315 | 95.2 | 82 | 94.3 | 131 | 94.9 |
| present | 8 | 7.0 | 16 | 4.8 | 5 | 5.7 | 7 | 5.1 |
| **Respiratory disease** |  |  |  |  |  |  |  |  |
| absent | 109 | 95.6 | 312 | 94.3 | 83 | 95.4 | 133 | 96.4 |
| present | 5 | 4.4 | 19 | 5.7 | 4 | 4.6 | 5 | 3.6 |
| **Cardiovascular disease** |  |  |  |  |  |  |  |  |
| absent | 82 | 71.9 | 242 | 73.1 | 63 | 72.4 | 112 | 81.2 |
| present | 32 | 28.1 | 89 | 26.9 | 24 | 27.6 | 26 | 18.8 |
| **Treatment scheme** |  |  |  |  |  |  |  |  |
| surgery | 8 | 7.0 | 5 | 1.5 | 8 | 9.2 | 2 | 1.4 |
| surgery<adjuvant | 93 | 81.6 | 289 | 87.3 | 72 | 82.8 | 118 | 85.5 |
| primary chemo and/or RT | 12 | 10.5 | 36 | 10.9 | 6 | 6.9 | 18 | 13.0 |
| no oncological treatment | 1 | 0.9 | 1 | 0.3 | 1 | 1.1 | 0 | 0.0 |

*<: prior to Source: BCR-IMA*

Table 8.6– Sensitivity analysis: Proportion of patients with high grade (grade 3/4) glioma undergoing at least three MRI’s (MRI brain or fMRI) in the first year of follow-up, by type of MRI

| **High grade glioma** | | | |
| --- | --- | --- | --- |
|  | **Denominator** | **Numerator** | **Proportion (%)** |
| **Overall** | 1 059 | 953 | 90.0 |
| **By type of MRI** |  |  |  |
| only MRI brain in the first follow-up year | 1 059 | 918 | 86.7 |
| only fMRI in the first follow-up year | 1 059 | 0 | 0.0 |
| both MRI brain and fMRI in the first follow-up year | 1 059 | 35 | 3.3 |

*Source: BCR-IMA*

## FOLLOW-UP EPENDYMOMA (F04)

## Flowchart

≥ 1 year of follow-up in IMA-data?

≥ 3 MRI’s in the first follow-up year?

biopsy?

Ependymoma?

Oncological treatment?

≥ 3 MRI’s in the first follow-up year?

≥ 1 year of follow-up in IMA-data?

## Results

Table 9.1 – Proportion of ependymoma patients undergoing at least three MRI’s (MRI brain or fMRI) in the first year of follow-up, by patient, tumour and treatment characteristics

| **Ependymoma** | | | |
| --- | --- | --- | --- |
|  | **Denominator** | **Numerator** | **Proportion (%)** |
| **Overall** | 34 | 25 | 73.5 |
| **Year of incidence** |  |  |  |
| 2016 | 6 | 5 | 83.3 |
| 2017 | 10 | 7 | 70.0 |
| 2018 | 9 | 6 | 66.7 |
| 2019 | 9 | 7 | 77.8 |
| **Age at time of diagnosis** |  |  |  |
| 30-39 years | 4 | 3 | 75.0 |
| 40-49 years | 6 | 5 | 83.3 |
| 50-59 years | 12 | 10 | 83.3 |
| 60-69 years | 10 | 6 | 60.0 |
| 70-79 years | 2 | 1 | 50.0 |
| **Sex** |  |  |  |
| male | 23 | 18 | 78.3 |
| female | 11 | 7 | 63.6 |
| **Multiple tumors** |  |  |  |
| absent | 28 | 20 | 71.4 |
| present | 6 | 5 | 83.3 |
| **WHO - Performance Status** |  |  |  |
| 0 – Asymptomatic | 1 | 0 | 0.0 |
| 1 – Symptomatic but completely ambulatory | 25 | 17 | 68.0 |
| 2 – Symptomatic, <50% in bed during the day | 1 | 1 | 100.0 |
| 3 – Symptomatic, >50% in bed, but not bedbound | 1 | 1 | 100.0 |
| Missing | 6 | 6 | 100.0 |
| **Diabetes** |  |  |  |
| absent | 28 | 20 | 71.4 |
| present | 6 | 5 | 83.3 |
| **Cardiovascular disease** |  |  |  |
| absent | 22 | 17 | 77.3 |
| present | 12 | 8 | 66.7 |
| **Respiratory disease** |  |  |  |
| absent | 32 | 24 | 75.0 |
| **present** | 2 | 1 | 50.0 |
| **Treatment scheme** |  |  |  |
| surgery | 15 | 8 | 53.3 |
| surgery<adjuvant | 17 | 16 | 94.1 |
| primary chemo and/or RT | 1 | 1 | 100.0 |
| no oncological treatment | 1 | 0 | 0.0 |

*<: prior to Source: BCR-IMA*

Table 9.2 – Sensitivity analysis: Proportion of ependymoma patients undergoing at least three MRI’s (MRI brain or fMRI) in the first year of follow-up, by time period

| **Ependymoma** | | | |
| --- | --- | --- | --- |
|  | **Denominator** | **Numerator** | **Proportion (%)** |
| **Study period (2016-2019)** | 34 | 25 | 73.5 |
| **Comparison period 1 (2012-2015)** | 39 | 26 | 66.7 |
| **Comparison period 2 (2008-2011)** | 32 | 14 | 43.8 |

*Source: BCR-IMA*

Table 9.3 – Sensitivity analysis: Proportion of ependymoma patients undergoing MRI’s (MRI brain or fMRI) in the first year of follow-up, by number of MRI’s

| **Ependymoma** | | | |
| --- | --- | --- | --- |
|  | **Denominator** | **Numerator** | **Proportion (%)** |
| **At least 1 MRI in the first follow-up year** | 34 | 32 | 94.1 |
| **At least 2 MRI in the first follow-up year** | 34 | 31 | 91.2 |
| **At least 3 MRI in the first follow-up year (main QI)** | 34 | 25 | 73.5 |
| **At least 4 MRI in the first follow-up year** | 34 | 14 | 41.2 |

*Source: BCR-IMA*

Table 9.4 – Sensitivity analysis: Proportion of ependymoma patients undergoing at least three MRI’s (MRI brain or fMRI) during follow-up, by prolonging the first follow-up year

| **Ependymoma** | | | |
| --- | --- | --- | --- |
|  | **Denominator** | **Numerator** | **Proportion (%)** |
| **At least 3 MRI in the first 365 days (main QI)** | 34 | 25 | 73.5 |
| **At least 3 MRI in the first 375 days** | 34 | 25 | 73.5 |
| **At least 3 MRI in the first 385 days** | 34 | 25 | 73.5 |
| **At least 3 MRI in the first 395 days** | 34 | 26 | 76.5 |

*Source: BCR-IMA*

Table 9.5 – Sensitivity analysis: Proportion of ependymoma patients undergoing at least three MRI’s (MRI brain or fMRI) during follow-up, by consecutive follow-up years

| **Ependymoma** | | | |
| --- | --- | --- | --- |
|  | **Denominator** | **Numerator** | **Proportion (%)** |
| **At least 3 MRI in the first follow-up year (main QI)** | 34 | 25 | 73.5 |
| **At least 3 MRI in the second follow-up year** | 25 | 6 | 24.0 |
| **At least 3 MRI in the third follow-up year** | 16 | 1 | 6.3 |

** Denominator decreases because requested time alive since start of follow-up increases*

*Source: BCR-IMA*

Table 9.6 – Sensitivity analysis: Proportion of ependymoma patients undergoing at least three MRI’s (MRI brain or fMRI) in the first year of follow-up, by type of MRI

| **Ependymoma** | | | |
| --- | --- | --- | --- |
|  | **Denominator** | **Numerator** | **Proportion (%)** |
| **Overall** | 34 | 25 | 73.5 |
| **By type of MRI** |  |  |  |
| only MRI brain in the first follow-up year | 34 | 25 | 73.5 |
| only fMRI in the first follow-up year | 34 | 0 | 0.0 |
| both MRI brain and fMRI in the first follow-up year | 34 | 0 | 0.0 |

*Source: BCR-IMA*
